# Structural and functional analysis of the Nipah virus polymerase complex

**DOI:** 10.1016/j.cell.2024.12.021

**Published:** 2025-02-06

**Authors:** Side Hu, Heesu Kim, Pan Yang, Zishuo Yu, Barbara Ludeke, Shawna Mobilia, Junhua Pan, Margaret Stratton, Yuemin Bian, Rachel Fearns, Jonathan Abraham

**Affiliations:** 1 Department of Microbiology, Blavatnik Institute, Harvard Medical School, Boston, MA, USA; 2 Department of Virology, Immunology & Microbiology, Boston University Chobanian & Avedisian School of Medicine, Boston, MA, USA; 3 Biomedical Research Institute and School of Life and Health Sciences, Hubei University of Technology, Wuhan, China; 4 Department of Biochemistry and Molecular Biology, University of Massachusetts, Amherst, MA, USA; 5 School of Medicine, Shanghai University, Shanghai, China; 6 Department of Medicine, Division of Infectious Diseases, Brigham & Women’s Hospital, Boston, MA, USA; 7 Center for Integrated Solutions in Infectious Diseases, Broad Institute of Harvard and MIT, Cambridge, MA, USA; 8 Howard Hughes Medical Institute, Boston, MA, USA

**Keywords:** Nipah virus, RNA virus, polymerase, emerging viruses, RNA replication, RNA transcription

## Abstract

Nipah virus (NiV) is a bat-borne, zoonotic RNA virus that is highly pathogenic in humans. The NiV polymerase, which mediates viral genome replication and mRNA transcription, is a promising drug target. We determined the cryoelectron microscopy (cryo-EM) structure of the NiV polymerase complex, comprising the large protein (L) and phosphoprotein (P), and performed structural, biophysical, and in-depth functional analyses of the NiV polymerase. The L protein assembles with a long P tetrameric coiled-coil that is capped by a bundle of ⍺-helices that we show are likely dynamic in solution. Docking studies with a known L inhibitor clarify mechanisms of antiviral drug resistance. In addition, we identified L protein features that are required for both transcription and RNA replication and mutations that have a greater impact on RNA replication than on transcription. Our findings have the potential to aid in the rational development of drugs to combat NiV infection.

## Highlights

• Cryo-EM structure of the NiV L-P complex determined
• Docking studies with an inhibitor clarify mechanisms of intrinsic NiV L resistance
• Palm insert, zinc fingers, and P4 extension are critical for NiV L activity
• Intrusion loop plays an essential role in RNA replication

## Graphical abstract

**Figure:**
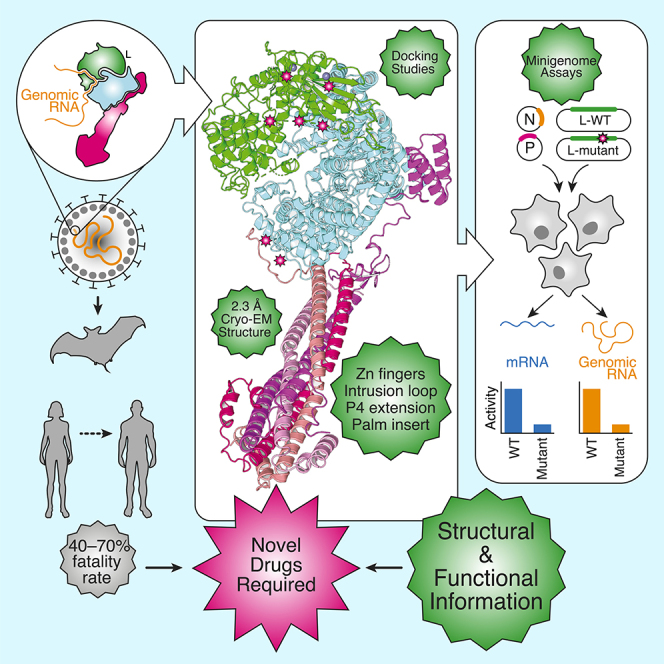

## Teaser

The cryo-EM structure and mutational analysis of the Nipah virus polymerase complex identify features critical for RNA replication and transcription with the potential to aid in the development of antivirals.

## Introduction

Nipah virus (NiV) is an enveloped RNA virus in the family *Paramyxoviridae* of the order *Mononegavirales*, the non-segmented negative-strand RNA viruses (nsNSVs).^1^ In humans, NiV infection can lead to respiratory illness or encephalitis with a fatality rate ranging from 40% to 70%.^2^ Since the identification of NiV in Malaysia in 1998, spillover events from bats into humans have occurred almost annually in Bangladesh, and NiV has also caused outbreaks in India and in the Philipines.^3^^,^^4^^,^^5^ Although prior outbreaks have been limited in size, NiV may have pandemic potential because infected individuals can be asymptomatic, and the virus can be transmitted from person to person with droplet-based transmission through coughing.^4^ There is no vaccine or antiviral against NiV infection, highlighting a gap in public health preparedness. For these reasons, NiV is on the World Health Organization Research & Development Blueprint list of priority diseases for which there is an urgent need for accelerated research and countermeasure development.

The NiV genome is a single strand of negative-sense RNA that is transcribed and replicated by the viral polymerase. During transcription, the polymerase generates capped and polyadenylated monocistronic mRNAs, corresponding to each of the viral genes.^6^^,^^7^^,^^8^^,^^9^ During replication it produces full-length, encapsidated replicative RNAs, which are uncapped and lack a poly A tail.^7^^,^^8^^,^^9^ The NiV polymerase comprises the large protein (L) and phosphoprotein (P).^10^^,^^11^ By analogy with the polymerases of other nsNSVs, the L protein comprises five domains: the RNA-dependent RNA polymerase (RdRp) domain, the capping domain (CAP), the connector domain (CD), the methyltransferase (MTase) domain, and the C-terminal domain (CTD).^7^^,^^8^^,^^9^^,^^12^ The P protein serves as an adaptor that allows the polymerase to associate both with the encapsidated ribonucleoprotein (RNP) template and with soluble nucleoprotein (N) protein that is required to encapsidate replicative RNA as it is synthesized.^7^^,^^8^^,^^9^ P contains an intrinsically disordered N-terminal domain (NTD), a central oligomerization domain (OD), and a C-terminal X domain (XD). The CTD of P binds the RNP template, and the NTD of P binds soluble N protein for encapsidation.^13^^,^^14^^,^^15^^,^^16^

Although several nsNSV polymerase structures are available, how the features identified within them facilitate different polymerase functions is generally poorly defined, and it is not fully understood how the polymerase is regulated to “switch on” and “switch off” the different enzymatic activities that are required for mRNA transcription versus genome replication.^17^ More complete analyses of the relationship between polymerase structure and function are necessary to clarify how the polymerase transitions between different enzymatic activities and produces different RNA products. Additionally, understanding the functional properties of key features will help identify those that could be leveraged as drug targets.

Here, we determined the 2.3 Å cryoelectron microscopy (cryo-EM) structure of the NiV L-P complex, which reveals how tetrameric P interacts with L. Structure-function analyses using single-step and multi-cycle NiV minigenome assays identify features of the polymerase that are required for transcription and/or genome replication, providing information that has the potential to aid rational design of antiviral molecules against NiV.

## Results

### Expression and purification of NiV L-P

**Figure 1:**
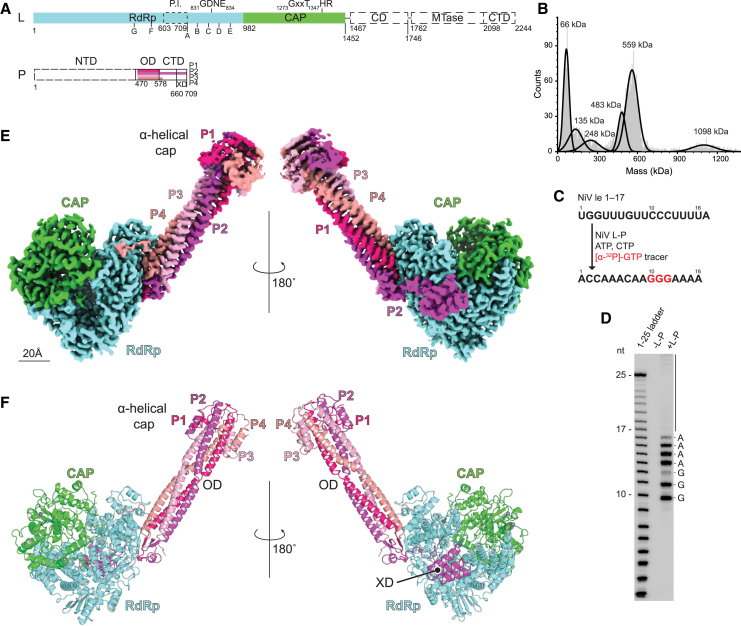
Structure of the NiV L-P complex (A) Domain organization of the NiV L and P proteins. The regions of tetrameric P protein that could be resolved vary in length depending on the protomer (P1–P4), as indicated. P NTD, OD, CTD, and XD boundaries are indicated based on established boundaries.^13^ (B) Mass photometry analysis of purified NiV L-P complex. The 559 kDa peak likely represents the L-P_4_ complex, the 483 kDa peak is consistent with an L-P_3_ complex, the 248 kDa peak may correspond to the L protein alone, and the 1098 kDa peak may represent L-P_4_ dimers. The 135 kDa and 66 kDa peaks may represent degradation products or contaminants. This experiment was performed twice, with representative data shown. (C) Workflow diagram of the RNA synthesis assay. The template is the leader (le) sequence of NiV, nucleotides 1–17. The GTP tracer and its incorporation in the product are in red. (D) RNA synthesis assay with radioactive products migrated on a denaturing polyacrylamide gel. nt, nucleotide. This experiment was performed four times with two different L-P preparations, with representative data shown. Note that some product bands migrated as doublets, and products longer than expected were also generated (indicated with a vertical line). These are likely generated by polymerase stuttering on the template. See also Figure S1B. (E) Cryo-EM density map of the NiV L-P complex with L domains and P protomers colored as indicated. Two views are shown. (F) NiV L-P complex with L domains and P protomers colored as indicated. See also Figure S1.

**Figure S1:**
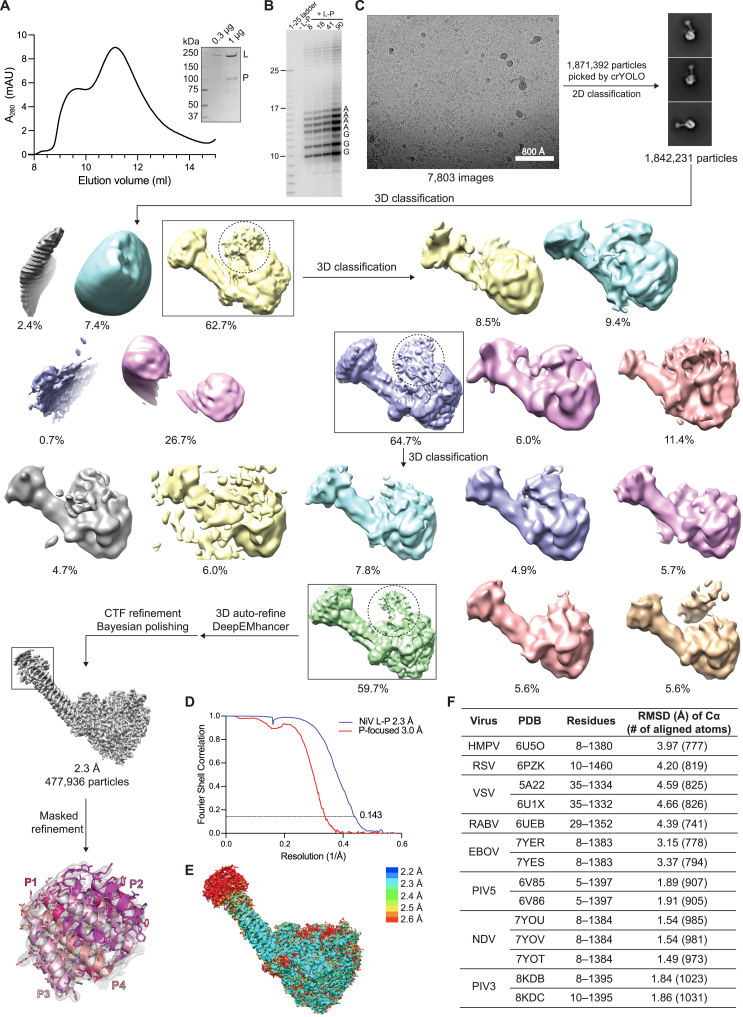
Purification and cryo-EM reconstruction of NiV L-P, activity assay, and structural alignment with other nsNSV L proteins, related to Figure 1 (A) Size exclusion chromatography profile of NiV L-P complex. The complex has a retention volume of ∼11 mL. A sample obtained from the 11 mL peak fraction was examined using SDS-PAGE, with a Coomassie-stained gel shown (inset). NiV L has a molecular weight of ∼260 kDa. NiV P has a molecular weight of ∼80 kDa but has an apparent size that is larger than its molecular weight (∼100 kDa). (B) RNA synthesis activity assay with radioactive products migrated on a denaturing polyacrylamide gel, as described for Figures 1C and 1D. Different amounts of polymerase complexes were used in this experiment as indicated (values indicate nM amounts of L). This analysis clearly shows products that are longer than the input template. Based on the observed migration patterns of the longer products (with incremental single nucleotide additions) and prior findings that nsNSV polymerases are prone to reiterative stuttering,^11^^,^^71^ we conclude that these additional bands are due to polymerase stuttering on U tracts within the promoter, although further experiments are required to show this conclusively. (C) Workflow used for cryo-EM data processing of the NiV L-P complex. Low-resolution features in 3D volumes consistent with the presence of flexible portions of the L C-terminal globular domains (CD, MTase, and CTD) are indicated with a dashed circled. (D) Fourier shell correlation (FSC) curves. The threshold used to estimate the resolution is 0.143. (E) Local resolution estimates of the NiV L-P complex obtained using ResMap.^72^ (F) Structural alignment of NiV L (residues 5–1463) with the L proteins of the indicated nsNSVs calculated over C-⍺ positions. The alignment was performed using PyMOL. RMSD, root-mean-square deviation.

We co-expressed full-length NiV L and P in insect cells (Figure 1A), purified L-P complexes, and analyzed samples of purified complexes by SDS-PAGE analysis (Figure S1A) and liquid chromatography-mass spectrometry (LC-MS/MS; Data S1). Analysis of samples by mass photometry revealed a major peak of 559 kDa, which likely represents L bound to four copies of P (L-P_4_ complex) (Figure 1B). The L-P complex was active in an *in vitro* RNA synthesis assay using an oligonucleotide RNA template containing the NiV promoter sequence and nucleoside triphosphates (NTPs) (Figures 1C, 1D, and S1B).

### Structure of the NiV L-P complex

We used single-particle cryo-EM to determine the structure of the NiV L-P complex to a global resolution of 2.3 Å (Figures S1C–S1E; Table S1). NiV L-P is shaped like a tobacco pipe, with the stem formed by the tetrameric P OD (Figures 1E and 1F). We observed interpretable density for L residues 5–1463, encompassing the RdRp and CAP domains, and for the P tetramer (P1–P4), of which we could observe different lengths for individual protomers (P1: residues 479–584, P2: 479–708, P3: 477–579, and P4: 479–596) (Figure 1A). Although we observed low-resolution features in 3D volumes during the earlier steps of data processing that are consistent with the presence of the L CD, MTase domain, and CTD, these features were lost during additional steps of data processing (Figure S1C). Therefore, like in the structures of the L proteins of respiratory syncytial virus (RSV) and human metapneumovirus (HMPV) (*Pneumoviridae*), the three L C-terminal globular domains in NiV L are probably too flexible to be visualized by cryo-EM^18^^,^^19^^,^^20^^,^^21^^,^^22^^,^^23^ (Data S2). For the RdRp and CAP domains that could be resolved, root-mean-square deviation values based on the Cα ranged from 1.7 to 4.7 Å between NiV and other nsNSV L proteins (Figure S1F), indicating structural conservation, consistent with the generally similar transcription and replication mechanisms of nsNSVs.

In the cryo-EM map, the N-terminal end of the OD of the NiV P tetramer is capped with a mushroom-shaped density (Figures 1E, S1C, and S1E). Focused refinement on this region allowed us to resolve individual ⍺-helices, allowing us to model the region based on a crystal structure of the NiV P OD.^15^ The ⍺-helices form a bundle that folds back onto the outer aspects of the coiled-coil core (Figure 1F), a feature observed in crystal structures of the NiV and Sendai virus (SeV) (paramyxovirus) phosphoproteins.^15^^,^^24^^,^^25^ These prior studies relied on truncated P constructs comprising only the OD, rather than full-length P.^15^^,^^24^^,^^25^ Observing the mushroom-shaped cap structure in the NiV L-P complex reveals that this feature is present when full-length P associates with L without the potential conformational biases that could have been introduced by crystallization of P OD in isolation.

### Features of the NiV L protein

**Figure 2:**
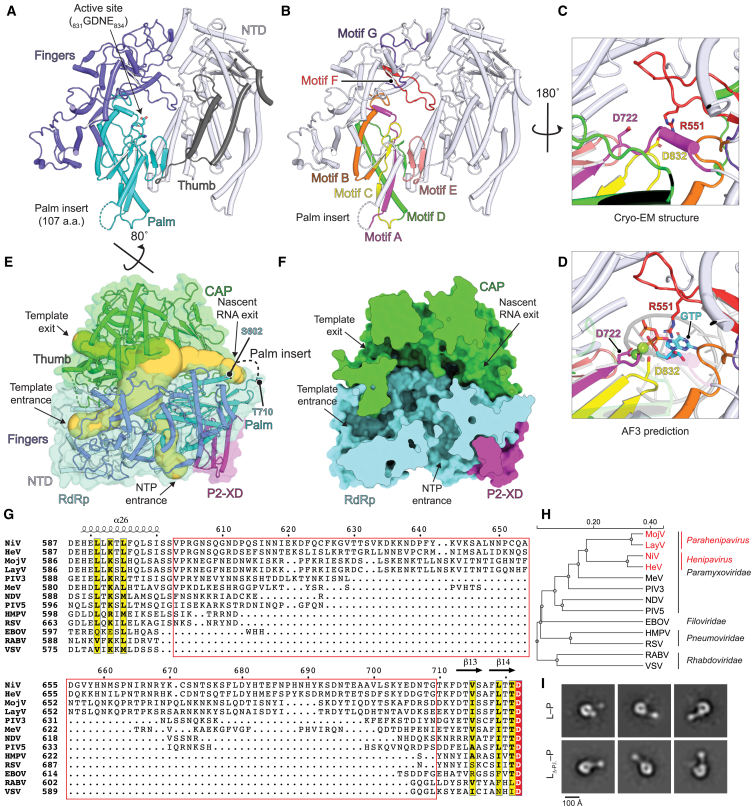
Molecular features of the NiV L polymerase core (A) NiV L RdRp domain. The fingers, palm, thumb, and NTD domains are shown. The palm active site GDNE motif residues are shown as sticks. The location of the palm insert is indicated. (B) The same view as in (A), with catalytic motifs A–G colored as indicated. (C) NiV L RdRp active site in the cryo-EM structure. Active site residues D832 (motif C), and nearby conserved residues D722 (motif A) and R551 (motif F) are shown as sticks. (D) AlphaFold 3 (AF3)^26^ prediction of the NiV L active site in the presence of a duplex RNA, GTP, and two magnesium ions (green spheres). D722 (motif A) and D832 (motif C) are predicted to coordinate metals, and R511 (motif F) is positioned to interact with the phosphate of the incoming nucleotide. Template and nascent RNA are shown in dark and light gray, respectively. Parts of motifs A and D are semi-transparent for clarity. See Figure S2D for predicted local distance difference test (pLDDT) scores. (E) NiV L-P complex shown as a ribbon diagram with transparent surface and channels shown as spheres (calculated by CAVER Web^28^). Entrance and exit channels are indicated. Residues S602 and T710, near the two ends of the palm insert, are near the nascent RNA exit channel. (F) Clipped surface of the NiV L-P complex showing the RNA synthesis channels in the same view as shown in (E). (G) Sequence alignment of the palm insert region of different nsNSV L proteins generated using ESPript 3.0.^29^ The NiV L palm insert (residues 603–709) is boxed in red. See Table S2 for virus name abbreviations and GenBank accession numbers. (H) Phylogenetic tree of the indicated nsNSVs based on L amino acid sequences generated using Clustal Omega.^30^ (I) Negative stain electron microscopy 2D classes of purified NiV L-P complexes for wild-type (WT) L or an L mutant in which much of the palm insert (residues 604–704) (L-_ΔP.I._) is deleted. P.I., palm insert. See also Figures S2K–S2M. See also Figure S2.

**Figure S2:**
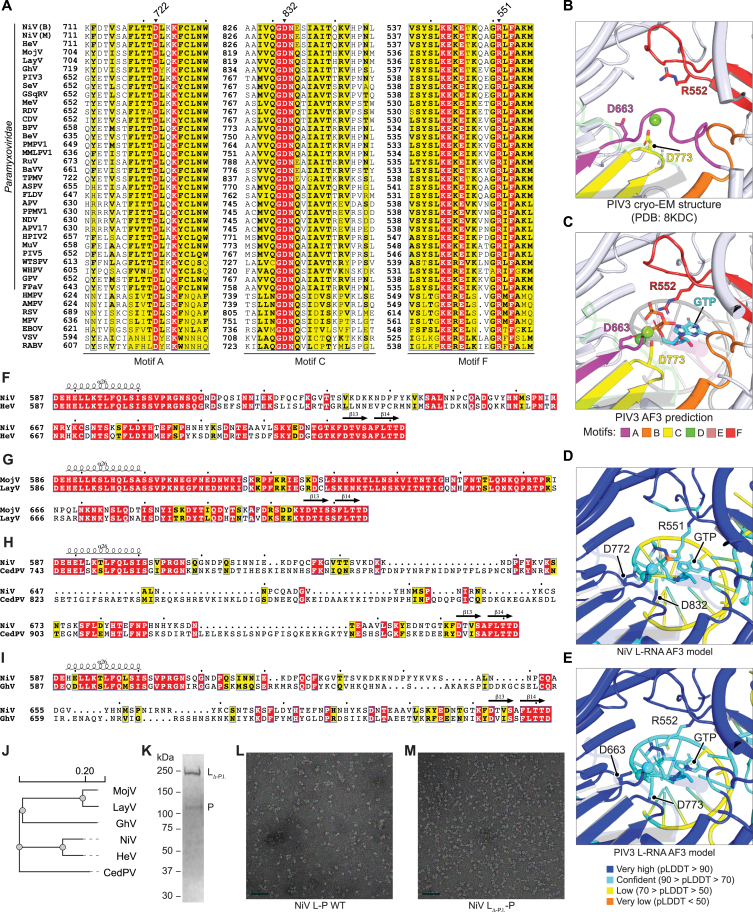
Partial sequence alignments of nsNSV L proteins, active site predictions, and negative stain images of NiV L, related to Figure 2 (A) Partial sequence alignment of nsNSV L proteins showing motifs A, C, and F. Motif C GDNE/Q is the catalytic site in the RdRp domain and is conserved among all the L proteins aligned. The fourth residue is glutamine in most sequenced viruses but is glutamate in a few viral sequences, including NiV L. Motif A D722, motif C D832, and motif F R551 are indicated by black triangles. See Table S2 for virus name abbreviations and GenBank accession numbers. (B) PIV3 L polymerase active site in the 2.7 Å cryo-EM structure of the PIV3 L-P complex.^27^ Active site residues D773 (motif C), D663 (motif A), and R552 (motif F) are shown as sticks. A magnesium ion (green sphere) was resolved in the cryo-EM structure interacting with D773 in the catalytic center. (C) AF3^26^ prediction of the PIV3 L active site in the presence of a duplex RNA, GTP, and two magnesium ions (green spheres). D663 (motif A) and D773 (motif C) are predicted to coordinate metals, and R552 (motif F) is positioned to interact with the phosphate of the incoming nucleotide. Template and nascent RNA are shown in dark and light gray, respectively. Parts of motifs A and D are shown as semi-transparent for clarity. (D and E) pLDDT of the active sites of the AF3 model of NiV L-RNA (D) and PIV3 L-RNA (E). Residues with very high confidence (pLDDT > 90) are shown in deep blue, confidence (90 > pLDDT > 70) in light blue, low confidence (70 > pLDDT > 50) in yellow, and very low confidence (pLDDT < 50) in orange. (F–I) Amino acid sequence alignment of palm insert regions of NiV and HeV (F), MojV and LayV (G), NiV and CedPV (H), and NiV and GhV (I). CedPV has a particularly long palm insert. (J) Phylogenetic tree of the indicated parahenipaviruses (MojV and LayV) and henipaviruses (GhV, NiV, HeV, and CedPV) based on L amino acid sequences generated using Clustal Omega.^30^ (K) SDS-PAGE gel of eluted fraction from Streptactin purification of NiV L_Δ-P.I._-P run under reducing conditions. Imaging was performed with a stain-free gel system. (L) Negative stain image of NiV L-P WT. After Streptactin purification, NiV L-P was diluted to 0.03 mg/mL for imaging by negative stain electron microscopy. The scale bar is 50 nm. (M) Negative stain image of NiV L_Δ-P.I._-P. After Streptactin purification, NiV L_Δ-P.I._-P was diluted to 0.06 mg/mL for negative stain electron microscopy. The scale bar is 50 nm.

The NiV L RdRp domain has a conventional right-hand “fingers-palm-thumb” organization containing seven motifs involved in catalysis (A–G), like other nsNSV RdRp domains (Figures 2A and 2B). Motifs A–E are in the palm while motifs F and G are in the fingers. Motif C contains a “GDNE” motif (residues 831–834), which is part of the active site (Figure S2A; Data S3). In the unliganded active site, protrusion of the β-hairpin loop in motif F in the fingers domain positions the side chain of R551 near (within 5 Å) motif C residue D832 (part of the GDNE motif; Figure 2C). R551 is universally conserved in nsNSV polymerases (Figure S2A; Data S3). Prediction of an RNA duplex- and nucleotide-bound NiV L with AlphaFold 3 (AF3)^26^ suggests the side chain of R551 interacts with the phosphates of the incoming nucleotide during RNA elongation, while the side chains of D832 (motif C) and D722 (motif A) coordinate active site metals (Figures 2D and S2D). Interestingly, in the cryo-EM structure of L-P complexes for parainfluenza virus type 3 (PIV3), visualized without RNA or incoming nucleotide, the analogous motif F arginine is also positioned toward the motif C catalytic aspartate and an associated active site magnesium (Figures S2B, S2C, and S2E),^27^ supporting AF3 predictions of the NiV L active site.

In the assembled NiV L-P complex, there are channels for NTP entry, template entry, template exit, and nascent RNA exit (Figures 2E and 2F). The template entrance channel, identified by analogy to the Ebola virus (EBOV) and RSV L protein-promoter RNA-bound structures,^18^^,^^31^ involves surfaces of the RdRp and CAP domains (Figure 2F). The putative template exit channel, identified by analogy with other viral RdRps, is within the CAP domain (Figure 2F).^8^ The putative nascent RNA exit channel is formed by the RdRp domain and the CAP domain on the opposite side of L relative to the template entrance channel.

A distinctive feature in the NiV RdRp domain, when compared with that of most other nsNSV polymerases, lies in the region found between NiV L residues 600–713, which is located immediately N-terminal to motif A in the palm domain (Figures 2A and 2B). We did not observe cryo-EM density for residues 603–709 within this loop. Primary sequence alignments (Figure 2G; Data S3)^32^^,^^33^ show that this region of henipavirus and parahenipavirus L proteins has additional sequence, ranging from ∼100 to > 200 amino acid residues in length, compared with other nsNSVs, including most other paramyxoviruses. We refer to this additional sequence as the palm insert. Other than the large size and general feature of being rich in lysine and asparagine residues, there is substantial divergence in the henipavirus and parahenipavirus palm insert sequences, although it is mostly conserved among closely related viruses when they are examined as pairs (Figures 2H and S2F–S2J).

The boundary residues that we could observe for the palm insert, S602 and T710, are relatively close to each other in the structure (only ∼10 Å apart) and are located near the putative nascent RNA exit channel (Figure 2E). The proximity of the N- and C-terminal regions of the loop and disorder in cryo-EM maps suggests that the insert may form an appendage that does not contribute to the fold of the RdRp domain. To test this hypothesis, we generated NiV L that lacks residues 604–704 (NiV L_Δ-P.I_.) and co-expressed it with P in insect cells. Purified particles examined by negative stain electron microscopy had a similar shape to particles for the wild-type (WT) NiV L-P complex (Figures 2I and S2K–S2M). These findings confirmed that the palm insert does not contribute to the fold of the RdRp domain.

### The CAP domain

**Figure 3:**
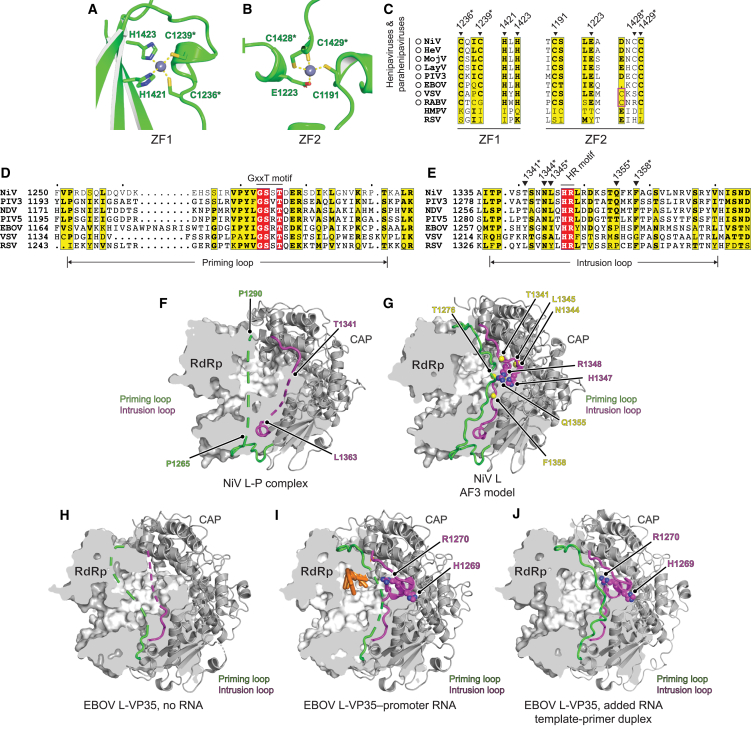
Features of the NiV L CAP domain (A and B) CAP domain ZF1 (A) and ZF2 (B). Zinc ions are shown as gray spheres. Asterisks indicate residues selected for mutational analysis. (C) Sequence alignment of examples of nsNSV L CAP ZFs, which are present in the L proteins of the viruses indicated by the circles, generated using ESPript 3.0.^29^ Numbering is based on NiV L. Triangles indicate residues that form ZFs. Residues within the purple box are alternative residues that form ZFs in VSV and RABV. Asterisks indicate residues selected for mutational analysis. (D and E) Sequence alignment of the priming loop (D) or intrusion loop (E) of NiV L and the indicated nsNSV L proteins. Triangles indicate positions in the intrusion loop that were selected for mutagenesis in addition to the HR motif. (F) The NiV L RdRp domain is shown in surface representation with a partially clipped surface, and the CAP domain is shown as a ribbon diagram. The disordered portions of the priming and intrusion loops are shown as dashed lines. Boundary residues are indicated. (G) AF3^26^ prediction of the NiV L RdRp and CAP domains generated in the absence of nucleic acids. Intrusion loop HR residues (H1347 and R1348) are indicated. Yellow spheres indicate residues selected for mutational analysis. (H–J) Cryo-EM structures of EBOV L-VP35 without RNA (PDB: 7YER)^36^ (H), with promoter RNA (PDB: 8JSL)^31^ (I), and with the addition of an RNA template-primer duplex but no RNA density observed in maps (PDB: 7YES)^36^ (J). Nucleic acids are shown as orange sticks. Intrusion loop HR residues are indicated. See also Figure S3.

**Figure S3:**
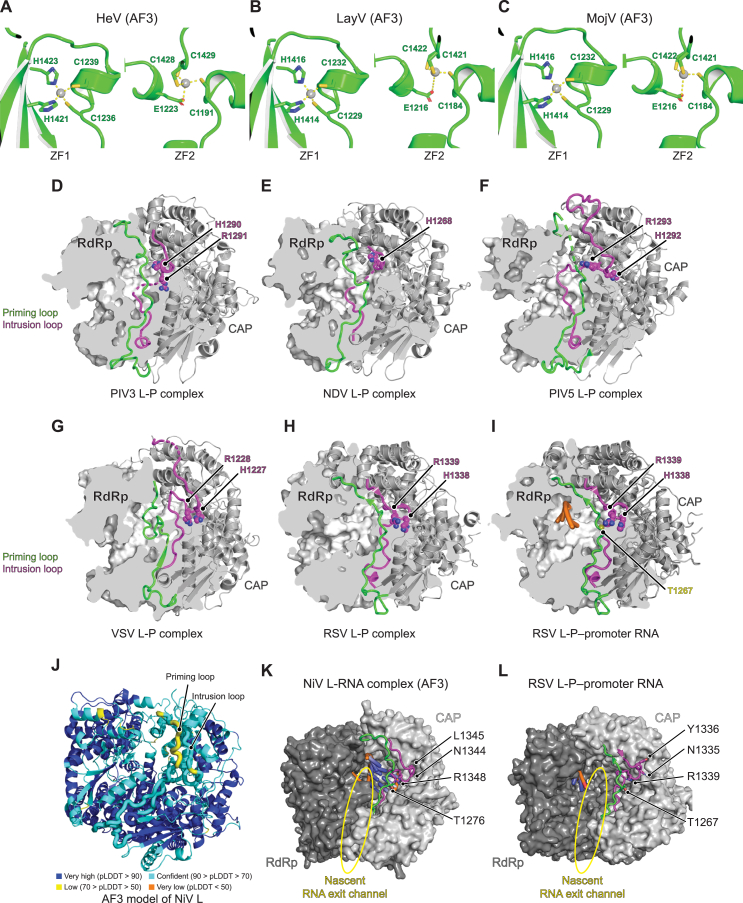
CAP domain features for additional polymerase structures, related to Figure 3 (A–C) AF3^26^ predicted ZFs of HeV (A), LayV (B), and MojV (C). Zinc ions are shown as gray spheres, and residues coordinating zinc ions are shown as sticks. (D–I) Cryo-EM structures of PIV3 L-P (PDB: 8KDC)^27^ (D), NDV L-P (PDB: 7YOU)^41^ (E), PIV5 L-P (PDB: 6V85)^37^ (F), VSV L-P (PDB: 6U1X)^39^ (G), RSV L-P (6PZK)^20^ (H), and RSV L-P-RNA (PDB: 8SNX)^18^ (I). The L protein RdRp domains are shown in surface representation with partially clipped surfaces, and the CAP domains are shown as ribbon diagrams. The priming (green) and intrusion (purple) loops are shown. The disordered portions within these loops that were not resolved in the cryo-EM structures are shown as dashed lines. HR residues are indicated. Except for NDV, whose intrusion loop HR motif arginine (R1269) was in a disordered segment, the HR residues could be resolved. Nucleic acids are shown in orange. (J) pLDDT scores of an AF3^26^ model of the NiV L RdRp and CAP domains in the absence of RNA. The priming and intrusion loops are indicated and shown with thicker ribbon representation. (K) AF3 model of RNA-bound NiV L showing the location of the priming (green) and intrusion (purple) loops with respect to the putative nascent RNA channel, the general location of which is indicated by the yellow oval. Intrusion loop residues N1344, L1345, and R1348, which are indicated, were subjected to mutational analysis using minigenome assays (see Figure S6R). Priming loop residue T1276 is equivalent to RSV priming loop residue T1267, which is a part of the GxxT motif. (L) Cryo-EM structure of RSV L-P bound to promoter RNA (PDB: 8SNX)^18^ showing the location of the priming (green) and intrusion (purple) loops with respect to the putative nascent RNA channel, the general location of which is indicated by the yellow oval. RSV L intrusion loop residues N1335, Y1336, and R1339 are equivalent to NiV L residues N1344, L1345, and R1348. RSV L priming loop residue T1267 is equivalent to NiV L residue T1276.

The CAP domain of nsNSV polymerases plays roles in RNA synthesis initiation and elongation, in addition to catalyzing the polyribonucleotidyltransferase (PRNTase) step of cap addition.^7^^,^^8^^,^^12^^,^^34^ For vesicular stomatitis virus (VSV), the CAP domain also interacts with the template RNP.^35^ Features that have been identified in other nsNSV CAP domains include zinc-finger (ZF) motifs, a priming loop, and an intrusion loop. The ZF motifs are present in the L proteins of many but not all nsNSVs (Figures 3A–3C and S3A–S3C; Data S3),^27^^,^^31^^,^^36^^,^^37^^,^^38^^,^^39^^,^^40^^,^^41^^,^^42^ but their function is not known. The priming loop is thought to be involved in stabilizing the RNA synthesis initiation complex, and the intrusion loop contains the catalytic histidine-arginine (HR) motif required for PRNTase activity (Figures 3D and 3E).^7^^,^^8^^,^^12^ The priming and intrusion loops have been captured in multiple distinct conformations for different nsNSV polymerases, each thought to represent a different functional state (Figures S3D–S3I). In the cryo-EM map of the NiV L-P complex, most of the priming loop and intrusion loop are not visible, likely due to flexibility (Figure 3F), although both loops could be predicted using AF3 (Figures 3G and S3J). The state captured with NiV L-P is most similar to EBOV L-VP35 complexes visualized in the absence of RNA, in which most of both loops were disordered, with the loops becoming partially ordered in the presence of promoter RNA and fully ordered in the presence of an RNA template-primer duplex (Figures 3H–3J).^31^^,^^36^

### Structure of NiV P

**Figure 4:**
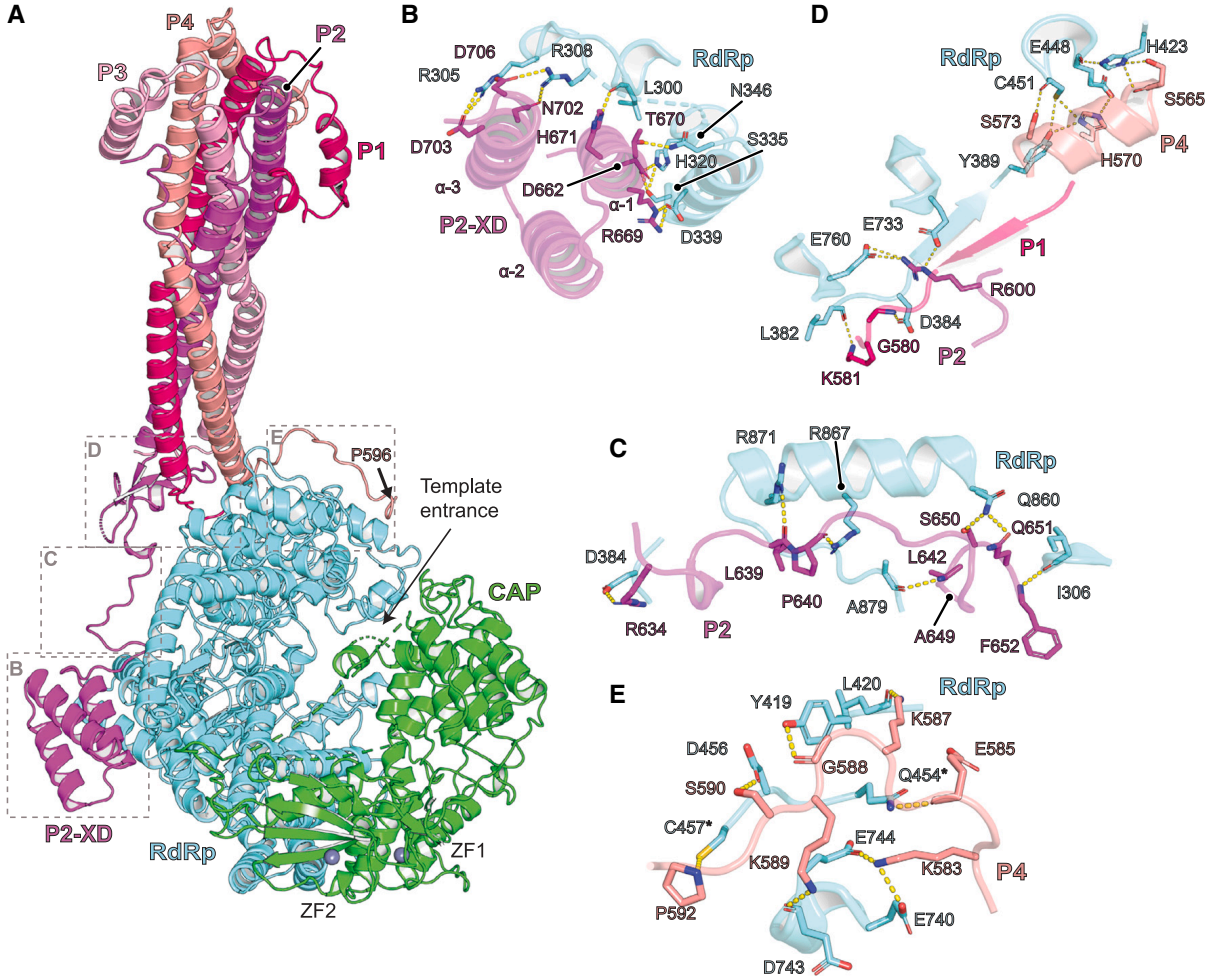
Interactions between NiV P and L RdRp (A) Ribbon diagram of the NiV L-P complex. Interactions between L and tetrameric P occur over four main regions, which are indicated by dashed boxes with detailed interactions provided in (B)–(E). (B) Interactions between the P2-XD ⍺-helices and the L RdRp domain. (C) Interactions between the P2 linker, which connects the P2 OD with the P2-XD, and the L RdRp domain. (D) Interactions between the P1, P2, and P4 and the L RdRp domain. (E) Interaction between the P4 peptide extension and the L RdRp domain. L residues mutated in functional assays (Q454 and C457) are indicated with an asterisk. See also Figure S4.

In the NiV L-P complex, most of the N-terminal region of P remains disordered, as is the case for other paramyxoviruses^27^^,^^37^^,^^41^^,^^42^ (Figure 1A). The C-terminal regions of P fold onto L by forming several extended arms, with one of the protomers (designated P2 here) providing contacts through the XD domain, which forms a bundle of three ⍺-helices (Figures 4A and 4B). P2-XD helices ⍺-1 and ⍺-3 interact with L (Figure 4B). P buries ∼3,478 Å^2^ of surface area on NiV L. The P2 polypeptide chain makes polar contacts as it courses toward the XD domain (Figure 4C), and the extended arms of P1 and P4 form an extensive network of polar interactions with the RdRp domain (Figures 4D and 4E). As part of this interaction network, P1 interacts with the RdRp domain through β-strand augmentation (Figure 4D). An extension in P4 interacts with the RdRp domain on the opposite face that binds P2 XD, and P4 residues K583, K587, and K589 make interactions that anchor a loop onto the RdRp domain (Figure 4E).

### Dynamics of L-associated phosphoprotein

**Figure S4:**
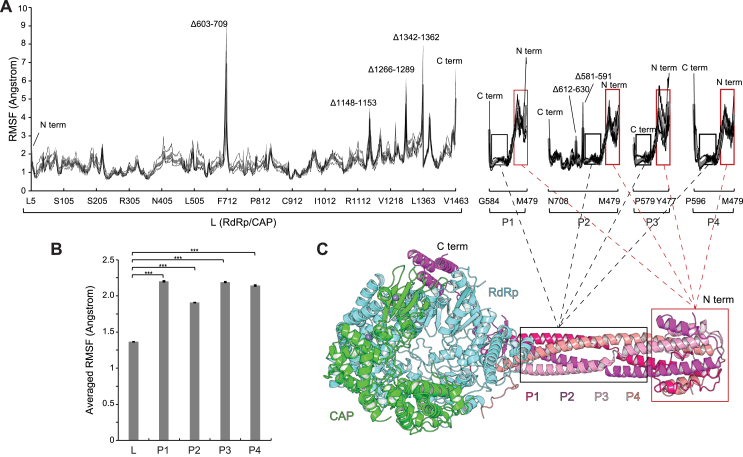
Tetrameric P is flexible when assembled onto the L polymerase core, related to Figure 4 (A) Per-residue root-mean-square-fluctuation (RMSF) from 100 ns molecular dynamics (MD) simulations of the protein complex. Three thin lines represent individual MD runs. A thick line represents averaged RMSF values. For the RdRp/CAP portion of the plot, regions denoted as “Δ” are stretches of residues that had no observable or interpretable density in cryo-EM maps and therefore were not modeled in atomic coordinates, and high RMSF fluctuations in these regions are likely due to interruption of the polypeptide chain. (B) Mean RMSF values calculated during MD simulations for L (RdRp/CAP), P1, P2, P3, and P4 are plotted. The comparison of mean RMSF values between two groups was performed using an unpaired, two-tailed Student’s t test. Error bars represent standard errors. ^∗∗∗^*p* < 0.001. (C) Cryo-EM structure of the NiV L-P complex provided as a reference for regions of P with increased flexibility.

We could only resolve 239 residues of P, approximately one-third of the molecule, as is the case in other structures of L-P complexes. The NiV polymerase structure contains the longest P segment visualized in paramyxovirus L-P complexes, particularly when the folded-back helices of the ⍺-helical cap structure are considered, as the two bent helices add an additional ∼50 Å to the 100 Å coiled core (Data S2). We hypothesized that the network of polar interactions P makes with L would likely be malleable, allowing the pose of the P OD stem to vary with respect to the polymerase core. Consistent with such motion, the distal (N-terminal tip) of P had the lowest local resolution, with smeared-out features of the ⍺-helical cap structure map region (Figures 1E and S1E). Three-dimensional (3D) variability analysis^43^ of the cryo-EM dataset revealed a swiveling motion of the P OD with respect to the polymerase core (Video S1). We also performed 100 ns molecular dynamics simulations using the cryo-EM structure as a starting point (Figure S4; Video S2; Data S4). Root-mean-square fluctuations (RMSF) were much higher for each of the P protomers than they were for the polymerase core (RdRp/CAP), with the highest fluctuations observed in the N-terminal portions of the ⍺-helical cap structure.

Video S1. 3D variability analysis of NiV L-P complexes, related to Figure 1 We performed 3D variability analysis on the cryo-EM data set of NiV L-P complexes using cryoSPARC.^43^^,^^59^ The movie shown is from a volume series recorded in UCSF Chimera.^52^ See methods for additional details.

Video S2. MD simulation of the NiV L-P complex in two views, related to Figures 1 and S4 We performed 100 ns MD simulations of the NiV L-P cryo-EM structure. The simulation is shown from both faces of the complex. L domains and P protomers are colored as shown in Figure S4C.

### Basis for NiV resistance to broad-spectrum inhibitor

**Figure S5:**
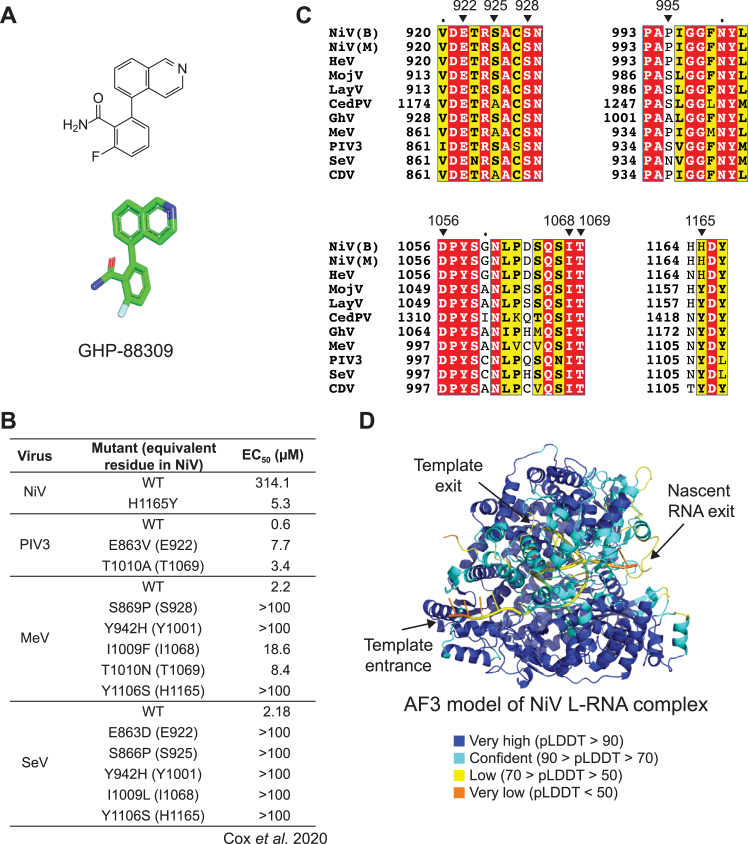
GHP-88309 structure, previously described activity profile, and conservation of inhibitor binding site, related to Figure 5 (A) Structure of the broad-spectrum paramyxovirus antiviral GHP-88309 in 2D (top) and 3D (bottom) representations. (B) Summary of results of antiviral activity assays for GHP-88309 as determined by Cox et al. using minigenome assays for NiV, PIV3, MeV, or recombinant SeV.^44^ (C) Partial sequence alignment of the L proteins of the indicated viruses. Residues implicated in inhibitor resistance by Cox et al.^44^ or near the drug in docking experiments are indicated. (D) pLDDT of AF3^26^ model of NiV L-RNA complex.

**Figure 5:**
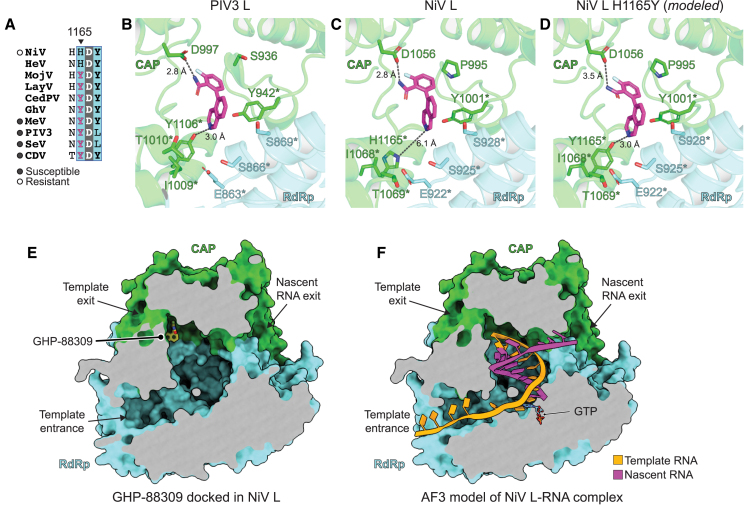
Basis for resistance of NiV L to a broad-spectrum paramyxovirus inhibitor (A) Sequence alignment of L protein residues 1164 to 1167 in the CAP domain for the indicated viruses. The broad-spectrum inhibitor GHP-88309 is active against the viruses indicated by the filled circles.^44^ (B–D) Docking of GHP-88309 into the predicted binding site in PIV3 L (B), NiV L (C), or NiV L H1165Y mutant (modeled *in silico*) (D). Predicted interactions or distances between the ring nitrogen of the inhibitor and contacting amino acids are shown as black dashed lines. Residues with asterisks are sites of L substitutions that affect inhibitor susceptibility (see Figures S5B and S5C). (E) Clipping surface showing the position of the GHP-88309 docked in NiV L. The RdRp and CAP domains are shown as surfaces and clipped to reveal template entry and exit channels. (F) Clipping surface showing the position of modeled template and nascent RNA in an AF3^26^ prediction of NiV L bound to RNA and nucleotide. An incoming GTP molecule shown as sticks for orientation, but note that the clipping surface does not include the NTP entrance channel, which is out of the plane of view. Active site metals are not shown for clarity. See Figure S5D for pLDDT scores. See also Figure S5.

GHP-88309 is a broad-spectrum, non-nucleoside inhibitor of paramyxovirus polymerases with activity against PIV3, measles virus (MeV), and SeV.^44^^,^^45^^,^^46^ While GHP-88309 is active against many paramyxoviruses, it is not active against NiV, with a half-maximal effective concentration (EC_50_) of 314 μM in a minigenome assay (Figures S5A and S5B).^44^ Photoaffinity labeling experiments, resistance mapping, and *in silico* docking experiments revealed that GHP-88309 binds in a cavity at the intersection of the RdRp and CAP domains in MeV L.^44^^,^^46^ A histidine in the predicted drug binding site (H1165) in NiV L has been implicated in resistance,^44^ and it was shown that introducing the H1165Y substitution into NiV L, which mimics the residue that is naturally present in susceptible polymerases (Figure 5A), renders NiV L susceptible to GHP-88309 inhibition, with the EC_50_ value decreasing by sixty-fold (5.3 μM) (Figure S5B).^44^

To better understand the basis for drug susceptibility conferred by the H1165Y substitution, we performed *in silico* docking experiments with GHP-88309. Given that PIV3 L is susceptible, we used the PIV3 L cryo-EM structure^27^ as a starting point for docking experiments. Docking of GHP-88309 with PIV3 L revealed a low-energy binding pose that placed GHP-88309 at the expected site at an interface between the CAP and RdRp domains, with the isoquinoline ring interacting with Y942 (PIV3 L numbering) and the drug surrounded by residues implicated in drug resistance (Figure 5B). The pose is highly akin to that predicted by Cox et al., who used the parainfluenza virus type 5 (PIV5) structure as a starting point to generate a homology model of MeV L.^44^

We next performed docking experiments with the NiV L cryo-EM structure in its WT form or containing the H1165Y substitution introduced *in silico* (Figures 5C and 5D). A low-energy pose was identified in NiV L at a binding site that is like that of PIV3. Comparison of the PIV3, NiV L, and NiV L H1165Y docking models suggests that NiV L is resistant to the inhibitor because H1165 does not make a polar contact that Y1165 can make with the ring nitrogen of GHP-88309 (Figures 5B–5D). Given that residues near the drug binding site are otherwise generally conserved (Figure S5C), GHP-88309 could serve as a starting point to identify modified inhibitors that target this site in the NiV L.

GHP-88309 was reported to inhibit *de novo* initiation of RNA synthesis at the promoter.^44^ However, inspection of the data presented indicates inhibition becomes more pronounced as the product length is increased, indicating that it inhibits a step after initiation of RNA synthesis.^44^ Consistent with this finding, the position of GHP-88309 in our docking experiment (Figure 5E) in comparison to AF3-modeling of RNA-bound NiV L (Figure 5F) suggests that the inhibitor would sterically block exit of the template from the polymerase, which would be expected to inhibit polymerase progression and thus efficient elongation of the RNA product.

### Functional analysis of the HR motif, P4 extension, palm insert, and ZFs

The transcription and genome replication processes performed by the NiV polymerase each involve a different sequence of events (reviewed in Kleiner and Fearns).^17^ It is thought that during transcription, the polymerase starts from position 1U of the leader (le) promoter, synthesizes a small RNA that is released, and then re-initiates RNA synthesis at a gene start (gs) signal.^17^ Having initiated RNA synthesis at the gs signal, the polymerase co-transcriptionally caps the pre-mRNA, methylates the cap, and then elongates the pre-mRNA until it reaches a gene end (ge) sequence, where it is directed to polyadenylate and then release the mature mRNA.^6^ Following mRNA release, the polymerase can then locate the gs signal for the next gene. By continuing this sequence of events, the polymerase produces a series of subgenomic mRNAs, each with a 5′ methylguanosine cap and 3′ polyadenylate tail.^17^ During replication, the polymerase again initiates at position 1U of the le promoter but elongates beyond the end of the le region, fails to recognize the gene junction signals, and produces a full-length, uncapped, non-polyadenylated antigenome, which is concurrently encapsidated by the N protein. The encapsidated antigenome in turn serves as a template for the synthesis of encapsidated genome RNA.^17^ We sought to determine if key features identified in the cryo-EM structure of the NiV L-P complex are required for transcription and/or RNA replication.

**Figure 6:**
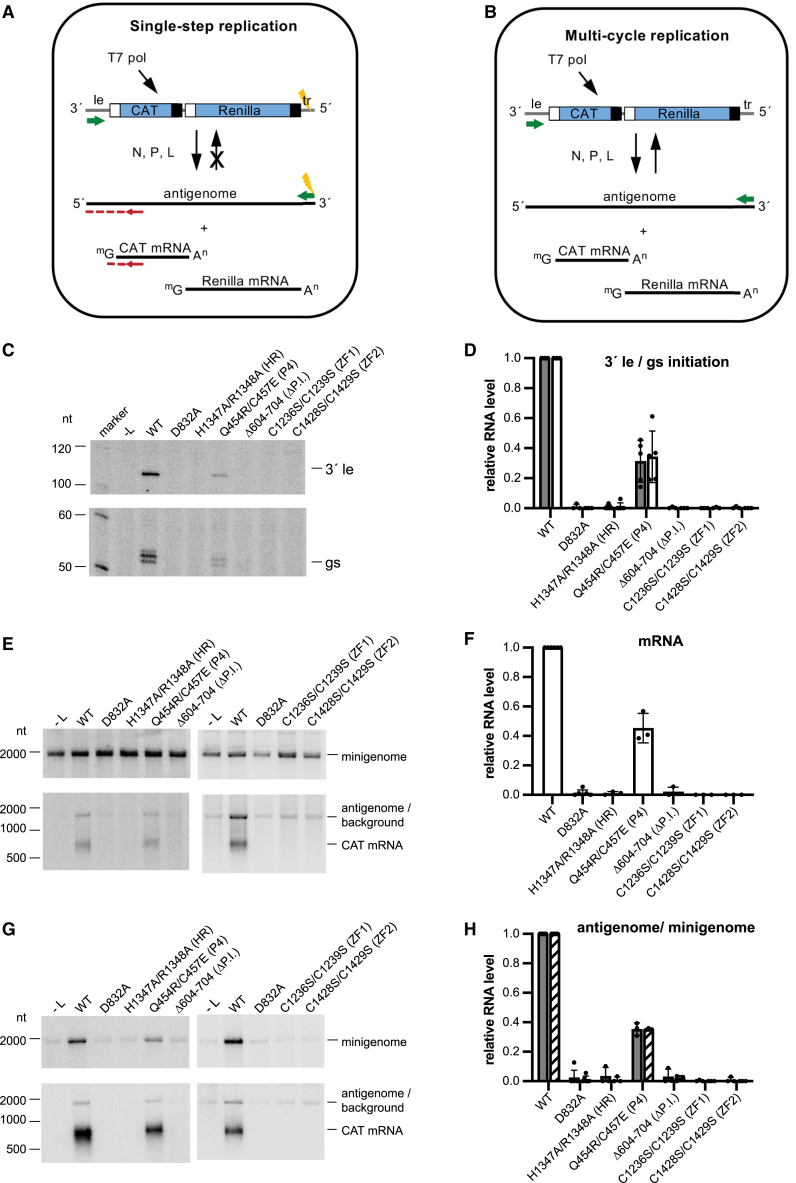
Functional analysis of the L HR motif, P4 extension, palm insert, and zinc-finger motifs using a minigenome system (A and B) Diagrams illustrating the structure of the minigenomes and the products that are generated during RNA replication and mRNA transcription. White and black boxes indicate gene start and gene end signals, respectively. Green arrows indicate promoters. (A) shows a minigenome limited to a single-step of RNA replication by a mutation in the 5′ trailer (tr) region (indicated with a lightning symbol). This mutation prevents the antigenome that is synthesized by the NiV polymerase from acting as a template for further rounds of minigenome synthesis. The red arrow illustrates the positioning of the primer used for primer extension analysis, and the red dotted line illustrates the cDNA that would be generated by primer extension. (B) shows a minigenome with an intact tr region that serves as a template for multiple cycles of RNA replication, resulting in amplified levels of encapsidated minigenome RNA. (C) Primer extension analysis of positive-sense RNA generated by the NiV polymerase in the single-step minigenome system, using the primer indicated in (A). The upper and lower panels show a phosphorimage scan of the same gel, with the intervening region excised. Note that the cDNA bands for the gs-initiated products sometimes migrated as doublet or triplet bands. This could be a consequence of the reverse transcriptase encountering the methylated cap on the mRNA. (D) Quantification of the levels of 3′ le (gray bars) and gs (white bars) initiation products from replicates of the experiment shown in (C). The bars show the mean and standard deviation from three independent experiments for each mutant, except for the H1347A/R1348A (HR) mutant (*n =* 6) and the Q454R/C457E (P4) mutant (*n =* 5). (E) Northern blot analysis of RNAs generated in the single-step minigenome system. The upper panel shows negative-sense minigenome template RNA generated by T7 RNA polymerase. The lower panel shows the corresponding positive-sense antigenome RNA and CAT mRNA generated by the NiV polymerase. Note that the antigenome RNA co-migrates with a background band. (F) Quantification of levels of mRNA from replicates of the northern blots shown in (E). The bars show the mean and standard deviation (SD) from three independent experiments for each mutant. (G) Northern blot analysis of RNAs generated in the multi-cycle minigenome system shown in (B). The upper panel shows negative-sense minigenome template RNA generated by T7 RNA polymerase and amplified by NiV polymerase. The lower panel shows the corresponding positive-sense antigenome RNA and CAT mRNA generated by the NiV polymerase. (H) Quantification of levels of antigenome (gray bars) and minigenome (hatched bars) from replicates of the northern blots shown in (G). The bars show the mean and SD from three independent experiments for each mutant. See also Figure S6

**Figure S6:**
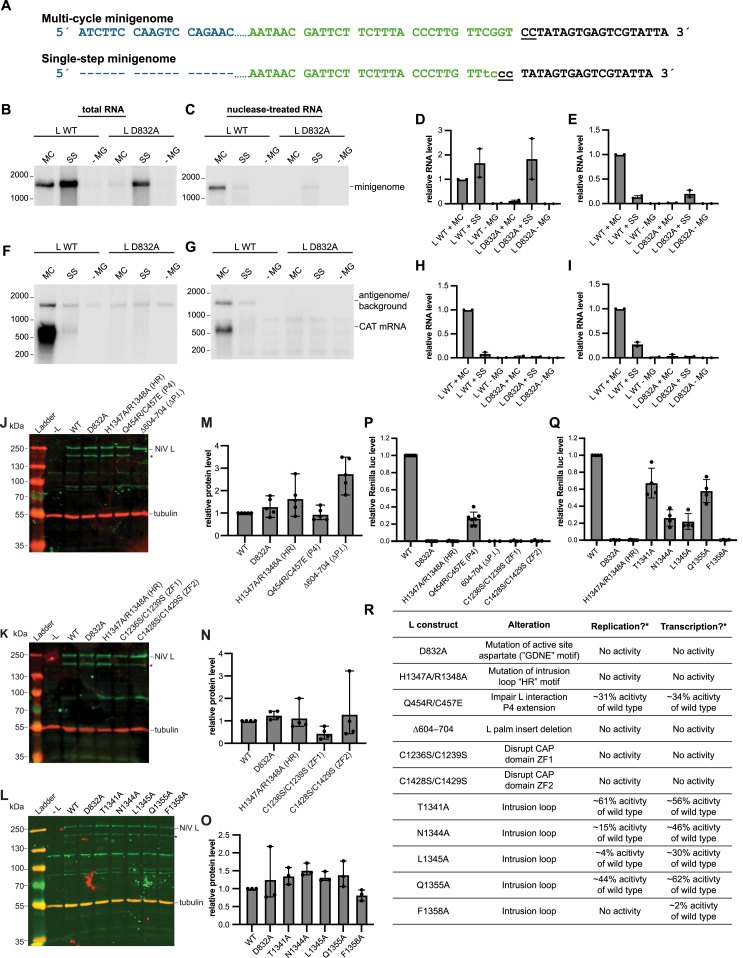
Validation of the single-step minigenome assay, mutant L expression, gene expression activities of L mutants measured by luciferase activity, and summary of functional studies, related to Figures 6 and 7 (A) Sequence alignment of the 3′ ends of the antigenome RNAs of the multi-cycle and single-step minigenomes (Figures 6A and 6B). The alignment shows the internal NiV promoter element (promoter element 2; blue type), the complement of the trailer region (green type), and the T7 promoter (black type). The T7 RNA polymerase initiates opposite the C residues that are underlined to synthesize negative-sense RNA. Dashes indicate residues that were deleted in the single-step minigenome. Substitutions introduced into the trailer promoter of the single-step minigenome are shown in lowercase type and were designed to ablate the promoter and enhance T7 promoter activity. The multi-cycle minigenome followed the rule of six (i.e., its nucleotide length was divisible by six) except for the additional G residues contributed by the T7 promoter, and the single-step minigenome followed the rule of six, including the additional G residues contributed by the T7 promoter. (B and C) Northern blot analysis of negative-sense (minigenome sense) RNA generated by either T7 RNA polymerase and NiV polymerase in the case of the multi-cycle minigenome or by T7 RNA polymerase alone in the case of the single-step minigenome. The blots show RNA generated in cells transfected with plasmids expressing either WT L or L with a substitution in the GDNE motif of the RdRp (L D832A). Cells were transfected with plasmids expressing either multi-cycle (MC) or single-step (SS) replication minigenomes, or minigenome (MG) was omitted from the transfection (−MG). (B) shows total RNA harvested from transfected cells. (C) shows RNA from a parallel transfection in which the cell lysate was treated with micrococcal nuclease prior to RNA purification to reduce levels of unencapsidated RNA and distinguish encapsidated minigenome template RNA. (D and E) Quantification of the bands from replicates of the experiments shown in (B) and (C), respectively. (F and G) RNA from the same transfections as used for (B) and (C), in which the northern blot was probed to detect positive-sense RNA (antigenome and CAT mRNA). (H and I) Quantification of the bands from replicates of the experiments shown in (F) and (G), respectively. (H) shows quantification of CAT mRNA, and (I) shows quantification of nuclease-resistant antigenome RNA. The data shown in (B), (C), (F), and (G) are representative of two independent experiments. The bars in (D), (E), (H), and (I) show the two data points and the mean for the two independent experiments, with normalization to L WT + MC minigenome in each case. This experiment shows that the single-step replication minigenome had a stronger T7 promoter than the multi-cycle replication minigenome (B and D). However, the single-step replication minigenome was not amplified by WT L protein (C and E) and consequently produced relatively low levels of CAT mRNA (F and H) and antigenome (G and I). (J–L) Western blot analysis of minigenome-transfected cell lysates probed with a strep-tag-specific antibody to detect L (green), and tubulin (red) was detected with a specific antibody as a loading control. The band marked with an asterisk is the appropriate molecular weight to be truncated L protein that had been cleaved at the palm insert. (M–O) Quantification of the full-length NiV L band from replicates of the experiments shown in (J)–(L), respectively. Each bar represents the mean and SD derived from three independent experiments. (P and Q) Renilla luciferase activity of L mutants in assays with a single-step minigenome. The bars show the mean and SD from three independent experiments, except for the H1347A/R1348A (HR) and Q454R/C457E (P4) mutants (*n =* 7). (R) Summary of functional studies with NiV minigenome system. ^∗^Activity levels determined by primer extension analysis of RNA initiated at the 3′ end of the le region (replication) or gs signal (transcription).

We examined NiV L mutants using a cell-based minigenome system, in which NiV minigenome and NiV N, P, and L protein expression are driven by intracellular expression of T7 RNA polymerase (Figures 6A and 6B).^10^ The advantage of this system, compared with *in vitro* biochemical assays, is that it reconstitutes each of the different steps involved in transcription and RNA replication in the order in which they would naturally occur in a cellular environment. We used a dicistronic minigenome in which the first gene contains *chloramphenicol acetyltransferase* (CAT) reporter gene sequence, and the second gene contains a *Renilla luciferase* reporter gene. We used a single-step minigenome that was limited to the antigenome synthesis step of RNA replication due to a mutation in the trailer (tr) region that ablated the promoter at the 3′ end of the antigenome (Figures 6A and S6A–S6I). This single-step minigenome assay allows the effects of polymerase mutations on transcription to be assessed independently of any effect that the mutations have on RNA replication and vice versa (Figure 6A). For all assays, the WT and mutant L proteins contained a C-terminal strep tag to allow monitoring of L protein expression. Comparison of untagged and strep-tagged WT L in a minigenome luciferase assay showed that the tag had minimal effect on polymerase activity, with luciferase expression from strep-tagged L being at 85% of untagged L levels (data not shown). As a negative control, we generated an L plasmid that contains a D832A substitution in the RdRp active site GDNE motif (Figures 2C and 2D) and should yield an inactive polymerase.

Mutations were introduced into L to substitute the HR motif in the intrusion loop of the CAP domain (H1347A/R1348A) (Figures 3E and 3G), disrupt the interface with the P4 extension that contacts the RdRp domain (Q454R/C457E) (Figure 4E), delete the palm insert in the RdRp domain (Δ604–704) (Figure 2G), and individually disrupt ZF1 (C1236S/C1239S) or ZF2 (C1428S/C1429S) that are in the CAP domain (Figures 3A–3C). Western blot analysis confirmed that all mutant L proteins were efficiently expressed, although typically a cleavage product of ∼195 kDa was also detected (Figures S6J, S6K, S6M, and S6N). This product is the appropriate size to represent the C-terminal portion of L following cleavage at the palm insert region, and consistent with this, the Δ604–704 mutant did not show evidence of this cleavage product (Figure S6J). Both ZF mutants were also consistently less prone to this cleavage (Figure S6K). The ZF1 mutant (C1236S/C1239S) was consistently expressed at a lower level than WT L and the other mutants (∼45% of WT levels; Figures S6K and S6N), suggesting that this ZF helps to maintain structural integrity of the L protein. Nonetheless, sufficient levels of the ZF1 mutant L protein were expressed for functional analysis.

RNA products generated by the L protein mutants were examined by primer extension analysis performed using a primer corresponding to sequence downstream of the first gs signal (Figure 6A). This primer would be able to detect antigenome RNA that had been elongated beyond the le region into the first gene, and mRNA initiated at the first gs signal. Analysis of RNA initiated at the 3′ end of the le region (antigenome) and gs signal (mRNA) showed that the L H1347A/R1348A, L Δ604-704, and two ZF mutants generated no detectable RNAs, whereas the L Q454R/C457E mutant initiated antigenome and mRNA at ∼31% and 34% of WT levels, respectively (Figures 6C and 6D). This decrease in transcription was also shown by northern blot analysis of CAT mRNA (Figures 6E and 6F) and measurement of luciferase activity (Figure S6P). Unfortunately, levels of antigenome RNA generated from a single-step minigenome could not be measured reliably by northern blotting due to a co-migrating background band on some blots. While the primer extension analysis shown in Figures 6C and 6D measures the products of the early steps of replication, it does not measure full-length replication products. To assess the effects of the mutations on full-length replicative RNAs, we also examined the polymerase mutants using a multi-cycle minigenome that had an intact tr region so that the polymerase could perform multiple cycles of RNA replication (Figure 6B). This analysis confirmed that the L H1347A/R1348A, L Δ604–704, and ZF mutants were completely defective in RNA replication and that the L Q454R/C457E mutant was only partially active (Figures 6G and 6H). Thus, each of the mutations inhibited transcription and RNA replication, indicating that the L protein features that were mutated are fundamental to polymerase activity.

### Functional analysis of the intrusion loop

By analogy with rhabdoviruses, the HR motif is required for the PRNTase activity of mRNA cap addition.^47^^,^^48^^,^^49^ Thus, it was not surprising that substitutions in the HR motif inhibited mRNA synthesis (Figures 6C–6F). However, the L H1347A/R1348A mutant was also completely defective in RNA replication (Figures 6C, 6D, 6G, and 6H). This result indicates that the L H1347A/R1348A mutation either affects polymerase structure or inhibits a core activity of the polymerase that is required for both transcription and replication (in addition to inhibiting capping), or the H1347A/R1348A mutation has another distinct effect on RNA replication in addition to inhibiting capping. The HR motif lies in the intrusion loop (Figures 3E–3G), which does not have a well-defined role. To gain further insight into intrusion loop function, we generated five additional L mutants. Each of these residues was individually substituted with alanine, and the mutant L proteins were tested in the same set of assays as described above.

**Figure 7:**
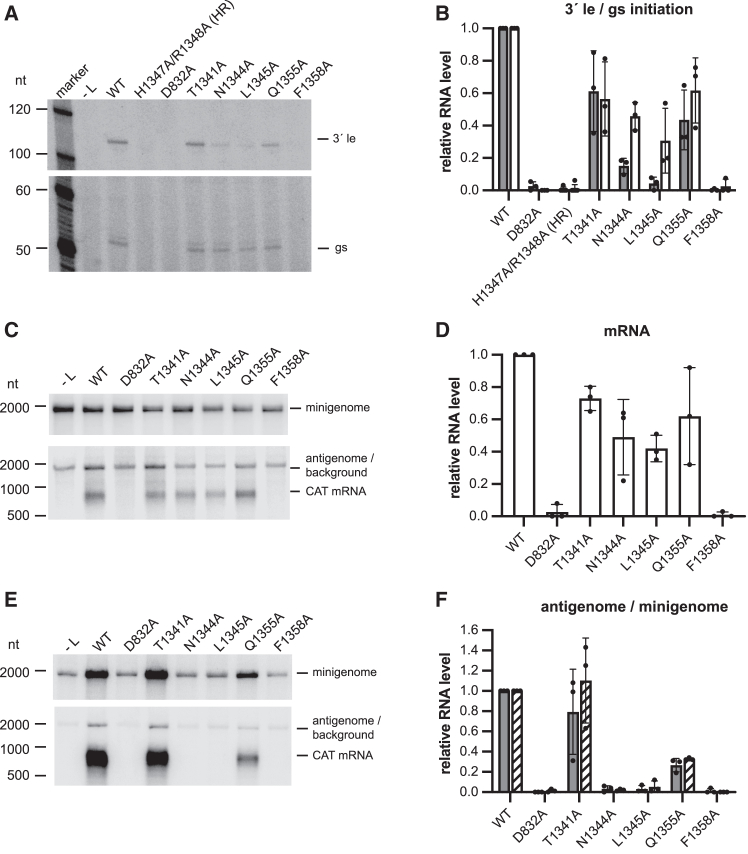
Functional analysis of the intrusion loop using a NiV minigenome system (A) Primer extension analysis of positive-sense RNA generated by the NiV polymerase in the single-step minigenome system, using the primer indicated in Figure 6A. The upper and lower panels show a phosphorimage scan of the same gel, with the intervening region excised. (B) Quantification of the levels of 3′ le (gray bars) and gs (white bars) initiation products from replicates of the experiment shown in (A). The bars show the mean and SD from three independent experiments for each mutant, except for the H1347A/R1348A (HR) mutant (*n =* 6). (C) Northern blot analysis of RNAs generated in the single-step minigenome system. The upper panel shows negative-sense minigenome template RNA generated by T7 RNA polymerase. The lower panel shows the corresponding positive-sense antigenome RNA and CAT mRNA generated by the NiV polymerase. Note that the antigenome RNA co-migrates with a background band. (D) Quantification of levels of mRNA from replicates of the northern blots shown in (C). The bars show the mean and SD from three independent experiments for each mutant. (E) Northern blot analysis of RNAs generated in the multi-step minigenome system. The upper panel shows negative-sense minigenome template RNA generated by T7 RNA polymerase and amplified by NiV polymerase. The lower panel shows the corresponding positive-sense antigenome RNA and CAT mRNA generated by the NiV polymerase. (F) Quantification of levels of antigenome (gray bars) and minigenome (hatched bars) from replicates of the northern blots shown in (E). The bars show the mean and SD from three independent experiments for each mutant.

Western blot analysis confirmed that each of the L mutants was efficiently expressed relative to WT L protein (Figures S6L and S6O). Primer extension analysis of the RNA generated by the L mutants showed a range of phenotypes. Substitution of T1341 and Q1355 had minimal effect on RNA initiated from the 3′ end of the le or the first gs signal; by contrast, substitution of F1358 completely inhibited both antigenome and mRNA initiation (Figures 7A and 7B). Substitution of N1344 and L1345 inhibited antigenome initiation from the 3′ end of the le region to less than 20% of WT levels but had only a modest inhibitory effect on mRNA initiation from the gs signal (Figures 7A and 7B; note that in Figure 7A the upper and lower panels are derived from the same gel, allowing direct comparison of RNA levels within the same reaction). Northern blot analysis of mRNAs generated in the single-step minigenome assay confirmed that the T1341A, N1344A, L1345A, and Q1355A L mutants were all capable of mRNA transcription, albeit at moderately reduced levels compared with WT polymerase (Figures 7C and 7D). Luciferase assays showed a similar pattern of gene expression by each of the mutants as was determined by measuring mRNA levels with their activities being ranked as follows: T1341A > Q1355A > N1344A > L1354A > H1347A/R1348A and F1358A (Figure S6Q). Curiously, the levels of luciferase activity for the T1341A, Q1355A, N1344A, and L1354A mutants relative to WT L were slightly lower than the relative levels of mRNA (compare Figure S6Q with Figures 7B and 7D). It is possible that these intrusion loop mutations affect the nature of the cap that is added and/or inhibit cap methylation, thus impairing mRNA translation, although further research is required to test these hypotheses.

Assessment of the L mutants in the multi-cycle minigenome system confirmed that N1344A, L1345A, and F1358A mutants were highly defective in RNA replication (Figures 7E and 7F). These mutant L proteins also failed to generate detectable mRNA in the multi-cycle minigenome system (Figures 7E and 7F). This is likely because the inhibitory effect of the mutations on RNA replication would have significantly reduced the amount of properly encapsidated minigenome template that was available in these reactions compared with the reactions with WT L protein (note that there is a large difference in the amount of encapsidated (nuclease-resistant) minigenome template available if the minigenome can be replicated, e.g., compare the L WT + MC and L WT + SS samples in Figures S6C and S6E). These results show that the intrusion loop has two separate and distinguishable roles in mRNA transcription and genome replication.

## Discussion

NiV depends on its L-P polymerase complex to perform all the enzymatic activities required to generate capped and polyadenylated mRNAs and replicative RNAs. Here we determined the structure of the L-P complex and performed functional analysis of some features that are shared with other nsNSV polymerase and features that have been specifically observed in the NiV polymerase.

Both transcription and RNA replication are dependent on the polymerase being able to associate with the template RNP complex, dissociate N protein from the RNA, and feed the RNA into the template entry channel. It is thought that P plays a key role in this process. We detected a region of one of the P molecules, P4, snaking along the surface of L on the opposite face of the polymerase from P2-XD and close to the template entry channel (Figure 4A). The Q454R/C457E double substitution, which we used to disrupt the L interaction with P4, did not affect L protein expression (Figures S6J and S6M) but reduced transcription and RNA replication compared with WT levels (Figures 6 and S6R). These findings suggest that this L-P interface plays an equivalent role in both processes. The P4 extension binding close to the template entrance channel might allow P4-XD to play a role in helping to guide the RNP toward the template entrance channel.

Another feature that might be involved in guiding RNA is the palm insert (amino acids 603–709), which is close to the putative nascent RNA exit channel (Figures 2E–2G). Studies with other paramyxovirus polymerases have shown this region is important for polymerase activity but did not distinguish if it affected transcription and/or replication.^27^^,^^50^ Here, we show that the NiV palm insert plays a key role during transcription and RNA replication (Figures 6 and S6R). Interestingly, the corresponding palm insert region of PIV3 L is also largely disordered, but the C-terminal segment of the region forms a β-strand that augments a β-sheet in the CTD (“β-latch”) that may restrict the conformation of the L C-terminal globular domains.^27^ In the NiV L-P structure, the palm insert and the C-terminal globular domains are not visible; by analogy to the PIV3 L structure, however, the C-terminal region of the NiV L RdRp palm insert could interact with the C-terminal globular domains.

The findings presented here demonstrate the multifunctional nature of the NiV L protein CAP domain. By analogy with the rhabdovirus polymerases, the CAP domain contains residues necessary for the GTPase and PRNTase steps of cap addition^12^ and is expected to be required for mRNA transcription. However, studies presented here show the importance of the CAP domain for RNA replication in addition to transcription. We examined the roles of two ZF motifs in CAP, which are conserved among many nsNSVs (Figures 3A–3C and S3A–S3C). As noted above, ZF1 appears to aid structural integrity of the L protein. However, mutations in both ZF motifs reduced the relative amounts of a truncated form of L, which is presumably generated by cleavage at the palm insert (Figure S6K). This observation could suggest direct or long-range interactions between the ZF motifs and the palm insert or that the ZF motifs influence the conformational dynamics of the RdRp core. Substitutions in each of the ZF motifs inhibited replication in addition to transcription (Figures 6 and S6R), suggesting that they might play a role common to both processes. Intriguingly, a structure of the VSV polymerase in association with RNP within virions showed that it is oriented such that the ZF motifs of the CAP are proximal to the N protein of the RNP.^35^ Thus, it is possible that the ZF motifs aid polymerase association with the RNP template.

The other feature of the CAP domain that is required for both transcription and replication is the intrusion loop. The intrusion loop can be identified as a loop in all nsNSV polymerase structures that have been determined so far (Figures S3D–S3I).^27^^,^^37^^,^^38^^,^^39^^,^^41^^,^^42^ The intrusion and priming loops are less ordered in the NiV L protein structure than in other paramyxovirus L protein structures (Figures 3F and S3D–S3F). Here, we examined the role of the intrusion loop with a panel of substitution mutants. Two of the mutants that were tested were completely defective in mRNA transcription, namely the H1347A/R1348A and F1358A mutants (Figures 6, 7, and S6R). The HR motif catalyzes the PRNTase reaction required for mRNA cap addition,^12^ and so the H1347A/R1348A mutation could inhibit transcription by inhibiting cap addition. The mechanism of transcription inhibition by the F1358A mutation is not known. Both the HR motif and F1358 also play a role in RNA replication (which does not involve a cap addition step) (Figures 6, 7, and S6R). Likewise, other mutations in the intrusion loop, N1344A, L1345A, and Q1355A, also inhibited RNA replication, with Q1355A having a more minor effect. Importantly, the N1344A and L1345A mutations had a more profound inhibitory effect on RNA replication than mRNA transcription (Figures 7A, 7B, and S6R).

While transcription and replication are distinct processes, many of the initial steps are shared, including template binding, promoter recognition, and RNA synthesis initiation at the 3′ end of the le region and nascent RNA elongation. The observation that the N1344A and L1345A mutants could perform each of these steps during transcription suggests that they were defective in a step that is specific to RNA replication. These findings are reminiscent of findings made with RSV polymerase, which showed that substitution of the threonine residue of the GxxT motif in the priming loop (T1267) or the arginine residue of the HR motif of the intrusion loop (R1339) inhibited RNA replication.^51^ Analysis of the abortive replicative RNAs generated by the RSV L T1267A and R1339A mutants showed that both mutants could initiate RNA replication at le position 1U, but rather than elongating the products to make antigenome RNA, they released the RNA before they reached the end of the le promoter region.^51^ Given that elongation during RNA replication is thought to be facilitated by concurrent encapsidation, it seems likely that mutations in the intrusion loop (and the priming loop in the case of RSV) either directly or indirectly inhibit encapsidation of the nascent replicative RNA. We suggest that, given that the intrusion loop (including the residues we mutated for functional analyses) is adjacent to the nascent RNA exit channel (Figures S3K and S3L), it might help to guide the nascent RNA appropriately for encapsidation.

Perhaps the most distinctive feature of the complex compared with that of other nsNSV L-P structures is the ⍺-helical cap structure at the N-terminal end of the P OD (Data S2). Both cryo-EM 3D variability analysis and molecular dynamics analysis suggest that the tip of the long coiled-coil that is capped by a bundle of helices is flexible. This flexibility could be important during RNA replication, in which it is thought that a region near the N terminus of P molecules that are associated with L delivers unassembled N protein onto the nascent RNA.

In summary, we have determined the structure of NiV L-P complex and performed functional analyses to identify features that play key roles in transcription and replication. These findings enhance our understanding of nsNSV polymerases and the mechanisms by which they engage in transcription or RNA replication and have the potential to allow for rational drug design against NiV infection.

### Limitations of the study

The *in vitro* RNA synthesis assay we performed in purified NiV L-P complexes involves a naked RNA oligonucleotide template rather than encapsidated RNA. While this assay is suitable for showing if the NiV L-P complex is active, the polymerase might behave differently on this template compared with an encapsidated RNA template. While we could not resolve the C-terminal globular domains of NiV L and the intrusion and priming loops, likely due to flexibility, additional studies will be required to determine whether binding of ligands, including nucleotides and/or nucleic acids, causes these regions to become ordered.

## Resource availability

### Lead contact

Further information and requests for resources and reagents should be directed to and will be fulfilled by the lead contact, Jonathan Abraham (jonathan_abraham@hms.harvard.edu).

### Materials availability

Reagents generated in this study are available from the lead contact upon request with completed material transfer agreements.

### Data and code availability

• Protein Data Bank (PDB) and Electron Microscopy Data Bank (EMDB) identification numbers for the cryo-EM structures and maps reported in this manuscript are available as of the date of publication. Identification numbers are listed in the key resources table. The per-residue root-mean-square-fluctuation (RMSF) values from MD simulations reported in this study are provided in Data S4.
• This paper does not report original code.
• Any additional information required to reanalyze the data reported in this paper is available from the lead contact.

## Acknowledgments

Cryo-EM data were collected at the Harvard Cryo-EM Center for Structural Biology at Harvard Medical School. We would like to thank Dr. Bernard Moss (National Institutes of Health) for providing the pTM1 plasmid and Dr. Klaus Conzelmann (Ludwig-Maximilians-University Munich) for providing the BSR-T7 cells used for the minigenome assays. We thank Dr. Bridget Gollan for her help with generating illustrations for the graphical abstract. Y.B. received funding support from the National Natural Science Foundation of China (Excellent Young Scientists Fund [Overseas] and 82404516) and the Shanghai Municipal Commission of Education. This work was supported by an award from the Bill & Melinda Gates Foundation (INV-040438) to R.F.

## Author contributions

Conceptualization, S.H., H.K., P.Y., J.P., Y.B., R.F., and J.A.; investigation, S.H., H.K., P.Y., Z.Y., B.L., S.M., J.P., Y.B., R.F., and J.A.; writing – original draft, S.H., H.K., P.Y., Y.B., R.F., and J.A.; writing – review and editing, S.H., H.K., P.Y., Z.Y., B.L., S.M., J.P., M.S., Y.B., R.F., and J.A.; funding acquisition, M.S., R.F., and J.A.

## Declaration of interests

R.F. is the recipient of a sponsored research agreement with Merck & Co.

## STAR★Methods

### Key resources table

**Table:** 

REAGENT or RESOURCE	SOURCE	IDENTIFIER
**Antibodies**		

β-tubulin rabbit polyclonal antibody	LI-COR	Cat# NC9872627
Strep-Tag Classic antibody (Strep-tag II)	Bio-Rad	Cat# MCA2489, RRID: AB_609795
IRDye 680RD Donkey anti-rabbit IgG secondary antibody	LI-COR	Cat# 926-68073, RRID: AB_10954442
IRDye 800CW Goat anti-mouse IgG secondary antibody	LI-COR	Cat# 926-32210, RRID: AB_621842

**Bacterial and virus strains**		

MAX Efficiency DH10Bac™ cells	Thermo Fisher Scientific	Cat# 10361012

**Chemicals, peptides, and recombinant proteins**		

Biotin	Sigma-Aldrich	Cat # B4501-5G
cOmplete™, Mini, EDTA-free protease inhibitor cocktail	Millipore Sigma	Cat# 11836170001
Dithiothreitol (DTT)	Sigma-Aldrich	Cat# 10708984001
Strep-Tactin®XT 4Flow® high-capacity resin	IBA Lifesciences	Cat# 2-5030-010
NiV (Bangladesh strain) L (full-length, with C-terminal triple strep tag)-P (full-length, with N-terminal polyhistidine tag)	This paper	N/A
NiV L (Bangladesh stain) L (full-length, with residues 604–704 deleted -triple strep-tag)- P (full-length, with N-terminal polyhistidine tag)	This paper	N/A
[α^32^P] GTP, 250 μCi	Revvity	Cat# BLU006H250UC
NTP 100 mM each	Thermo Fisher Scientific	Cat# R0481
Calf intestinal alkaline phosphatase	New England Biolabs	Cat# M0525
Lipofectamine 3000 Transfection reagent	Invitrogen	Cat# L3000015
Aprotinin	Thermo Fisher Scientific	Cat# J11388MA
Micrococcal nuclease S7	Roche	Cat# 10107921001
[ɣ^32^P] ATP, 250μCi	Revvity	Cat #BLU002A250UC
Sensiscript reverse transcriptase	QIAGEN	Cat# 205211
Moloney murine leukemia virus reverse transcriptase	Promega	Cat# M1701
AccuGel 19:1 (40%)	National Diagnostics	Cat# EC-850
Tris-Borate-EDTA	Fisher BioReagents	Cat# BP1333-1
Urea	Sigma	Cat# U6504
T4 Polynucleotide kinase	New England Biolabs	Cat# M0201

**Critical commercial assays**		

Promega Dual-Luciferase Reporter Assay System	Promega	Cat# E1910
Monarch total RNA Miniprep kit	New England Biolabs	Cat# T2010S

**Deposited data**		

Cryo-EM map of NiV L-P complex	This paper	EMD-44465
Model of NiV L-P complex	This paper	PDB ID: 9BDQ

**Experimental models: Cell lines**		

Sf9 cells (Spodoptera frugiperda)	ThermoFisher	Cat# 11496015, RRID: CVCL_JF76
BSR-T7 cells	Dr. Klaus Conzelmann	N/A

**Oligonucleotides**		

NiV (Bangladesh strain) leader sequence 1-17 RNA (5' AUUUUCCCUUGUUUGGU 3')	Integrated DNA technologies	N/A
NiV (Bangladesh strain) positions 90-109 primer (5’ GATATATTTCTTGAGGATCC 3’), PAGE purified	Invitrogen	N/A
DynaMarker prestain marker for RNA high	Diagnocine	Cat# DM260S
Single-stranded DNA ladder	Simplex Sciences	Cat# ss20

**Recombinant DNA**		

pFastbac Dual vector	Thermofisher	Cat# 10712024
NiV (Bangladesh strain) L (full-length, with C-terminal triple strep tag)-P (full-length, with N-terminal polyhistidine tag) in pFastBac dual vector	This paper	N/A
NiV (Bangladesh stain) L (full-length, with residues 604–704 deleted triple strep-tag-P (full-length, with N-terminal polyhistidine tag) in pFastBac dual vector	This paper	N/A
Multi-cycle minigenome with CAT and Renilla luciferase reporter gene	This paper	N/A
Single-step minigenome with a deletion of promoter element 2 within the L nontranslated region and substitutions at position 1-4 relative to the 5' end of the trailer region	This paper	N/A
pTM1 plasmid	Dr. Bernard Moss	N/A
Codon optimized NiV (Bangladesh strain) L open reading frames in modified pTM1 containing T7 promoter and internal ribosome entry site with Strep tag on C terminus	This paper	N/A
Codon optimized NiV (Bangladesh strain) P open reading frames in modified pTM1 containing T7 promoter and internal ribosome entry site	This paper	N/A
Codon optimized NiV (Bangladesh strain) N open reading frames in modified pTM1 containing T7 promoter and internal ribosome entry site	This paper	N/A
NiV (Bangladesh strain) L (with D832A) open reading frames in modified pTM1 containing T7 promoter and internal ribosome entry site with Strep tag on C terminus	This paper	N/A
NiV (Bangladesh strain) L (with H1347A and R1348A) open reading frames in modified pTM1 containing T7 promoter and internal ribosome entry site with Strep tag on C terminus	This paper	N/A
NiV (Bangladesh strain) L (with Q454R and C457E in pTM1) open reading frames in modified pTM1 containing T7 promoter and internal ribosome entry site with Strep tag on C terminus	This paper	N/A
NiV (Bangladesh strain) L (with residues 604–704 deleted) open reading frames in modified pTM1 containing T7 promoter and internal ribosome entry site with Strep tag on C terminus	This paper	N/A
NiV (Bangladesh strain) L (with C1236S and C1239S) open reading frames in modified pTM1 containing T7 promoter and internal ribosome entry site with Strep tag on C terminus	This paper	N/A
NiV (Bangladesh strain) L (with C1428S and C1429S) open reading frames in modified pTM1 containing T7 promoter and internal ribosome entry site with Strep tag on C terminus	This paper	N/A
NiV (Bangladesh strain) L (with T1341A) open reading frames in modified pTM1 containing T7 promoter and internal ribosome entry site with Strep tag on C terminus	This paper	N/A
NiV (Bangladesh strain) L (with N1344A) open reading frames in modified pTM1 containing T7 promoter and internal ribosome entry site with Strep tag on C terminus	This paper	N/A
NiV (Bangladesh strain) L (with L1345A) open reading frames in modified pTM1 containing T7 promoter and internal ribosome entry site with Strep tag on C terminus	This paper	N/A
NiV (Bangladesh strain) L (with Q1355A) open reading frames in modified pTM1 containing T7 promoter and internal ribosome entry site with Strep tag on C terminus	This paper	N/A
NiV (Bangladesh strain) L (with F1358A) open reading frames in modified pTM1 containing T7 promoter and internal ribosome entry site with Strep tag on C terminus	This paper	N/A

**Software and algorithms**		

UCSF Chimera 1.15	Pettersen et al.^52^	https://www.cgl.ucsf.edu/chimera/, RRID:SCR_004097
UCSF ChimeraX 1.5	Goddard et al.^53^	https://www.cgl.ucsf.edu/chimerax/, RRID:SCR_015872
PyMOL 2.5.5	The PyMOL Molecular Graphics System, Version 3.0 Schrödinger, LLC.	https://pymol.org/2/, RRID:SCR_000305
Phenix 1.20.1-4487	Adams et al.^54^	https://www.phenix-online.org, RRID: SCR_014224
SerialEM 4.1-beta	Mastronarde^55^	http://bio3d.colorado.edu/SerialEM/, RRID: SCR_017293
MotionCor2 1.6.4	Zheng et al.^56^	https://emcore.ucsf.edu/cryoem-software, RRID: SCR_016499
CTFFind4 4.1.14	Rohou and Grigorieff^57^	http://grigoriefflab.janelia.org/ctffind4, RRID: SCR_016732
Relion 3.1.1	Zivanov et al.^58^	https://www3.mrc-lmb.cam.ac.uk/relion/index.php/Main_Page, RRID: SCR_016274
cryoSPARC v4.5.1	Punjani et al.^59^	https://cryosparc.com/, RRID: SCR_016501
cryOLO 1.9.9	Wagner et al.^60^	https://cryolo.readthedocs.io/en/stable/index.html, RRID: SCR_018392
DeepEMhancer 20220530_cu10	Sanchez-Garcia et al.^61^	https://github.com/rsanchezgarc/deepEMhancer, RRID: SCR_022573
*Coot* 0.9	Emsley et al.^62^	http://www2.mrc-lmb.cam.ac.uk/personal/pemsley/coot/, RRID: SCR_014222
MolProbity 4.5.2	Williams et al.^63^	http://molprobity.biochem.duke.edu, RRID: SCR_014226
Refeyn DISCover^MP^ v2.3	REFEYN	https://www.refeyn.com/
Maestro 2024–3	Schrodinger	https://www.schrodinger.com/maestro, RRID: SCR_016748
Image Studio Lite version 5.2	LI-COR	https://www.licor.com/bio/image-studio-lite/, RRID: SCR_013715
Prism 10 version 10.3.1	GraphPad Prism	https://www.graphpad.com, RRID: SCR_002798
UNICORN™ version 7.8	Cytiva	https://www.cytivalifesciences.com/en/us/shop/chromatography/software/unicorn-7-p-05649
AlphaFold Server	Abramson et al.^26^	https://deepmind.google/technologies/alphafold/alphafold-server/, RRID: SCR_025885
CAVER web version 1.2	Stourac et al.^28^	https://loschmidt.chemi.muni.cz/caverweb/
Clustal Omega	Sievers et al.^30^	https://www.ebi.ac.uk/jdispatcher/msa/clustalo, RRID: SCR_001591

### Experimental model and study participant details

#### Insect cells

Sf9 cells (*Spodoptera frugiperda*) (Thermo Fisher Scientific Cat# 11496015) cells used for recombinant NiV L-P production were maintained in Sf-900™ II SFM (Thermo Fisher Scientific Cat# 10902088) media at 27°C.

#### Bacterial cells

*Escherichia coli* MAX Efficiency DH10Bac™ cells (Thermo Fisher Scientific Cat# 10361012) were used for bacmid production to produce recombinant baculoviruses.

#### Cell lines

BSR-T7 cells were maintained Glasgow’s MEM (Thermo Fisher Scientific Cat# 11710035) with 10% (v/v) Fetal Bovine Serum (Thermo Fisher Scientific Cat# A5256701), 1% (v/v) MEM Non-Essential Amino Acids Solution (Thermo Fisher Scientific Cat# 11140050), 1% (v/v) GlutaMAX™ (Thermo Fisher Scientific Cat# 35050061), and 1 mg/ml of Geneticin (G418 sulfate) (Thermo Fisher Scientific Cat# 10131035) at 37 °C, 5–10% CO_2_, and 100% relative humidity.

### Method details

#### Cloning and insect cell expression of NiV L-P complexes

The coding sequence of NiV L (GenBank: AAY43917.1) and P (GenBank: AAY43912.1) were synthesized and codon-optimized for Bac-to-Bac expression system using pFastBac Dual transfer vector. The sequences of NiV L and P were fused with a C-terminal triple-strep tag after a TEV protease cleavage site and an N-terminal His_6_ tag followed by a TEV protease cleavage site, respectively. NiV L was inserted downstream of the polyhedrin promoter and P was inserted downstream of the p10 promoter in the pFastBac Dual. Recombinant bacmid containing NiV L and P genes was isolated after transforming into MAX Efficiency DH10Bac™ competent cells (Thermo Fisher Scientific Cat# 10361012) following the user guide of the Bac-to-Bac Baculovirus Expression System (Invitrogen). Viral stock generated from purified bacmids was amplified and used for protein expression. Two liters of *Spodoptera frugiperda* 9 (Sf9) cells (Thermo Fisher Scientific Cat# 11496015) maintained in Gibco™ Sf-900TM II SFM media (Thermo Fisher Scientific Cat# 10902104) at a density of 2.5x10^6^ cells/ml were infected with amplified viruses to co-express NiV L and P proteins at 27°C for 72 h.

#### NiV L-P complex protein purification

The pellet of Sf9 cells expressing the NiV L-P complex was resuspended in lysis buffer (50 mM Tris, pH 7.5, 500 mM NaCl, 10% (v/v) glycerol, 6 mM MgSO_4_, 1 mM dithiothreitol (DTT), 1% (v/v) Triton X-100) supplemented with cOmplete™, EDTA-free protease inhibitor cocktail (Sigma-Aldrich Cat# 11836170001), carried out for 5 min at 4°C. After high-speed centrifugation at 50,000 g for 2 h at 4°C, the supernatant containing the target proteins was incubated with Strep-Tactin® XT Sepharose resin (IBA Lifesciences Cat# 2-5030-010) at 4°C for over 1 h and then loaded onto a gravity column. The beads were washed using buffer A (50 mM Tris pH 8.0, 500 mM NaCl, 10% glycerol (v/v), 2 mM MgSO_4_, 1 mM DTT), and the bound proteins were eluted using 50 mM biotin in buffer A. The eluates were analyzed by sodium dodecyl sulfate-polyacrylamide gel electrophoresis (SDS-PAGE) and the putative L and P bands were subjected to LC-MS/MS analysis (Harvard Center for Mass Spectrometry) to confirm their identities. Specifically, the NiV L band that is near the 250 kDa marker band on SDS-PAGE (Figure S1A) was cut and sent for trypsin digestion, followed by LC-MS/MS sequencing. Fragments of NiV L detected by LC-MS/MS are highlighted in gray in Data S1. NiV L has a coverage over 82%. The sequence C-terminal of residue 2244 is a glycine-serine linker and triple-strep tag. Additionally, the NiV P band near the 100 kDa marker band on SDS-PAGE (Figure S1A) was cut and sent for LC-MS/MS sequencing. Fragments detected are highlighted in gray in Data S1. NiV P has a coverage over 94%. The sequence N-terminal of residue 1 is a histidine tag and serine-glycine linker.

The fractions containing L-P protein were further purified using a size-exclusion column (Superose 6 Increase 10/300 GL, GE healthcare) equilibrated with size exclusion chromatography (SEC) buffer (20 mM Tris, pH 7.5, 500 mM NaCl, 2 mM MgSO_4_, 1 mM DTT) using an ÄKTA pure™ protein purification system (Cytiva) with UNICORN™ version 7.8. The peak fractions near 11 ml were pooled and concentrated. Protein homogeneity was examined by negative stain electron microscopy and mass photometry (see below).

#### Mass photometry analysis of NiV L-P protein

Mass photometry analyses were carried out with a Refeyn Two^MP^ mass photometer (Refeyn LTD, Oxford, UK) at room temperature. Glass coverslips and gaskets were cleaned with HPLC-grade water and isopropanol and dried under filtered gas before use. NiV L-P was diluted to 200 nM in SEC buffer. Eighteen microliters of buffer were used to find the camera focus prior to loading 2 μl of the sample onto the gasket. Acquisition camera image size was set to medium. Data were collected as a 1 min movie and then processed using ratiometric imaging. To correlate ratiometric contrast to mass, the Refeyn Two^MP^ instrument was calibrated using molecular standards of monomeric bovine serum albumin (BSA) (66 kDa), dimeric BSA (132 kDa), and thyroglobulin (MW 660 kDa) with a molecular weight error less than 5%. The experiment was performed twice. Data were analyzed using Discover^MP^ version 2.3 software (Refeyn LTD, Oxford, UK).

#### In vitro RNA synthesis assay

L protein was quantified by SDS-PAGE and densitometric comparison with a BSA standard curve on the same gel. L-P complexes (8–90 nM final concentration) were incubated with 2 μM NiV le 1–17 template with the sequence 3'-UGGUUUGUUCCCUUUUA-5' in a buffer containing 50 mM Tris-HCl, pH 7.5, 8 mM MgCl_2_, 5 mM DTT and 10% glycerol (v/v) for 10 min at 30°C. Reactions were initiated by the addition of ATP and CTP to a final concentration of 1 mM each and 10 μCi of [α-^32^P]-GTP tracer (Revvity, 3000 Ci/mmol and 10 μCi/μl, final concentration 100–150 nM) in a total reaction volume of 50 μl and allowed to proceed for 1 h at 30°C. The polymerase complex was inactivated by heating to 95°C for 5 min. Reactions were subsequently treated with 10 U of calf intestinal alkaline phosphatase (NEB, 10,000 U/ml) for 1 h at 37°C. 200 μl of 0.25% SDS in nuclease-free water were added to each and RNA products were isolated by extraction with 250 μl of acid-phenol:chloroform (Invitrogen). After the addition of 170 mM NaCl and 15 μg glycogen (Invitrogen), RNA products were precipitated with ethanol overnight at -20°C. Pellets were washed once with ice-cold 70% (v/v) ethanol, briefly air-dried and resuspended in 8 μl water. An equal volume of 2 x STOP buffer (20 mM EDTA, 0.01% each bromophenol blue and xylene cyanol in deionized formamide) was added before heat denaturing samples for 5 min at 95°C. Samples were flash cooled on ice and loaded onto a 20% acrylamide sequencing gel containing 7 M urea in Tris-borate-EDTA buffer. Gels were vacuum dried at 80°C onto Whatman 3MM paper. RNA products were visualized by phosphor imaging (Typhoon IP, GE Healthcare Life Technologies).

#### Cryo-EM sample preparation and data collection

An aliquot of 4 μl of NiV L-P complex at 0.7 mg/ml was applied to a freshly glow-discharged Quantifoil Cu 1.2/1.3 400 mesh grid (Electron Microscopy Sciences Cat# Q4100CR1.3). The sample was blotted for 3 s after incubation for 15 s at 4°C with a relative humidity of 100%, then plunge-frozen in liquid ethane using Vitrobot Mark IV (Thermo Fisher Scientific, USA), and stored in liquid nitrogen. Cryo-EM data were collected on a Titan Krios microscope (Thermo Fisher Scientific, USA) (300 kV) equipped with a K3 Summit direct electron detector (Gatan, USA) at the Harvard Cryo-Electron Microscopy Center for Structural Biology. Movie stacks were automatically recorded using SerialEM 4.1-beta^55^ in counting mode at a nominal magnification of 105,000 x, corresponding to a physical pixel size of 0.83 Å. The defocus was set to from -1.0 to -2.5 μm. A total exposure dose of 53 e^-^/Å^2^ was fractionated into 50 frames for each movie stack. We obtained one cryo-EM dataset of NiV L-P complex including a total number of 7803 movie stacks.

#### Cryo-EM image processing

All images were processed using Relion 3.1,^58^ movie frames were gain-normalized and motion-corrected using MotionCor2 version 1.6.4^56^, and contrast transfer function (CTF) correction was performed using CTFFind4 version 4.1.14,^57^ as implemented in Relion 3.1. Particle-picking was performed in crYOLO version 1.9.9^60^ and 1,871,392 particles from 7,803 micrographs were picked. Picked particles were imported to Relion, extracted, binned to a pixel size of 1.66 Å and subjected to 2D classification. Good classes of particles from the 2D classification were selected and imported to cryoSPARC version 4.5.1^59^ to generate an initial model. Three rounds of 3D classification with C1 symmetry were imposed on 1,842,231 particles, 477,936 particles of which were selected and subjected to auto-refinement to generate a 2.7 Å map. CTF refinement and Bayesian polishing were performed to improve the resolution. Consequently, a final map was generated to 2.3 Å. To improve the local resolution of P ⍺-helical cap structure, focused 3D auto-refinement was performed, allowing us to obtain a masked a 3.0 Å map. We also used DeepEMhancer version 20220530_cu10^61^ for cryo-EM volume post-processing.

#### Model building and refinement

The structures of the five domains (RdRp, CAP, CD, MTase domain and CTD domain) of L and of monomeric P were predicted using Phyre2 version 2.0.^64^ These predicted structures and the crystal structure of the NiV P OD (PDB code: 4N5B,^15^ residues 476–576) were used to build the atomic model of NiV L-P complex. The RdRp and CAP domains of L and tetrameric P OD plus a single P XD were docked and rigid-body fitted well into the cryo-EM map using UCSF Chimera. Extra residues of P were built manually in *Coot* version 0.9.^62^ Based on interpretable density, we could model L residues 5–1463, with the exception of residues 601–709, 1148–1153, 1266–1289 (priming loop), and 1342–1362 (intrusion loop). We could model P1 residues 479–584, P2 residues 479–708 (except for residues 581–591 and 612–630), P3 residues 477–579, and P4 residues 479–596. We performed manual model building to improve local fit using *Coot*^62^ and real space refinement using Phenix.^54^ MolProbity^63^ was used to validate the model. Statistics are provided in Table S1.

#### 3D variability analysis

3D variability analysis (3DVA) was performed using cryoSPARC.^43^^,^^59^ Particle stacks and reference map from Relion final 3D auto-refine were imported. 3DVA was performed using a mask generated from Relion 3.1. Twenty components were generated, of which major components showed flexibility in the P segment. The frames from these components were visualized and recorded in UCSF Chimera^52^ as a volume series (see Video S1).

#### Negative stain electron microscopy

For negative stain electron microscopy experiments, 4 μl of purified protein samples were applied to glow-discharged continuous carbon films supported by 400-mesh copper grids (Electron Microscopy Sciences Cat#: FCF400-Cu-50) stained with 1.5% (w/v) uranyl formate. Stained grids were imaged on a Philips CM10 transmission electron microscope (100 kV) equipped with a Gatan UltraScan 894 (2k x 2k) CCD camera. Images were collected at a magnification of 52,000x (2.06 Å/pixel) and processed using cryoSPARC.^59^ Particles were selected using Blob picker and particles were subjected to several rounds of reference-free alignment and 2D classification.

#### In silico modeling, molecular dynamics simulations and molecular docking

The cryo-EM structure of the NiV L-P complex was prepared before modelling and simulations. The module of Protein Preparation in Schrödinger Maestro^65^ was applied to cap termini, repair residues, optimize H-bond assignments, and run restrained minimizations following default settings.

The Schrödinger Desmond MD engine 2024–3^66^ was used for simulations.^67^ An orthorhombic water box was applied to bury prepared protein systems with a minimum distance of 10 Å to the edges from the protein. Water molecules were described using the SPC model. Na^+^ ions were placed to neutralize the total net charge. All simulations were performed following the OPLS4 force field.^68^ The ensemble class of NPT was selected with the simulation temperature set to 300K (Nose-Hoover chain) and the pressure set to 1.01325 bar (Martyna-Tobias-Klein). A set of default minimization steps pre-defined in the Desmond protocol was adopted to relax the MD system. The simulation time was set to 100 ns for the protein system with three duplicate MD runs. One frame was recorded per 200 ps during the sampling phase. Post-simulation analysis of the RMSF was performed using a Schrödinger simulation interaction diagram. RMSF values from the Cα of each residue were used for plotting.

The molecular docking was performed using the Glide module in Schrödinger. The binding pocket on NiV L was defined by selecting residues E922, S925, S928, Y1001, I1068, and H1165/Y1165 along with surrounding residues. Similarly, the binding pocket on PIV3 L (PDB: 8KDC)^27^ was defined by selecting residues E863, S866, S869, Y942, I1009, T1010, and Y1106 along with surrounding residues. The 3D conformation of the compound GHP-88309 was prepared using LigPrep. Glide SP, in standard precision mode, was used without constraints to generate binding poses. Up to 20 docked poses were allowed to be generated for GHP-88309 inside the defined pockets for each protein. Among these poses, the top 10 poses underwent the post-docking minimization. The prior study by Cox et al.^44^ was referred to guide the visual inspection of minimized poses. Representative conformations that could reproduce the predicted binding pose^44^ were selected and investigated to be reported in this manuscript.

#### AlphaFold 3 modeling

Predicted structures for RNA-bound NiV L (Bangladesh strain, GenBank: AAY43917.1) or PIV3 L (GenBank: WCF97250.1) were generated using AF3.^26^ Modeling was performed using the leader RNA 1–16 (underlined) plus six adenines as the template RNA (5′-UUUUCCCUUGUUUGGUAAAAAA-3′ for NiV and 5′-UCUUCUCUUGUUUGGUAAAAAA-3′ for PIV3) and the complementary sequence of leader RNA 1–9 (underlined) plus three adenines as the nascent RNA (5′-AAAACCAAACAA-3′) along with a GTP molecule, two magnesium ions, and two zinc ions. Note that non-specific adenines were added to the template and nascent RNAs to ensure that their 3′ and 5′ ends, respectively, remained single stranded in the model.

#### Design and cloning of the NiV minigenome system

The minigenome system was designed based on sequences from the NiV Bangladesh strain (GenBank: AY988601.1). The cis-acting elements inserted into the multi-cycle replication minigenome were determined based on a previously described NiV minigenome template.^10^ The multi-cycle minigenome contained in 3' to 5' order, the first 112 nt of the NiV genome sequence, including the 55 nt leader region, *N* gene start signal, and 46 nt of non-translated sequence from the 3' end of the *N* gene, which includes promoter element 2 (CNNNNN)_3_, 519 nt derived from the bacterial *chloramphenicol acetyltransferase* (*CAT*) gene, 105 nt of sequence derived from the *N-P* gene junction region (nucleotides 2247–2351 of the NiV Bangladesh complete genome) that includes the *N* gene end signal, trinucleotide intergenic, and *P* gene start signal, followed by 939 nt *Renilla luciferase* reporter gene sequence, and then the 5' 100 nt of the NiV genome, including 56 nt of non-translated sequence from the 5' end of the L gene, which contains the complement of promoter element 2, the *L* gene end signal and the 30 nt 5' trailer region. Together with inserted restriction sites, the total minigenome length is 1794 nt. The minigenome cassette was flanked by a hepatitis delta virus ribozyme sequence adjacent to the leader region and a T7 promoter sequence adjacent to the trailer region (Figure S6A). This minigenome was used as a template to generate a single-step minigenome limited to the antigenome synthesis step of replication. This minigenome had a deletion of promoter element 2 within the L nontranslated region and substitutions at position 1–4 relative to the 5' end of the trailer region, which inactivated the trailer promoter at the 3' end of the antigenome and created an optimal T7 promoter sequence (Figure S6A). The multi-cycle minigenome followed the “rule of six” except for two additional G residues contributed by the T7 promoter; the single-step minigenome followed the rule of six including the additional G residues contributed by the T7 promoter. Codon optimized versions of NiV Bangladesh strain N, P, and L open reading frames (Synbio) were inserted into a modified version of plasmid pTM1 (a kind gift from Dr. Bernard Moss),^69^ which contains a T7 promoter and internal ribosome entry site. Each open reading frame was inserted such that the initiating codon was inserted into the plasmid NcoI site, and the 3′ end of the open reading frame was flanked with a 17 nt poly A sequence.^27^ In addition, the open reading frame of L contained a C-terminal strep tag. All plasmids were sequenced in their entirety (Plasmidsaurus) and their integrity and purity was confirmed by agarose gel electrophoresis prior to each transfection.

#### Reconstitution of minigenome replication and transcription

BSR-T7 cells (a kind gift from Dr. Klaus Conzelmann)^70^ in six-well dishes were transfected with (per well) 0.3 μg of the relevant minigenome plasmid, 0.4 μg of N, 0.2 μg of P, 0.1 μg of L and 0.04 μg of firefly expression minigenome using Lipofectamine 3000 (Invitrogen Cat# L3000015). Each transfection reaction was set up in duplicate. Followed by incubation at 37°C for 22–24 h, the transfection mixture was replaced with OptiMem containing 2% (v/v) fetal bovine serum. Cells were harvested 44–48 h after transfection.

#### Harvesting of transfected cells for Western blot and luciferase assays

For Western blot and luciferase analyses, cells from one transfected well were lysed in 500 μl passive lysis buffer (Promega). A 100 μl aliquot of the lysate was passed through a QIAshredder column (Qiagen Cat# 79656) and a 14 μl aliquot was subjected to electrophoresis on an 8% SDS-polyacrylamide gel alongside a PageRuler Plus Prestained Protein Ladder (Thermo Fisher Scientific Cat# 26619). Proteins were transferred to nitrocellulose by Western blotting and the blots were probed with beta-tubulin rabbit polyclonal antibody (LI-COR) at 1:1000 and a Strep-tag classic mouse monoclonal antibody (Bio-Rad Cat# MCA2489) at 1:500 dilution, followed by incubation with IRDye 680RD-conjugated donkey anti-rabbit (LI-COR Cat# 926-68073) and IRDye 800CW-conjugated goat anti-mouse (LI-COR Cat# 926-32210) antibodies, each at 1:20,000 dilution. Blots were scanned using an OdysseyCLx imager (LICOR). For quantification a box of equivalent size as used for other bands of the same protein species was drawn at the corresponding position on the -L sample lane and used to set the background to 0. The resulting values were then normalized to the corresponding value from the L WT reaction, which was set to 1.

For luciferase assays, aliquots of cell lysates were diluted 1:30 and assessed for firefly and Renilla luciferase activity using a Dual-Luciferase Reporter Assay System (Promega Cat# E1910) according to the manufacturer’s instructions. Quantification of the relative luciferase activity for each sample was performed by normalizing the Renilla luciferase value to the firefly luciferase value. The -L value was subtracted to account for background. The resulting values were then normalized to the corresponding value from the L WT reaction, which was set to 1.

#### Northern blot and hybridization

For analysis of total intracellular RNA cells from a transfected well were pelleted and RNA was extracted using a Monarch total RNA miniprep kit (NEB Cat# T2010S). In the case of micrococcal treatment, each transfection reaction was set up in duplicate wells. Following harvest, the cell pellet from one well of the duplicate was resuspended in 100 μl of nuclease lysis buffer 10 mM NaCl, 10 mM Tris, pH 7,5, 1.5 mM MgCl_2_, 1% Triton X-100, 0.5% sodium deoxycholate, 10 mM CaCl_2_, 1 μl of aprotinin (Thermo Fisher Scientific Cat# J11388MA) and then lysis buffer from the Monarch total RNA miniprep kit (NEB Cat# T2010S) was added directly. The cell pellet from the other well was resuspended in 100 μl of nuclease lysis buffer and 10 μl of micrococcal nuclease S7 (Roche Cat# 10107921001) was added. The reaction containing micrococcal nuclease was incubated at 30°C for 1 h with occasional shaking, following which the Monarch total RNA miniprep kit (NEB) lysis buffer was added. RNA from the untreated and treated cells was isolated using the Monarch total RNA miniprep kit (NEB) according to the manufacturer’s instructions. Isolated RNA was subjected to denaturing gel electrophoresis alongside a molecular weight ladder (DynaMarker prestain marker for RNA high; Diagnocine Cat# DM260S) in a 1.5% (w/v) agarose gel containing 0.44 M formaldehyde in MOPS buffer. The RNA was transferred to a nylon membrane (Cytiva), which was stained with methylene blue to confirm that equal amounts of RNA were loaded in each lane and to allow visualization of the molecular weight markers. Negative- and positive-sense ^32^P-labled CAT-specific riboprobes corresponding to minigenome (or complementary) sequence were synthesized by T7 RNA polymerase and purified by phenol-chloroform extraction. The appropriate riboprobe was hybridized to the membrane in 6X SSC, 2X Denhardt’s solution, 0.1% SDS, and 100 μg/ml of sheared salmon sperm DNA for a minimum of 18 h at 65°C. The membranes were washed at 65°C in 2X SSC-0.1% SDS for 2 h and in 0.1X SSC-0.1% SDS for 15 min. Phosphorimager analysis was performed using an Azure Sapphire FL Biomolecular Imager (IS4000) and signals were quantified using Image Studio (LI-COR). The CAT mRNA would be expected to be 636 nt (excluding the poly A tail which is heterogeneous in length), the antigenome and genome RNAs would be expected to be 1,776 nt in the case of the single-step minigenome, and the replicated antigenome and genome RNAs would be expected to be 1,794 nt in the case of the multi-cycle minigenome. To quantify the RNA levels, a box of equivalent size as used for other bands of the same RNA species was drawn at the corresponding position on the -L sample lane and used to set the background to 0. The resulting values were then normalized to the corresponding value from the L WT reaction, which was set to 1.

#### Primer extension analysis

Primer extension reactions were carried out using 4–8 μg of total intracellular RNA isolated from transfected cells. RNA was extracted using a Monarch Total RNA Miniprep Kit (NEB). The RNA initiated from position 1U at the 3' end of the le region, and RNA initiated at the first gs signal was detected with a ^32^P-end-labeled primer (5'-GATATATTTCTTGAGGATCC-3'), which corresponds to nucleotides 90-109 from the 3' end of the minigenome le region. Thus, the cDNA products derived from RNA initiated at the 3' end of le or the gs signal would be expected to be 109 nt or 54 nt, respectively. Each reaction was prepared with either Sensiscript reverse transcriptase (QIAGEN Cat# 205211) or Moloney murine leukemia virus reverse transcriptase (Promega Cat# M1701), following the manufacturer’s instructions. Using Sensiscript reverse transcriptase, the RNA samples were reverse transcribed at 50°C for 1.5 hours. With M-MLV, the RNA samples were incubated for at 42°C for 1.5 hours. After incubation with reverse transcriptase, 2X STOP buffer (deionized formamide, 20 mM EDTA, 0.1% (w/v) bromophenol blue, and xylene cyanol) was added. A single-stranded DNA ladder (Simplex Cat# ss20) was end-labeled using T4 Polynucleotide Kinase (NEB Cat# M0201), following the manufacturer's instructions. The primer extension products were then separated by electrophoresis on 8% polyacrylamide gels containing 7 M urea in Tris-borate-EDTA buffer. After electrophoresis, the gels were dried onto 3MM paper using a vacuum drier at 80°C. Phosphorimager analysis was performed using an Azure Sapphire FL Biomolecular Imager (IS4000), and signals were quantified using Image Studio (LI-COR). A box of equivalent size to those used for other bands of the same RNA species was drawn at the corresponding position on the negative control lane and used to set the background to 0. The resulting values were then normalized to the corresponding value from the L WT reaction, which was set to 1.

#### Figure preparation

UCSF Chimera version 1.15, ChimeraX version 1.5, and PyMOL version 2.5.5 (Schrödinger LLC) were used for structure visualization and figure generation. Prism 10 version 10.3.1 (GraphPad Prism) was used to prepare the bar charts shown in Figures 6, 7, and S6.

### Quantification and statistical analysis

The data analysis function in Microsoft Excel (version 16.62) was used to conduct the unpaired, two-tailed Student's t-test to compare mean RMSF values from two groups. Statistical significance was defined as *p* < 0.05. Data from the Image Studio Lite (version 5.2) and luciferase readings were inputted into Prism 10 (GraphPad Prism version 10.3.1) and means and standard deviations were calculated by Prism 10. The details of tests, including the number of replicates, are included in figure legends.

## Supplemental information

Document S1. Tables S1 and S2

Data S1. LC-MS/MS sequencing of purified NiV L and P, related to Figure 1

Data S2. Comparison of nsNSV cryo-EM structures, related to Figure 1

Data S3. Amino acid sequence alignment of nsNSV L proteins, related to Figure 2

Data S4. Per-residue-root-mean-square-fluctuation values from molecular dynamics simulations of NiV L-P reported in this study, related to Figures 1 and S4 List of RMSF values from 100 ns MD simulations are provided for each NiV L (chain A), P1 (chain E), P2 (chain C), P3 (chain P3), and P4 (chain B).
