# Supplementary material for: Structural and functional analysis of the Nipah virus polymerase complex
Source: Cell. 2025 Feb 6;188(3):688–703.e18. doi: 10.1016/j.cell.2024.12.021 (PMC11813165; doi:10.1016/j.cell.2024.12.021)
Supplement: Document S1. Tables S1 and S2 [file mmc1.pdf]

Cell, Volume *188*

Supplemental information

Structural and functional analysis

# of the Nipah virus polymerase complex

Side Hu, Heesu Kim, Pan Yang, Zishuo Yu, Barbara Ludeke, Shawna Mobilia, Junhua Pan, Margaret Stratton, Yuemin Bian, Rachel Fearns, and Jonathan Abraham

Table S1. Data collection, image processing, and model refinement, related to Figure 1.

	NiV L-P complex (PDB: 9BDQ, EMDB: EMD-44465)
**Data collection and processing**	
Microscope	Titan Krios
Detector	K3 Summit direct electron detector
Magnification	105,000 x
Voltage (kV)	300
Electron exposure ($e^-/Å^2$)	53
Defocus range (μm)	-1.0 to -2.5
Pixel size (Å)	0.83
Symmetry imposed	C1
Initial particles	1,871,392
Final particle images (no.)	477,936
Map resolution (Å)	2.3
FSC threshold	0.143
**Model refinement and validation**	
Initial model used	L: Phyre2 prediction; P: PDB 4N5B
R.m.s. deviations	
Bond lengths (Å)	0.006
Bond angles (˚)	0.81
Validation	
Clashscore	13
Favored (%)	96.3
Allowed (%)	3.6
Outliers (%)	0.1

Table S2. GenBank accession number for L sequences, related to Figures 3, S2, and Data S3.

Abbreviation	Name	GenBank accession number
NiV (B)	Nipah virus, Bangladesh strain	AAY43917.1
NiV (M)	Nipah virus, Malaysia strain	NP_112028.1
HeV	Hendra virus	NP_047113.3
CedPV	Cedar virus	YP_009094087.1
GhV	Ghana virus	YP_009091839.1
MojV	Mojiang virus	YP_009094096.1
LayV	Langya virus	UUV47207.1
PIV3	Human parainfluenza virus 3	WCF97250.1
SeV	Sendai virus	YP_010790275.1
GSqRV	Giant squirrel respirovirus	YP_010806263.1
MeV	Measles virus	NP_056924.1
RDV	Rinderpest virus	P41357.1 (UniProtKB)
CDV	Canine distemper virus	NP_047207.1
BPV	Bat paramyxovirus	YP_010796325.1
BeV	Belerina virus	YP_010801294.1
PMPV1	Pohorje myodes paramyxovirus 1	YP_010085024.1
MMLPV1	Mountain Mabu Lophuromys virus 1	YP_009666847.1
RuV	Ruloma virus	YP_010801062.1
BaVV1	Bank vole virus 1	YP_010085015.1
TPMV	Tupaia paramyxovirus	NP_054697.1
ASPV	Atlantic salmon paramyxovirus	YP_009094152.1
FLDV	Fer-de-Lance paramyxovirus	NP_899661.1
APV	Avian paramyxovirus UP0216	YP_009508504.1
PPMV1	Pigeon paramyxovirus 1	QBZ96492.1
NDV	Newcastle disease virus	YP_009513199.1
APV17	Avian paramyxovirus 17	YP_010796382.1
HPIV2	Human parainfluenza virus 2	NP_598406.1
MuV	Mumps virus	NP_054714.1
PIV5	Parainfluenza virus 5	QWL54719.1
WTSPV	Wenling tonguesole paramyxovirus	YP_010790495.1
WHPV	Wenling hoplichthys paramyxovirus	YP_010790504.1
GPV	Gerbil paramyxovirus	UWK09075.1
FPaV	Feline paramyxovirus 163	YP_010799262.1
EBOV	Zaire Ebola virus	AQA27307.1
VSV	Vesicular stomatitis virus	NP_041716.1
RABV	Rabies virus SAD-B19	P16289.1
HMPV	Human metapneumovirus	YP_009513273.1
AMPV	Avian metapneumovirus	Q2Y2L8.2 (UniprotKB)
RSV	Respiratory syncytial virus	NP_044598.1
MPV	Murine pneumonia virus	YP_010774683.1
