# Supplementary material for: Structural and functional analysis of the Nipah virus polymerase complex
Source: Cell. 2025 Feb 6;188(3):688–703.e18. doi: 10.1016/j.cell.2024.12.021 (PMC11813165; doi:10.1016/j.cell.2024.12.021)
Supplement: Data S1. LC-MS/MS sequencing of purified NiV L and P, related to Figure 1 [file mmc2.pdf]

Data S1. LC-MS/MS sequencing of purified NiV L and P, related to Figure 1.

NiV L

MADELSISDI IYPECHLDSP IVSGKLISAI EYAQLKHNQP SDDKRLSENI RLNLHGKRKS LYILRQSKQG NYIRNNVKNL KEFMHIAYPE CNNTLFSITS QGMTSKLDNI MRKSFKAYNI

ISKKVIGMLQ NITRNLITQD RRDEIINIHE CRRLGDLGKN MSQSKWYECF LFWFTIKTEM RAVIKNSQKP KFRSDSCIIH MRDKSTEIIL NPNLICIFKS DKTGKKCYYL TPEMVLMYCD

VLEGRMMMET TIKSDIKYQP LISRSNALWG LIDPLFPVMG NRIYNIVSMI EPLVLALLQL KDEARILRGA FLHHCIKEMH QELSECGFTD QKIRSMFIDD LLSILNIDNI HLLAEFFSFF

RTFGHPILEA KVAAEKVREH MLADKVLEYA PIMKAHAIFC GTIINGYRDR HGGAWPPLYL PAHASKHIIR LKNSGESLTI DDCVKNWESF CGIQFDCFME LKLDSDLSMY MKDKALSPIK

DEWDSVYPRE VLSYTPPKST EPRRLVDVFV NDENFDPYNM LEYVLSGAYL EDDQFNVSYS LKEKETKQAG RLFAKMTYKM RACQVIAEAL IASGVGKYFK ENGMVKDEHE LLKTLFQLSI

SSVPRGNSQG NDPQSINNIE KDFQCFKGVT TSVKDKKNDP FYKVKSALNN PCQADGVYHN MSPNIRNRYK CSNTSKSFLD YHTEFNPHNH YKSDNTEAAV LSKYEDNTGT KFDTVSAFLT

TDLKKFCLNW RYESMAIFAE RLDEIYGLPG FFNWMHKRLE RSVIYVADPN CPPNIDKHME LEETPEDDIF IHYPKGGIEG YSQKTWTIAT IPFLFLSAYE TNTRIAAIVQ GDNESIAITQ

KVHPNLPYKV KKEICAKQAQ LYFERLRMNL RALGHNLKAT ETIISTHLFV YSKKIHYDGA VLSQALKSMS RCCFWSETLV DETRSACSNI STTIAKAIEN GLSRNVGYCI NILKVIQQLL

ISTEFSINET LTLDVTSPIS NNLDWLITAA LIPAPIGGFN YLNLSRIFVR NIGDPVTASL ADLKRMIDHS IMTESVLQKV MNQEPGDASF LDWASDPYSG NLPDSQSITK TIKNITARTI

LRNSPNPMLK GLFHDKSFDE DLELASFLMD RRVILPRAAH EILDNSLTGA REEIAGLLDT TKGLIRSGLR KSGIQPRLVS RLSHHDYNQF LILNKLLSNR KQNDLISSNT CSVDLARALR

SHMWRELALG RVIYGLEVPD ALEAMVGRYI TGSLECQICE QGNTMYGWFF VPRDSQLDQV DKEHSSIRVP YVGSSTDERS DIKLGNVKRP TKALRSAIRI ATVYTWAYGD NEECWYEAWY

LASQRVNIDL DVLKAITPVS TSNNLSHRLR DKSTQFKFAG SVLNRVSRYV NISNDNLDFR IEGEKVDTNL IYQQAMLLGL SVLEGKFRLR LETDDYNGIY HLHVKDNCCV KEVADVGQVD

AELPIPEYTE VDNNHLIYDP DPVSEIDCSR LSNQESKSRE LDFPLWSTEE LHDALAKTVA QTVLEIITKA DKDVLKQHLA IDSDDNINSL ITEFLIVDPE LFALYLGQSI AIKWAFEIHH

RRPRGRHTMV DLLSDLISNT SKHTYKVLSN ALSHPRVFKR FVNCGLLLPT QGPYLHQQDF EKLSQNLLVT SYMIYLMNWC DFKKYPFLIA EQDETVINLR EDIITSKHLC VIIDLYANHH

KPPWIIDLNP QEKICVLRDF ISKSRHMDTS SRSWNTSDLD FVIFYASLTY LRRGIIKQLR IRQVTEVVDT TTMLRDNIIV ENPPIKTGVL DIRGCIIYNL EEILSMNTKS SSKKIFNLNS

KPSVENHKYR RIGLNSSSCY KALNLSPLIQ RYLPSGAQRL FIGEGSGSMM LLYQSTLGQS ISFYNSGIDG DYIPGQRELK LFPSEYSIAE EDPSLAGKLK GLVVPLFNGR PETTWIGNLD

SYEYIINRTA GRSIGLVHSD MESGIDKNVE EILVEHSHLI SIAINVMMED GLLVSKIAYT PGFPISRLFN MYRSYFGLVL VCFPVYSNPD STEVYLLCLQ KTVKTIIPPQ RVLEHSDLHD

EVNDQGITSV IFKIKNSQSK QFHEDLKKYY HIDQPFFVPT KITSDEQVLL QAGLKLNGPE ILKSEISYDI GSDINTLRDT IIIMLNEAMN YFDDNRSPSH HLEPYPVLER TRIKTIMNRV

TKKVIVYSLI KFKDTKSSEL YHIKNNIRRK VLILDFRSKL MTKTLPKGMQ ERREKSGFKE VWIVDLSNRE VKIWWKIIGY ISIIGGSGGE NLYFQSGGGS AWSHPQFEKG GSGGSGGSSA

WSHPQFEKGG SGGSAWSHPQ FEK

NiV P

MHHHHHHSGG ENLYFQSMDK LELVNDGLNI IDFIQKNQKE IQKTYGRSSI QQPSIKDRTK AWEDFLQCTS GESEQVEGGM SKDDGGVERR SLEDLSSTSP TDGTIGKRVS NTRDWAEGSD

DIQLDPVVTD VVYHDHGGEC TGYGFTSSPE RGWSDHSSGA NNGDVCLVSD AKVLSYAPEI AVSKEDRETD LVHLEDKLSA TGLNPTAIPF TPKNLSVPAK DSPVIAEHYY GLGVREQNVD

PQTNRNVNLD SIKLYTSDDE EADQLEFEDE FAGSSSEVIV GISPEEEEPS SAGRKPIESV GHIIEGQSTR DSLQIKGNKP ADAPGAGPKD SAVKEKSPQK RLPMLAEEFE CSGSEDPIIQ

ELLKENSFIN SQQGKDAQPL YYRGIEGSRS PDKTEITSDA VQTANKQRPG TPMPKSRGIP IKKGTDEKYP SAGTENVPGS KSGATRHVRG SPPYQEGKSV NAENVQLNVP TVVKETDKSE

ANPADDNDSL DDKYIMPSDD FSNTFFPHDT DRLNYHADHL GDYDLETLCE ESVLMGVINS IKLINLDMRL NHIEEQVKEI PKIINKLESI DRVLAKTNTA LSTIEGHLVS MMIMIPGKGK

GERKGKSNPE LKPVIGRDVL EQQSLFSFDN VKNFRDGSLT NEPYGAAVQL RGDLILPELN FEETNASQFV PMADDSSRDV VKTLIRTHIK DRELRSELIG YLNRAENDEE IQEIANTVND

IIDGNI
