# Supplementary figures and images for: Structural and functional analysis of the Nipah virus polymerase complex
Source: Cell. 2025 Feb 6;188(3):688–703.e18. doi: 10.1016/j.cell.2024.12.021 (PMC11813165; doi:10.1016/j.cell.2024.12.021)

Data S2. Comparison of nsNSV cryo-EM structures, related to Figure 1.

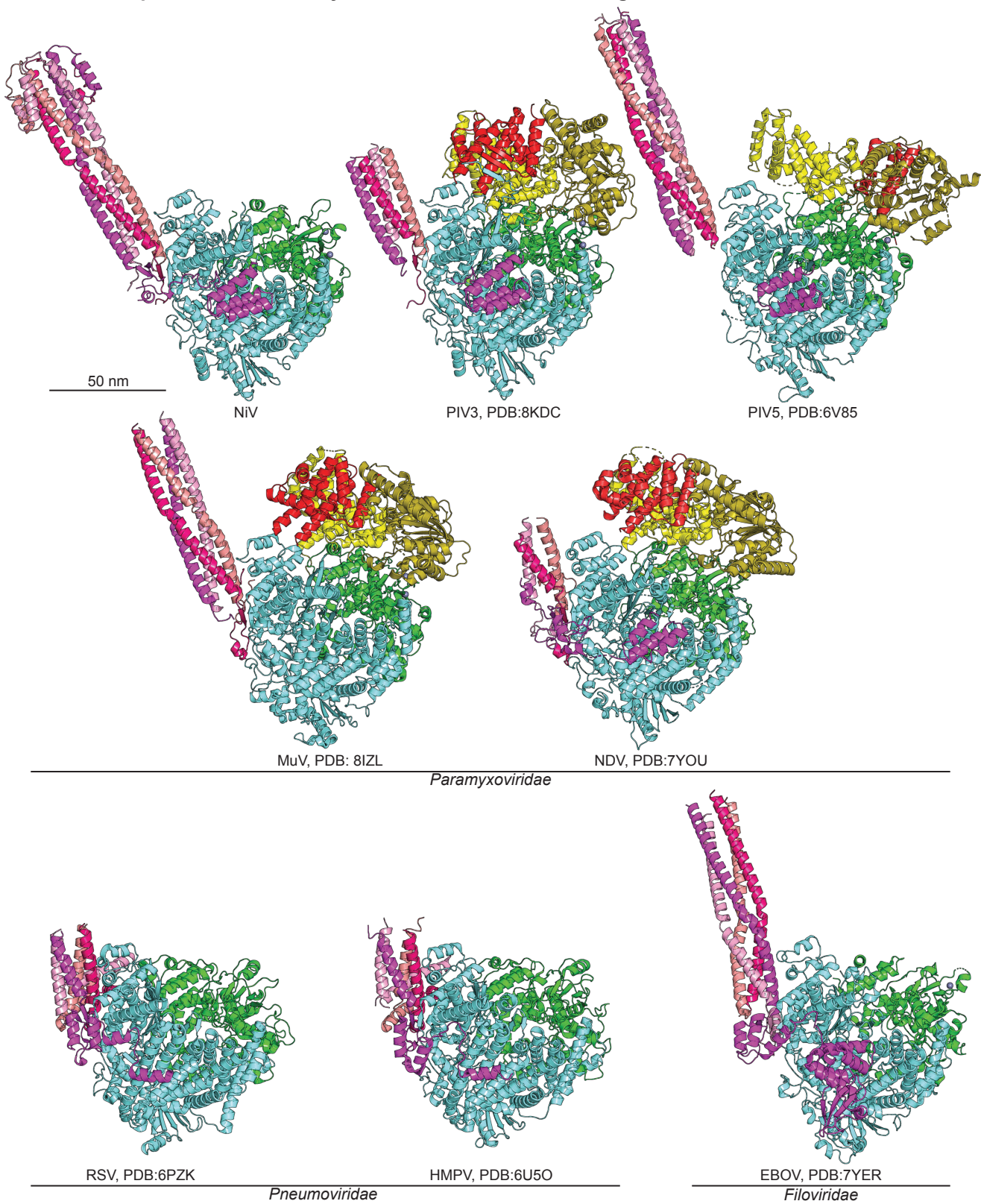

Supplement: Data S2. Comparison of nsNSV cryo-EM structures, related to Figure 1 [file mmc3.pdf]
