# Supplementary material for: Structural and functional analysis of the Nipah virus polymerase complex
Source: Cell. 2025 Feb 6;188(3):688–703.e18. doi: 10.1016/j.cell.2024.12.021 (PMC11813165; doi:10.1016/j.cell.2024.12.021)
Supplement: Data S3. Amino acid sequence alignment of nsNSV L proteins, related to Figure 2 [file mmc4.pdf]

Data S3. Amino acid sequence alignment of nsNSV L proteins, related to Figure 2.

```
NiV(B)    1  .........................MADELSISDIIYPECHLDSPIVSGKLISAIEYA.Q
NiV(M)    1  .........................MADELSISDIIYPECHLDSPIVSGKLISAIEYA.Q
HeV       1  .........................MAHELSISDIIYPECHLDSPIVSGKLISAIEYA.Q
MojV      1  .............M...........NFSDVSVSDILYPECHLNSPIVTGKIVQVVRFS.D
LayV      1  .............M...........NFSDVSISDILYPECHLNSPIVTGKIVQAVRFS.E
GhV       1  ............ME...........LEGNVSLTDILYPECHLDSPLVTGKLIQALEYA.N
PIV3      1  .MD.......T..............ESNNGTVSDILYPECHLNSPIVKGKIAQLHTIM.S
SeV       1  .MD.......G..............QESSQNPSDILYPECHLNSPIVRGKIAQLHVLL.D
GSqRV     1  .MD.......N..............QNSAQNPSDILYPECHLNSPIVRGKIAQLHVLM.D
MeV       1  ..........................MDSLSVNQILYPEVHLDSPIVTNKIVAILEYA.R
RDV       1  ..........................MDSLSVNQILYPEVHLDSPIVTNKLVAILEYA.R
CDV       1  ..........................MDSVSVNQILYPEVHLDSPIVTNKLVSILEYA.R
BPV       1  ............................MENLTDIIYPESHLDSPLVAGKLVELLMYS.G
BeV       1  .........................MDQTPKVSDILYPECHLDSPIVTGKLVELLNYT.G
PMPV1     1  ...........................MSAQISDILYPECHLDSPIVTGKLISLLIKG.N
MMLPV1    1  ..........................MAGTSLSDILYPECHLNSPIVTGKLVELILVT.G
RuV       1  .........................MSLSYSLSDVLYPEAHLNSPIVAGKLITMVLYA.D
BaVV      1  ...........................MDLLDETILYPESHLNSPLVCQKLVTFIEYS.C
ASPV      1  .MD.......ADQP...........VQSNKDCSSILYPECHLNSPIILGKIVQCHVAM.G
FLDV      1  .............................MDPKDILYPECHLDSPIVLGKVIRLHYLL.D
APV       1  ...........MAE...........PTGVRAEHQIILPESHLSSPLVKHKLLYYWKLT.G
PPMV1     1  ...........MAS...........TGPERAEHQIILPESHLSSPLVKHKLLYYWKLT.G
NDV       1  ...........MAG...........SGPERAEHQIILPESHLSSPLVKHKLLYYWKLT.G
APV17     1  ...........MAE...........LGQGRPDHQVILPEAHLSSPIVRHKLLYLWRLT.G
HPIV2     1  ............................MAASSEILLPEVHLNSPIVKHKLIYYLLLG.H
MuV       1  ............................MAGLNEILLPEVHLNSPIVRYKLFYYILHG.Q
PIV5      1  ............................MAGSREILLPEVHLNSPIVKHKLYYYILLG.N
WTSPV     1  MMDEFTLEN.PFEK...........DSNSCTANTILFPEVHLNSPIVLNQLRLICHIA.E
WHPV      1  ...MYF....EEDA...........DEGGVPGHDILLPEIRLGSPICLGKLALIQEIS.G
GPV       1  ...........MNS...........QYDNISVNDILYPECHLDSPIVTGKLYTLLEKG.G
FPaV      1  .........................MADFLHLSDILYPECHLDSPIVTGKLVSILIYS.K
HMPV      1  .MD..........P...........L..NESTVNVYLPDSYLKGVISFSETNAI.GSC.L
AMPV      1  .MD..........P...........L..NEGVVNVYLTDSYLKGVISFSETNAI.GSC.L
RSV       1  .MD..........P...........II.NGNSANVYLTDSYLKGVISFSECNAL.GSY.I
MPV       1  .MD..........P...........I..DEQEVNVYLPDSYLKGVISFSETNAL.GSC.I
EBOV      1  .............................MATQHTQYPDARLSSPIVLDQCDLVTRAC.G
VSV       1  .MEVHDFETDEFNDFNEDDYATREFLNPDERMTYLNHADYNLNSPLISDDIDNLIRKFNS
RABV      1  MLDPGEVYDDPIDPIELE....AEPRGTPIVPNILRNSDYNLNSPLIEDPARLMLEWLKT
```

```
NiV(B)   35  LKHNQPSDDKRLSENIRLNLHGKR...KSLYILRQSKQGNYI.RNNVKNL..........
NiV(M)   35  LRHNQPSDDKRLSENIRLNLHGKR...KSLYILRQSKQGDYI.RNNIKNL..........
HeV      35  LRHNQPNGDKRLTENIKINLQGKR...RSVYISRQSRLGNYI.RDNIKNL..........
MojV     36  IPCNQILIDPTLNEIIDIKLKSNK...PGLIRRRQKEYGILL.KQVAGDI..........
LayV     36  IPCNQILIDPTLNDVIDIKLRSNK...QGLIRRRQKEYGLLL.KQVAGDI..........
GhV      37  LTHNQYLKDKTLLNNIQLNKSKGK...RSRYTIAQEQFGNFI.KDKINNL..........
PIV3     38  LPQPYDMDDDSILVITRQKIKLNK...LDKRQRSIRRLKLIL.IEKVSDL..........
SeV      38  VNQPYRLKDDSIINITKHKIRNGG...LSPRQIKIRSLGKAL.QRTIKDL..........
GSqRV    38  VNQPYILQDDSIINITKHKIRNGG...LSPRQIKIRSLRRIL.QKGIRNF..........
MeV      34  VPHAYSLEDPTLCQNIKHRLKNGF...SNQMIINNVEVGNVI.KSKLRSY..........
RDV      34  IPHKYVLEDPTLCKNIKHRLDSGF...SNQMVINNVEIGNVV.QSKLKGY..........
CDV      34  IRHNYQLLDTTLVRNIKERISEGF...SNQMIINCIEIGSII.NQTLLSY..........
BPV      32  LPHNQPLKDDVLINNIKSNISKNK...KSPLVNTQLEFRKHL.LENFIPI..........
BeV      35  LPFTDTLKDKTLIRNLKINEDNCR...GNNIQYTQRKYGKII.VDLVGNP..........
PMPV1    33  FPHREDLKDPTLLRNLDANIGDIH...RLQITIDQQKLRDAL.ITKYHDL..........
MMLPV1   34  LPNNQHLDDQTIITNIRKNLSLGY...HNSLILQQRQFNHIL.TKKYPNL..........
RuV      35  LPCNQSLDDKQILLNIHRNKQRGV...SNSVIRTQKEIGLEI.RKYYPNF..........
BaVV     33  LPHNQELSDKTIIPTIRSKISNIG...HPHHLRILYHSGRIV.HPWLTTQ..........
ASPV     41  VHQPYMMEDDTIITNTRKNLRTRR...ETGRLCSLRKIGGIL.KGIVPSL..........
FLDV     31  LTVEYDIKDSSILRNLDYNAKSGR...LSNRDRELVKHKDIL.RKEGFVR..........
APV      38  LPLPEECEFDPLILGRQWRKILET...GAPDTERMVKLGRLA.HQNL.............
PPMV1    38  LPLPDECDFDHLIISRQWKKILES...ATPDIERMIKLGRAV.HQTL.............
NDV      38  LPLPDECDFDHLIISRQWKRILES...ATPDTERMIKLGRAV.HQTL.............
APV17    38  LPLPQVAELDPLLLSRPWSRIAQS...ASPEVARMKDLGVRV.HTTI.............
HPIV2    32  FPHDLDISEISPLHNNDWDQIARE...ESNLAERLGVAKSEL.IKRVPAF..........
MuV      32  LPNDLEPDDLGPLANQNWKAIRAE...ESQVHARLKQIRVEL.IARIPSL..........
PIV5     32  LPNEIDIDDLGPLHNQNWNQIAHE...ESNLAQRLVNVRNFL.ITHISDL..........
WTSPV    48  IPAGFDLRDTTILHNHDSADEQQRKKRNGPLYQSARHIKDVA.HRILKTS..........
WHPV     42  IDFAISLRDDAIIKNYRGKSAISKTGERHVVTDCAKEIRRSL.MSRFPKI..........
GPV      38  FPHNETLSDKVLEHNLAANRSITT...KQQTSIDQRRLYNTL.LSKYPNT..........
FPaV     35  LPYNQELKDKTLIDNCLQNLSKGI...KNNMINQQNYFREQL.LQHYGDI..........
HMPV     35  LKRPYLKNDNTAKVAIENPVIEHV...........RLKNAV..NSKMK............
AMPV     35  LGKHYLKKDNTSKVAIESPVVEHI...........RLRNAF..QTRIK............
RSV      36  FNGPYLKNDYTNLISRQNPLIEHI...........NLKKLNITQSLMSKY..........
MPV      35  IGRPFLKDDFTATTSIRNPLIEHK...........RIRDTKLVKNIVS...........
EBOV     31  LYSSYSLNPQ..LRNCKLPKHIYR...........LKYDVTV.TKFLSDVPVATLPIDFI
VSV      60  LPIPSMWD.S.....KNWDGVLEM...LTSCQANP..ISTSQMHKWMG............
RABV     57  GNRPYRMTLTD.NCSRSFRVLKDY...FKKVDLGSLKVGGMAAQSMIS............
```

```
                          .         .               .            .
NiV(B)       81  .........KEFMHIAYPECNNTLFSITS...QGM...TSKLDNIMRKS...FKAYNII.
NiV(M)       81  .........KEFMHIAYPECNNILFSITS...QGM...TSKLDNIMKKS...FKAYNII.
HeV          81  .........KEFLHVSYPECNKSLFSLKS...PGM...TSKLSNIMKKS...FKAYNIV.
MojV         82  .........TSYQHIPYPECNTRLFYISD...SSL...VCCLSEIMTHA...NQCYLKI.
LayV         82  .........TSYQHIPYPECNTRLFYIHD...PSL...VDCLTEIMTHA...NQCYLKI.
GhV          83  .........SQFYHVPYPECNYNLFRLSS...DRI...VARLSDVLKVP...YDCYLSN.
PIV3         84  .........GKYTFIRYPEMSKEMFKLHI...PGI...NSKVTELLLKA...DRTYSQM.
SeV          84  .........DRYTFEPYPTYSQELLRLDI...PEI...CDKIRSVFAVS...DRLTREL.
GSqRV        84  .........DRYTFEPYPTYSRELMRLDI...PEI...CDKIRAVFSIS...DRLMREL.
MeV          80  .........PAHSHIPYPNCNQDLFNIED...KES...TRKIRELLKKG...NSLYSKV.
RDV          80  .........PNHEHIPYPECNQELFIARD...KKA...TEKIRSLFKKG...TTLYTKI.
CDV          80  .........PKHNHVIYPNCNKLLFHAQD...RVI...SLRLRNIFKRG...NSIYSKI.
BPV          78  .........DKLNHIPYPEGNKFLFELTD...SNI...TKKIQLLMKLA...NSGYSKI.
BeV          81  .........SKLRHVPYPSGNLMLFNMRD...DDV...TLEISSHLRYA...SLCYKKI.
PMPV1        79  .........SSLSPVPYPYGNNYLFKIRD...NEY...TKDISTILTYA...NTCYKKI.
MMLPV1       80  .........RVLRHIPYPNGNYFLFNITN...NTI...ISELKSLLSYA...HSCYRKI.
RuV          81  .........KRLKPIPYPNGNKHLFSLTD...NKV...ISELKTILNYG...HKCYNKI.
BaVV         79  .........GNYIHIPYPECNKTLFRYVD...HKL...TRKLDQVLSHA...NAAYNSV.
ASPV         87  .........PNYRFEAYPYNTKEMLRLDL...KER...CDELDNLLGRA...DSCFNYN.
FLDV         77  .........SGYRGIDYPRGNKELFRLVI...PNL...TKPLECLLKQS...NKVFDRI.
APV          81  ......NHNKPIRGLLHPRTLTMLTTIEI...PDS...SKKFRTIEKKI...QIHNKVF.
PPMV1        81  ......NHNSKITGVLHPRCLEELSSIEV...PDS...TNKFRKIEKKI...QIHNTRY.
NDV          81  ......NHNSKITGVLHPRCLEELANIEV...PDS...TNKFRKIEKKI...QIHNTRY.
APV17        81  ......AHDGNMSPIMHPATLPWLVRLRI...PIA...IERFRMVEKKV...QGLLSSR.
HPIV2        78  ...RATRWRSHAAVLIWPSCIPFLVKFLP...HSK...LQPVEQWYKLI...NASCNTI.
MuV          78  ...RWTRSQREIAILIWPRILPILQAYDL...RQS...MQLPTVWEKLT...QSTVNLI.
PIV5         78  ...RKGHWQEYVNVILWPRILPLIPDFKI...NDQ...LPLLKNWDKLV...KESCSVI.
WTSPV        97  ............QCIDAYKIHKDLIRIKD...TVI...GHDAKILWKQAVHHMDCFQKN.
WHPV         91  ...R......SMQPVD...PHKFILQSQ..............DRWDRKVA..QVSEENIE
GPV          84  .........HKLNPVPYPYANKYLFRIID...NEY...IDDITSVLSLA...STCYKKI.
FPaV         81  .........SKLYNVPYPYGNDYLFHIID...RKF...SESLRQVLSYS...HLCYRKV.
HMPV         70  ........ISDYKIVEPVNMQHEIMKNVH......SCELTLLKQFLTRS...........
AMPV         70  ........EKNLRVVEPVNMQSEVMRNSY......TCELNLLKQLITRS...........
RSV          75  .......HKGEIKIEEPTYFQSLLMTYKSMTSLEQITTTNLLKKIIRRA...........
MPV          72  ........NPQYRLVEPLQMQHELLSVLS...PNFILHTANLRKIIQRS...........
EBOV         77  VPILLKALSGNGFCPVEPRCQQFLDEII..................KYTMQ.DALFLK..
VSV          97  .....................SWLMSDNHDASQG....YSFLHEVDKEA.......EI..
RABV        101  .....................LWLYGAHSESNRS....RRCITDLAHFY.......SK..
```

```
                         .          .
NiV(B)      122  .........SKKVIGMLQ.NITRNLITQDRRD..E....IIN..................
NiV(M)      122  .........SKKVIGMLQ.NITRNLITQDRRD..E....IIN..................
HeV         122  .........SRKIIEMLQ.NITRNLITQDQKD..E....VLG..................
MojV        123  .........SSKILDLLD.KTECNLTGIGSRD..D....DET..................
LayV        123  .........SSRILDLLD.KTECSLTGIGSRD..D....DET..................
GhV         124  .........IEKLLGMLT.AVDRKLGGQPLCK..D....VTG..................
PIV3        125  .........TDGLRDLWI.NVLSKLASKNDGS..N....YDL..................
SeV         125  .........SSGFQDLWL.NIFKQLGNIEGRE..G....YDP..................
GSqRV       125  .........SGGFQDLWT.NIFEQLGNLSGKE..G....YDP..................
MeV         121  .........SDKVFQCLR.DTNSRLGLGSELR............................
RDV         121  .........SDKVCTCLE.TANSRLGLGADLG............................
CDV         121  .........TDGVKKCLN.DINLNIGLGGALD............................
BPV         119  .........SQRLVLLKN.HVESGLGLRKKYHKRK....HDE..................
BeV         122  .........SRKIIQLRR.QTEYTLGRSKGIP..S....PEV..................
PMPV1       120  .........SGKIIRLED.LVQSKLGSYNQNS..N....YTT..................
MMLPV1      121  .........SDRLVHLVN.HVESTLGENHTRD..K....DVD..................
RuV         122  .........SGRLLKLKK.SLLDQLGTTDKVL..P....VEE..................
BaVV        120  .........AERIYDCLK.SIRLKLGSTLSKE..D....IMN..................
ASPV        128  .........KEKVSAMLK.SCLVKGVGLPSQD..I....PGL..................
FLDV        118  .........ESGLRDTFL.ASANKISGKGKEV..F....TEQ..................
APV         125  .........GGLFNTLCD.QVEQKLLGRHSKQI......ISR..................
PPMV1       125  .........GDLFAKLCT.HVEKKLLGSSRSSK......VPR..................
NDV         125  .........GDLFTKLCT.HVEKKLLGSSRSNN......VPR..................
APV17       125  .........RDNFNRLIE.QVNTDLFGTLPDYD......LCP..................
HPIV2       125  .........SDSIDRCME.NISIKLTGKNNLFSRSRGTAGAG..................
MuV         125  .........SDGLERVVL.HISNQLTGKPNLFTRSRAGQDVK..................
PIV5        125  .........NAGTSQCIQ.NLSYGLTGRGNLFTRSRELSGDR..................
WTSPV       138  .........DKKFIDELF.HNLDHVEGRS...............................
WHPV        123  TAVREMISNMDRYTDWIK.TISEVWDCK............ST..................
GPV         125  .........SHRIVKLKN.LTLNQLGIESINN..T....KED..................
FPaV        122  .........SNYFIKLMT.LVESKLGINSNPN..D....EEY..................
HMPV        105  ..........KNISTLKLNMICDWLQLKSTSDD...........................
AMPV        105  ..........KDISSLKLDMICDWLQLKSTSEN...........................
RSV         117  ..........IEISDVKVYAILNKLGLKEKDKIKSNNGQDEDNSVITTIIKDDILLAVKD
MPV         110  ..........VDITDKKLNPILHILNLNSPNQE...........................
EBOV        116  ...................YYLKNVGAQEDCV............................
VSV         123  .........TFDVVE....TFIRGWGNKP...............................
RABV        127  .........SSPIEK....LLNLTLGNRGLRI............................
```

```
NiV(B)   148  ........................IHECRRLGDLGKNMSQSKWYEC...........FLF
NiV(M)   148  ........................IHECRRLGDLGKNMSQSKWYEC...........FLF
HeV      148  ........................IYEQDRLSNIGKYMSQSQWYEC...........FLF
MojV     149  ........................RIIYNNVETLPKIMAQSKWYKP...........FLF
LayV     149  ........................RIIYNNIETLPKIMAQSRWYKS...........FLF
GhV      150  ........................SAPDDKLVHLPDTMKQSDWYMP...........FLF
PIV3     151  ........................NEEINNISKVHTTYKSDKWYNP...........FKT
SeV      151  ........................LQDIGTIPEITDKYSRNRWYRP...........FLT
GSqRV    151  ........................LRDINLIPDIVGRYNRNKWYRS...........FLT
MeV      143  ........................EDIKEKIINLGVYMHSSQWFEP...........FLF
RDV      143  ........................IGIKEKILSLSLYMQSSQWYEP...........FLF
CDV      143  ........................KTIGTKIDEAGIIMQSSQWFEP...........FLL
BPV      147  ........................IYTENAMRSLHHKMEGSKWYQP...........FLF
BeV      148  ........................ETEEHMMSCVSEVMQASKWYHP...........FMF
PMPV1    146  ........................NDERAKIRRLPNIMEGSKWYNP...........FLF
MMLPV1   147  ........................DDRKLQIVNLPNILESSKWYKP...........FLF
RuV      148  ........................ELINKKIVSLPNIMESSNWYNP...........FLF
BaVV     146  ........................NDDSTKILDLPEIMISSQWYGP...........FLF
ASPV     154  ........................SDDCAAGESLPAAARRSRWFKS...........FKS
FLDV     144  ........................NLTESGMAGVSRSIHNNGWNQP...........FMT
APV      151  ........................SPEYLRLREDPAFWFHSAWSSA...........KFA
PPMV1    151  ........................SEEFSSIRTDPAFWFHSRWSRA...........KFA
NDV      151  ........................SEEFSSIRTDPAFWFHSKWSRA...........KFA
APV17    151  ........................SPEFQELLSGTGFWFRSDYSES...........KFS
HPIV2    157  ........................KNSKITLNDIQSIWESNKWQPN...........VSL
MuV      157  ........................DYSIPSTRELSQIWFNNEWSGS...........VKT
PIV5     157  ..........................RDIDLKTVVAAWHDSDWKRI...........SDF
WTSPV    157  ..........................TRHDWNEISPLFKSSKYRDS...........VNV
WHPV     152  ........................EDPKADMIEAMGRYATSMYRGP...........FSC
GPV      151  ........................IYENNNIIRLSSIMEGSNWYKP...........FLF
FPaV     148  ........................MSRLDSMEFLSQIIEGSKYYEP...........FLF
HMPV     128  ................TS......ILSFIDVEFI......PSW..............VSN
AMPV     128  ................PS......VLKFVDVRCI......PDW..............VST
RSV      167  NQSHLKAVKNHSTKQKDTIKTTLLKKLMCSMQHP......PSW..............LIH
MPV      133  ................GKVSERLTRLIKKHLSHI......PNW..............VSS
EBOV     129  ..........................DDHFQEKILSSIQGNEFLHQ...........MFF
VSV      139  ..................................IEYIKKERWTDSFK.ILAYLCQKFLD
RABV     146  .............................PPEGVLSCLERVDYDNAFGRYLANTYSSYLF
```

```
NiV(B)   173  ............WFTIKTEMRAVIKNSQKPKF...RSDSCIIHMRDKSTEIILNPNLI..
NiV(M)   173  ............WFTIKTEMRAVIKNSQKPKF...RSDSCIIHMRDKSTEIILNPNLI..
HeV      173  ............WFTIKTEMRAVIKNSQKPKF...RSDSCIIHMKDNNMEIVMNPNLV..
MojV     174  ............WFTVKTEMRNNIKENKKYSR...HNRPHITQLSGKLIYVQLNMNLI..
LayV     174  ............WFTVKTEMRNNIKENKKYSK...HNRPHITQLSGKQLYVQINMNLI..
GhV      175  ............WFTVKTELRLLQKESQKVKN...RGSSNIVQFGDNHLTVVMNPSLV..
PIV3     176  ............WFTIKYDMRRLQKARNEVTF...NMGKDYNLLEDQKNLLLIHPELV..
SeV      176  ............WFSIKYDMRWMQKTRPGGPL...DTSNSHNLLECKSYTLVTYGDLI..
GSqRV    176  ............WFSIKYDMRWLQKTRTGNHL...DASNSHNLLECRTYTLVTYSDLV..
MeV      168  ............WFTVKTEMRSVIKSQTHTCH...RRRHTPVFFTGSSVELLISRDLV..
RDV      168  ............WFTIKTEMRSTIKSSVHTSH...RRRYVPSFFSGDSFEILISRDLV..
CDV      168  ............WFTIKTEMRSVIKSSTHNCR...KRRQNPVFVKGESLNVLVSRDLV..
BPV      172  ............WFSIKTDMRQVIKQHVNSRR...KDQINIIDYDLPRLYIQMNRNLV..
BeV      173  ............WFTLKYEMRKILKNTAPGNQ...AVHQNVKIIDTDRHIIVINRNLC..
PMPV1    171  ............WFKCKYMMRKMIKRGVKLKY...NNYNTEQVFDLYDHFIAINKHLT..
MMLPV1   172  ............WFSIKKEMRECIKISTSNIN...RGVYPNKVFDVDNLYIQLNRNIL..
RuV      173  ............WFTIKTYMRKIIKKSGKKFT...SQSDRIKTIISNSLIIVINRNLA..
BaVV     171  ............WFTIKTEMRSCIKDYTNMRG...RKQEKPMVLKSADSLIVINRLLV..
ASPV     179  ............WFTIKREMRILQKACNKGAR...MNKEQTKWLPGNPYDVFITAEVV..
FLDV     169  ............WFTIKKEMRKLKKDYRKNAK...STGMTRLASHHERYSLYIHSDFV..
APV      176  ............WLHIKQLQRHLTVCARS..R...MGPGNPITLIHNHGQVFVTPELV..
PPMV1    176  ............WLHIKQIQRHLIVAART..R...SAVSKLVTLTHKLGQVFVTPELV..
NDV      176  ............WLHIKQVQRHLIVAART..R...SAVNKLVTLSHKIGHVFVTPELV..
APV17    176  ............WLHLKQIQRHLILSART..R...STQVLYVCVRSSVGHVHVTPDLV..
HPIV2    182  ............WLTIKYQMRQLIMHQSS..R...QPTDLVHIVDTRSGLIVITPELV..
MuV      182  ............WLMIKYRMRQLITNQKT..G...ELTDLVTIVDTRSTLCIIAPELV..
PIV5     180  ............WIMIKFQMRQLIVRQTD..H...NDPDLITYIENREGIIIITPELV..
WTSPV    180  ............WYKIRHVMREVQNSRGSAGK...NGYNLVKIIKGGTWNCYINGAWV..
WHPV     177  ............WNSVRKQFRAIIHRYESKKA...SSATKAKVLDFDRWTLIITCTAM..
GPV      176  ............WFKVKYTMRKLIKRGQKLKY...KSYNTEVIADTNVHYIGINKHLV..
FPaV     173  ............WFTLKTYMRNIIKMSVSPTH...KNKYCIDYFDTDNHYIQINRNLV..
HMPV     146  ............WFSNWYNLNKLILEFRKEEV.IRTGSILCRSLGK..LVFVVSSYGC..
AMPV     146  ............WFSSWYNLNKLILEFRREEV.ACTGSIICKTIGS..IMFIISSFGC..
RSV      207  ............WFNLYTKLNNILTQYRSSEV.KNHGFILIDNHTLNGFQFILNQYGC..
MPV      157  ............WYNIWVNLNNLLQEYRSKEV.IDHNCVLTRQLSGSFIHVVMSQYGV..
EBOV     152  ............WYDLAILTRRGRLNRGNSRSTWFVHDDLIDILGYGDYVFWKIPISMLP
VSV      164  LHKLTLILNAVSEVELLNLARTFKGKVRR....SSHGTNICRIRVPSLGPTFISEGWA..
RABV     177  FHVITLYMNALDWDEEKTILALW.KDLTS....VDIGKDLVKFKDQIWGLLIVTKDFV..
```

```
NiV(B)    216  ....................CIFKSDKTGKKCYYLTPEMVLMYCDVLEGRMMMETTIK..
NiV(M)    216  ....................CIFKSDKTGKKCYYLTPEMVLMYCDVLEGRMMMETTVK..
HeV       216  ....................CIYKNDKDGKRCYYLTPEIVLMCCDVLEGRMMIETSIK..
MojV      217  ....................SIIEW..EKKCIYHLTPELVLMYCDVIEGRLMIDTVMK..
LayV      217  ....................SIIEW..DKKCIYHLTPELVLMYCDVLEGRLMIDTVMQ..
GhV       218  ....................TIWAQ..KSKQCYYLTPEHVLMFCDVLEGRLMIDVTVK..
PIV3      219  ....................LILDK..QNYNGYLITPELVLMYCDVIEGRWNISACAK..
SeV       219  ....................MILNK..LTLTGYILTPELVLMYCDVVEGRWNMSAAGH..
GSqRV     219  ....................MILNK..LTLTGYILTPELVLMYCDVVEGRWNMSSAGQ..
MeV       211  ....................AIISK..ESQHVYYLTFELVLMYCDVIEGRLMTETAMT..
RDV       211  ....................AIVHR..RLHHVYYLTFELVLMYCDIIEGRLMTETAMV..
CDV       211  ....................CIIDL..TSHIVYYLTFEMVLMYCDVIEGRLMTDTAMA..
BPV       215  ....................MIIDK..KTMRTHYLTNEMVLMFCDVTEGRLMIDLAMS..
BeV       216  ....................TIIQR..EGWATHYLTHELVLLYCDVAEGRLMIDSGMT..
PMPV1     214  ....................IIVDK..NTNNIYYLTFEMVLMFSDVLEGRLMIDTGME..
MMLPV1    215  ....................IIVNK..LTNEVHYLTFEMVLMFSDVLEGRLMIDFAMR..
RuV       216  ....................IIIDK..RDNTIHYLTFEMVLMFSDVVEGRLMIDLGMQ..
BaVV      214  ....................YIINI..KLREIYYLTFEMVLMMCDVVEGRLMCEIAMI..
ASPV      222  ....................VILSK..SGKIGHILTPELVLMYCDVVEGRWNMATAGS..
FLDV      212  ....................LISDH..VLEVYHLLTDDMVLGFCDVVEGRLMVSAVAS..
APV       217  ....................IITEN..SSNQFTCLTQEMVLSYSDMLEGRDMVSFVSS..
PPMV1     217  ....................IVTHT..DENKFTCLTQELVLMYADMMEGRDMVNIISS..
NDV       217  ....................IVTHT..DENKFTCLTQELVLMYADMMEGRDMVNIISS..
APV17     217  ....................VVTDL..ASQSFTALTQEMVLMYSDMLEGRDMVLATAR..
HPIV2     223  ....................ICFDR..LNSVLMYFTFEMTLMVSDMFEGRMNVTALCT..
MuV       223  ....................ALYSN..EHKALTYLTFEMVLMVTDMLEGRLNVSSLCT..
PIV5      221  ....................ALFNT..ENHTLTYMTFEIVLMVSDMYEGRHNILSLCT..
WTSPV     223  ....................CLHNK..SSNTHCFLSPNQVLMYNDLVEGRWLALLGAS..
WHPV      220  ....................VIVDR..RTLTYTIMDLNLVLMAVDLFESRWVIDMLT...
GPV       219  ....................YLINK..RSLNTYYFTFEMVLMFCDVCEGRLMIDVGMS..
FPaV      216  ....................MIIDK..RNRTSYYFTLEMILMFCDVCEGRLMIDLAMS..
HMPV      189  ....................IVKSN..KSKRVSFFTYNQLLTWKDVMLSRFNANFCIW..
AMPV      189  ....................VIKSN..KSKRISFMTYNQVLTWKDVMLSRFNANLCVW..
RSV       252  ....................IVYHK..ELKRITVTTYNQFLTWKNISLSRLNVCLITW..
MPV       202  ....................VIISK..KSKRYTMCTYNQFLTWKDLALSRFNANYVVW..
EBOV      200  LNTQGIPHAAMDWYQASVFKEAVQG..HTHIVSVSTADVLIMCKDLITCRFNTTLISK..
VSV       218  .....................Y....FKKLDILMDRNFLLMVKDVIIGRMQTVLSMVCR
RABV      230  .....................Y....SQSSNCLFDRNYTLMLKDLFLSRFNSLMVLLSP
```

```
NiV(B)    254  SDIKYQPLIS...........RSNALWGLIDPLFPVMGNRIYNIVSMIEPLVLALLQ.LK
NiV(M)    254  SDIKYQPLIS...........RSNALWGLIDPLFPVMGNRIYNIVSMIEPLVLALLQ.LK
HeV       254  SDIKYQSLIT...........RSNALWTFIDSLFPIMGNRIYNIVSMIEPLVLALLQ.LK
MojV      253  ADTRYHPLYN...........RAHQLWDLLDLMFEEIGNNMYNIISLIEPLTLGILQ.LQ
LayV      253  ADTRYHPLCE...........RAHQLWELLDLMFEELGNNMYNLISMIEPLTLGILQ.LQ
GhV       254  SDDRYKSLRT...........KSKELWELIDPFFPIIGNETYNIVSMLEPFTLATLQ.LK
PIV3      255  LDPKLQSMYQ...........KGNNLWEVIDKLFPIMGEKTFDVISLLEPLALSLIQ.TH
SeV       255  LDKKSIGITS...........KGEELWELVDSLFSSLGEEIYNVIALLEPLSLALIQ.LN
GSqRV     255  LDRRSFRITR...........KGEELWDLIDSLFSNLGEDVYNVIALLEPLSLALIQ.LN
MeV       247  IDARYAELLG...........RVRYMWKLIDGFFPALGNPTYQIVAMLEPLSLAYLQ.LR
RDV       247  IDPRYTELLE...........RVRYMWKLIDGFFPTLGNSTYQVVALLEPLSLAYLQ.LR
CDV       247  IDQRYSTLHV...........RIRYLWDLIDGFFPDLGNSTYQLVALLEPLSLAYLQ.LK
BPV       251  SDYRFKSFRS...........HGISLWQIIDELFEDLGNNTYNIVALIEPLTLGYLQ.LN
BeV       252  LDPRYRQLKV...........CGNLLWDFIDSQFAMLGNSTYDLVAMVEPLVLGYLQ.LN
PMPV1     250  ADDRFQLFKE...........KGHALWNFMDSLFPNLGNETYNIVAMIEPLVLGFLQ.LR
MMLPV1    251  SDLRYRQFKN...........RGYLLWEFIDSLFCDLGNETYNIVALIEPLVLGFLQ.LH
RuV       252  TDIRLKPIYR...........RGKYLWGLIDSLFVDLGNSTYNIVALIEPFVLGFLQ.LK
BaVV      250  CDSKYHVLHP...........NVKSLWILIDGLFPDLGNNTYNIVSLLEPLTLGFLQ.LK
ASPV      258  LDTKTSGITR...........RGEKLWSLIDTLFPRIGEKTFDVVALLEPLSLALIQ.LS
FLDV      248  MDPNYYVNIGI..........RGEQLWSLIDSLFILLGNKVYDVVGMLEPLALSYIQ.LR
APV       253  TATHLLPLKD...........KIQSLLLLIDALAMDMGNNVYEVISLMEGLAYGAVQ.LL
PPMV1     253  TATHLKNLSE...........KIDDILRLVDALSKDLGNQVYDVVALMEGFAYGAVQ.LL
NDV       253  TAAHLRNLSE...........KIDDILRLVDALAKDLGNQVYDVVALMEGFAYGAVQ.LL
APV17     253  LTRGLMPLYE...........KISSLLDLIDQLASTLHDNVYEVIGILEGLAYAAVQ.LH
HPIV2     259  ISHYLSPLGP...........RIDRLFSIVDELAQLLGDTVYKVIASLESLVYGCLQ.LK
MuV       259  ASHYLSPLKK...........RIEILLTLVDDLALLMGDKVYGVVSSLESFVYAQLQ.YG
PIV5      257  VSTYLNPLKK...........RIKYLLSLVDNLAFQIGDAVYNIIALLESFVYAQLQ.MS
WTSPV     259  LQDALPRLDD...........CLNWFLSFFDRFFTCLGRDTFDIIELIEAFLIGAIQ.CQ
WHPV      255  ..DNISEKRS...........NLRVIWDQIDRIICDHGPKGYQLVGFLEPLLVGSVE.VK
GPV       255  SDHRFTNFRT...........RGYKLWELLDSLFEDLGNDNYDLVAMIEPLVLGFLQ.LK
FPaV      252  SDYRYKQFKV...........KGHKLWSLIDSLFIDLGNETYNIVALIEPLVLGFLQ.LY
HMPV      225  VSNSLNENQEGLGLRSNLQGILTNKLYETVDYMLSLCCNEGFSLVKEFEGFIMSEIL.RI
AMPV      225  ISNSLNKNQEGLGLRSNLQGALVNKLYEIVDSMLSVCSNEGFTLVKEFEGFIMSEIL.KI
RSV       288  ISNCLNTLNKSLGLRCGFNNVILTQLFLYGDCILKLFHNEGFYIIKEVEGFIMSLIL.NI
MPV       238  LSNVLNTLNEGLGLRCRLKGHLLSKLYISTDIFLSSTSNEFYNVVKEFEGFIMSLIL.KQ
EBOV      256  IAEVEDPVCSDYP.....DFKIVSMLYQSGDYLLSILGSDGYKIIKFLEPLCLAKIQ.LC
VSV       252  IDNLFSEQ..........DIFSLLNIYRIGDKIVERQGNFSYDLIKMVEPICNLKLMKLA
RABV      264  PEPRYSDD..........LISQLCQLYIAGDQVLSMCGNSGYEVIKILEPYVVNSLVQRA
```

```
NiV(B)   302  DEAR.ILRG...AFLHHCIKEMHQELSECG......F.........TDQKIRSMFI....
NiV(M)   302  DEAR.ILRG...AFLHHCIKEMHQELSECG......F.........TDQKIRSMFI....
HeV      302  DEAR.ILRG...AFLHHCIKEIHQELIGCG......F.........TDQKTRSIFI....
MojV     301  DKAV.ALVG...SFLSFCISEIRSELVKNG......F.........HDEQDIEDFT....
LayV     301  DKAV.ALVG...SFLSFCINEIKEELIKNG......F.........NDEQDINEFT....
GhV      302  DRSQ.QLRG...AFLHHCIKDLKSELVSNG......F.........TDDEKIERFC....
PIV3     303  DPVK.QLRG...AFLNHVLSEMELIFESRE......S.........IKEFLSVDYI....
SeV      303  DPVI.PLRG...AFMRHVLTELQAVLTSRD......V.........YTDAEADTIV....
GSqRV    303  DPVI.PLKG...AFMRHVVTELHAAIIGAG......S.........YTEPEADMVT....
MeV      295  DITV.ELRG...AFLNHCFTEIHDVLDQNG......F.........SDEGTYHELI....
RDV      295  DVTT.ELRG...AFLNHCFTEIREILNQNG......V.........TDDNTYHEIV....
CDV      295  DITF.SLRG...AFLSHCFAEIQEILQDNG......F.........YTEETFQTLT....
BPV      299  DKSP.DLKG...AFLKYALDELVNELESNG......I.........TNPDDIEIML....
BeV      300  DSST.FLRG...AFLDYCLHDLINIYVENG......Y.........DNPEDHDRIL....
PMPV1    298  DESN.LLRG...AFLQFALGEIENEFKSHG......F.........TSRADLDLVL....
MMLPV1   299  DESY.LLQG...AFLQFCLNEIMNEFSNLG......F.........DHKDDHHEIL....
RuV      300  DESH.ILRG...AFLKYCLNELNNELINNG......F.........DDDKDIDFIK....
BaVV     298  DEAP.ILAG...AFLEHCFGEVVNELNNTG......Y.........TSEVDQKEII....
ASPV     306  DPVE.EIKG...AFLEFVINELSDALSKDD......W.........YTPKRVSAFS....
FLDV     296  DPIK.EIRG...AFLYYNLSELDQLLKEDT......I.........LSTGEVKDFI....
APV      301  EPSE.LFAG...NFLSFNLHELKDILLPH.................FRIDIVQRII....
PPMV1    301  EPSG.TFAG...DFFAFNLQELKDTLIEL.................LPTDVAESVT....
NDV      301  EPSG.TFAG...DFFAFNLQELKDTLIEL.................LPNNIAESVT....
APV17    301  EPSG.KFAG...DFFEFNLKEISSVLKDR.................LDAPLVKRVL....
HPIV2    307  DPVV.ELAG...SFHSFITQEIIDILIGSK......A.........LDKDESITVT....
MuV      307  DPVV.DIKG...TFYGFICNEILDLLTEDN......I.........FTEEEANKVL....
PIV5     305  DPIP.ELRG...QFHAFVCSEILDALRGTN......S.........FTQDESRTVT....
WTSPV    307  DDSP.SLRG...HFLADTLYSFREILEDNM......F.........QD...IDTII....
WHPV     301  GEIISETQF...DFLEKQCEEFLETAEAIG......I.........NEEEAQ........
GPV      303  DESE.LLRG...AFLQYCFTEVEEILVSHG......Y.........DNSQDIATVI....
FPaV     300  DDSY.LLRG...AFLEYCLRELKNELKNNG......F.........ESDHDYNIIL....
HMPV     284  TEHA.QFST...RFRNTLLNGLTDQLTKLKNKNRLRVHGTVLENND..YPMYEVVLKLLG
AMPV     284  TEHA.QFST...RFRNTLLNGLVDQLAKMRGLNRKRVSGTVLEGNQ..YPMYETTLATLG
RSV      347  TEED.QFRK...RFYNSMLNNITDAANKAQKNLLSRVCHTLLDKTVSDNIINGRWIILLS
MPV      297  TEEA.LFST...RFYNNMLNNLIDAIDRARLEYLARCAN.SAARIN..LPSTDVMIASLG
EBOV     310  SKYT.ERKG...RFLTQMHLAVNHTLEEITEMRALKP.........SQDQKIREFH....
VSV      302  RESR.PLVPQFPHFENHIKTSVDEGAK.ID...................RGIRFLH....
RABV     314  EKFR.PLIHSLGDFPVFIKDKVSQLEETFG...................PCARRFF....
```

```
NiV(B)   339  ..DDLLSILNIDNIHLLAEFFSFFRTFGHPILEAKVAAEKVREHMLADKVLEYAPIMKAH
NiV(M)   339  ..DDLLSILNIDNIHLLAEFFSFFRTFGHPILEAKVAAEKVREHMLADKVLEYAPIMKAH
HeV      339  ..DDLLSVMNIDNIHLLAEFFSFFRTFGHPILEAKTAADKVREHMLADKVLEYGPIMKAH
MojV     338  ..SRIINIMSLPDIHLVAEFFSFFRTFGHPTLEALTAAEKVRTHMQKPKILDFLPIMKAH
LayV     338  ..SRIINIMSIPDIHLVAEFFSFFRTFGHPTLEALTAAEKVRTHMQKPKILEFLPIMKAH
GhV      339  ..DKLVNILNNNDIHMIAEMFSFFRTFGHPTLEAVTAASKVRDYMKADKVLEYETIMKGH
PIV3     340  ..DKILDIFDKSTIDEIAEIFSFFRTFGHPPLEASIAAEKVRKYMYIEKQLKFDTINKCH
SeV      340  ..ESLLAIFHGTSIDEKAEIFSFFRTFGHPSLEAVTAADKVRAHMYAQKAIKLKTLYECH
GSqRV    340  ..DSILNIFKDTTIDEKAEIFSFFRTFGHPSLEAVTAADKVRAHMYASKSIKLRTLYECH
MeV      332  ..EALDYIFITDDIHLTGEIFSFFRSFGHPRLEAVTAAENVRKYMNQPKVIVYETLMKGH
RDV      332  ..EALDYIFITDDIHLTGEIFSFFRSFGHPRLEAITAADNVRKHMNQPKVIVYETLMKGH
CDV      332  ..QALDFVFITEDIHITGEIFSFFRSFGHPRLEAITAAENVRKHMNQPKVVSYETMMKGH
BPV      336  ..DAVEKIFNIDDIHMVAEFFSFFRTFGHPILEASEAATKVREHMNKPKIIDFQIMMKGH
BeV      337  ..NIIDHIFSIDDIHMVSEFFSFFRTFGHPTLEAENAADKVRAHMNKPKLIKFSTMMKAH
PMPV1    335  ..DTITNIFTIDDIHMISEFFSFFRTFGHPTLEAKEAASKVREHMNKPKIVSFEIMMKGH
MMLPV1   336  ..ATIYKIFDIEDLHMIAEFFSFFRTFGHPTLEAEEAAAKVRAHMNKPKVIKFQTMMKGH
RuV      337  ..SSFFWIFNLSDIHLIGEFFSFFRTFGHPILEAKEAADKVRSHMNKPKLIDFKIMMKGH
BaVV     335  ..RIIREIFNLDDVHLLAEMFSFFRSFGHPILEAEEAAHKVREHMHKPKLISFEVLMKGH
ASPV     343  ..RKLLGILSTDLVSEAAEIFSFFRTFGHPALEAVQAAEKVRKYMYEPKILSFETIMQAH
FLDV     333  ..ELISEIINMGSVSEISEIFSFFRSFGHPVLEAVTAADKVRDHMCKPKVLDYETLQKGH
APV      336  ..TIVSEVFSGLDQTQSAEMLCMLRLWGHPLLESRAAASAVRKQMCAPKLLDFDTVLQVL
PPMV1    336  ..HVIASIFSGLEQNQAAEMLCLLRLWGHPLLESRSAAKAVRSQMCAPKMVDFDMILQVL
NDV      336  ..HAIATVFSGLEQNQAAEMLCLLRLWGHPLLESRSAARAVRSQTCAPKMVDFDMILQVL
APV17    336  ..DAVTWIYSGLTDNQSAEMLCILRLWGHPTLESRAAATAVRKQMCAAKVVDFDMIQQVL
HPIV2    344  ..TQLLDIFSNLSPDLIAEMLCLMRLWGHPTLTAAQ.VGKVRESMCAGKLLDFPTIMKTL
MuV      344  ..LDLTSQFDNLSPDLTAELLCIMRLWGHPTLTASQAASKVRESMCAPKVLDFQTIMKTL
PIV5     342  ..TNLISPFQDLTPDLTAELLCIMRLWGHPMLTASQAAGKVRESMCAGKVLDFPTIMKTL
WTSPV    341  ..EQLRTFLTDHDVEVYAELFCAFRLWGHPPVEASPAAQKMREYMNAPAYTSMRSLLKNQ
WHPV     335  ...GLLYDITAGTGEWCAEKMAMFRTWGHPPLEASPAAAKIRPHMCSSRLLEPLFLFKEE
GPV      340  ..ETFTDIFQNKDIHMIAEFFSFFRTFGHPTLEAEEAATKVRTHMNKPKVINFEIMMKGH
FPaV     337  ..SSLFDIFSIDDIHMIAEYFSFFRTFGHPTLEATEAANKVREYMNKPKLINFQVMMKGH
HMPV     338  DTLRCIKLLINKNLENAAELYYIFRIFGHPMVDERDAMDAVKLNNEITKILRWESLTELR
AMPV     338  GALKTIRLLVNKNLDNAAELYYIFRIFGHPMVEEREAMDAVRLNNEITKILKLESLTELR
RSV      403  KFLKLIKLAGDNNLNNLSELYFLFRIFGHPMVDERQAMDAVKVNCNETKFYLLSSLSMLR
MPV      350  DILSLINVLGESNLNNLSELYFIFRIFGHPMVDERKAMDAVRDNCCETKFLTAKNLASLR
EBOV     353  ..RTL..IRLEMTPQQLCELFSIQKHWGHPVLHSETAIQKVKKHATVLKALRPIVIFETY
VSV      337  .....DQIMSVKTVDLTLVIYGSFRHWGHPFIDYYTGLEKLHSQVTMKKDIDVSYAKALA
RABV     350  .....RALDQFDNIHDLVFVFGCYRHWGHPYIDYRKGLSKLYDQVHLKKMIDKSYQECLA
```

```
NiV(B)   397  AIFCGTIINGYRDRHGGAWPPLYLPAHASKHIIRLKNSGES.LTI..DDCVKNWESFCGI
NiV(M)   397  AIFCGTIINGYRDRHGGAWPPLYLPAHASKHIIRLKNSGES.LTI..DDCVKNWESFCGI
HeV      397  AVFCGTIINGYRDRHRGAWPPLYLPSHASKHIIRLKNSGES.LTV..DDCVKNWESFCGI
MojV     396  AIFCGIIINGYRDRHGGAWPPVIFPEHVSVLIKRLHNSSEA.LTH..EICCQEWKSFCGF
LayV     396  AIFCGIIINGYRDRHGGAWPPVVFPEHASVLIKRLHNSSEA.LTH..EICCQEWKSFCGF
GhV      397  AIFCGIIINGYRDKHGGVWPPLTFPKHVHQEIKRLQSSGER.LTY..EICIKHWKSFCGL
PIV3     398  AIFCTIIINGYRERHGGQWPPVTLPDHAHEFIINAYGSNSA.ISY..ENAVDYYQSFIGI
SeV      398  AVFCTIIINGYRERHGGQWPPCDFPDHVCLELRNAQGSNTA.ISY..ECAVDNYTSFIGF
GSqRV    398  AIFCTIIINGYRERHGGQWPPCSFPEHVCLELRNAQGSNSA.ISY..ECAVDNYMSFIGF
MeV      390  AIFCGIIINGYRDRHGGSWPPIHLPLHAADTIRNAQASGEG.LTH..EQCVDNWKSFAGV
RDV      390  AIFCGIIINGYRDRHGGSWPPIHLPVHAATSIRNAQASGEG.LTY..EQCVDNWKSFAGI
CDV      390  AIFCGIIINGYRDRHGGTWPPMDLPVHASPIIRNAHASGEG.ITY..SQCIENWKSFAGI
BPV      394  ALFCGFIINGFRERHGGSWPPLKLPDHSSKKIKNAQLNNES.LTD..DVCIEEWKSFVGL
BeV      395  ALFCGTIINGYRDRHGGMWPPLTLGDHASSKIKQCMRNHES.LTH..ELCIENWKSFCGI
PMPV1    393  ALFCGIIINGFRDRHGGAWPPHEFPEHVSQLIKNAQTNSEA.LTH..ELCIQEWKSFVGF
MMLPV1   394  ALFCGTIINGFRDRHGGAWPPVVFPNHVSKRLKQLHLNSES.LTD..EICIQEWKSFVGF
RuV      395  AIFCGTIINGYRERHGGTWPPLILPKHASHEIINLKNNDEG.LTD..EMCIVHWKSFVGL
BaVV     393  AIFCATIINGYRDRHGGAWPPLQLPSHATTAIKTAQVNNEA.ITD..ALAVTHWKSFCGL
ASPV     401  AIFCGIIIGGYRERHGGQWPPCTIPEHASNSIKAAKLQGSA.LTH..EMCYDNWRSFTGF
FLDV     391  AVFCSMIINGFRERHGGSWPPCFLPDYASSELKAVMANHAA.IPY..ELSVNNWESFIGF
APV      394  SFFKGTIINGYRKKNAGVWPRVKQSTVYGSTITQLHADSAE.ISH..EIMLKEYKNLSAL
PPMV1    394  SFFKGTIINGYRKKNSGVWPRVKTDTIYGKVIGQLHADSAE.ISH..DVMLREYKSLSAL
NDV      394  SFFKGTIINGYRKKNSGVWPRVKVDTIYGNIIGQLHADSAE.ISH..DVMLREYKSLSAL
APV17    394  AFFKGTLINGYRRQNSGVWPRVKRHTVYGPTLEQLHADSAE.ISH..ELMLKEYRRLAAI
HPIV2    401  AFFHTILINGYRRKKNGMWPPLILPKNASKSLIEFQHDNAE.ISY..EYTLKHWKEISLI
MuV      402  AFFHAILINGYRRSHNGIWPPTTLHGNAPKSLIEMRHDNSE.LKY..EYVLKNWKSISML
PIV5     400  AFFHTILINGYRRKHHGVWPPLNLPGNASKGLTELMNDNTE.ISY..EFTLKHWKEISLI
WTSPV    399  RAFRVMLINGYIRQNGGTWPNVELDPSRAPKLLTLRMTGKG.IPL..EDIDSLANEIDTI
WHPV     392  SFFKREIINGHIKKQAGMWPSCSIPIW.CDELRTLRSTGRP.IPQ..NPSSKIMDELLCT
GPV      398  ALFCGIIINGYRERHGGAWPPHEFPLHVSKNIKTCAANCEA.LTH..EICINEWKSFVGF
FPaV     395  ALFCGTIINGFRDQHGGSWPPVILPDHVDKRIKHAYLNNEG.LTD..EMCITSWKSFVGF
HMPV     398  GAFILRIIKGFVDNN.KRWPKIKNLKVLSKRWTMYFKAKSYPSQL..ELSEQDFLELAAI
AMPV     398  GAFILRIIKGFVDTN.KRWPKIKNLKVLSRRWIMYFKAKSYPSQL..ELSSQDFLELAGV
RSV      463  GAFIYRIIKGFVNNY.NRWPTLRNAIVLPLRWLTYYKLNTYPSLL..ELTERDLIVLSGL
MPV      410  GAYVYRIIKGFVANY.NRWPYIKTRVCLTPTWINYLDTNSCPSLL..EMTEDDFIVLAGV
EBOV     409  CVFKYSIAKHYFDSQ.GSWYSVTSDRNLTPGLNSYIKRNQF.PPL..PMIKELLWEFYHL
VSV      392  SDLARIVLFQQFNDH.KKWFVNGDLLPHDHPFKSHVKENTWPTAAQVQDFGDKWH...EL
RABV     405  SDLARRILRWGFDKY.SKWYLDSRFLARDHPLTPYIKTQTWPPKHIVDLVGDTWH...KL
```

```
NiV(B)   454  QFDCFMELKLDSDLSMYMKDKALSPIKDEWDSVYPREVLSYTPPK..........STEP
NiV(M)   454  QFDCFMELKLDSDLSMYMKDKALSPIKDEWDSVYPREVLSYTPPK..........STEP
HeV      454  QFDCFMELKLDSDLSMYMKDKALSPIKEEWDSVYPREVLNYTPPR..........STEP
MojV     453  KFKCFLPLELDNDLSMYMKDKALSPVKNEWDSVYPYESMCYNPPA..........STTS
LayV     453  KFKCFLPLELDSDLSMYMKDKALSPIKDEWDSVYPSESMCYSPPN..........STTS
GhV      454  NFKCFMDLNLDEDLSMYMKDKALAAIRNEWDSVYPKENLSYQPTK..........NTTP
PIV3     455  KFNKFIEPQLDEDLTIYMKDKALSPKKSNWDTVYPASNLLYRTNA..........SNES
SeV      455  KFRKFIEPQLDEDLTIYMKDKALSPRKEAWDSVYPDSNLYYKAPE..........SEET
GSqRV    455  KFQKFIDPQLDEDLTIYMKDKALSPRREAWDSVYPESNLYYKVPE..........SDET
MeV      447  RFGCFMPLSLDSDLTMYLKDKALAALQREWDSVYPKEFLRYDPPK..........GTGS
RDV      447  RFGCFMPLSLDSDLTMYLKDKALAALKKEWDSAYPKEFLRYNPPK..........STSS
CDV      447  RFKCFMPLSLDSDLTMYLKDKALAALRKEWDSVYPKEFLRYNPPR..........STES
BPV      451  RFGCFMSLTLDDDLTMFMKDKALAALKSEWDSVYPRETMVYNPPA..........QTSS
BeV      452  KFGCFMGLSLDEDLTMYMKDKALSAIKQEWDSVYPRSVMIYDAPQ..........QSTS
PMPV1    450  KFKCFMSLTLDEDLTMYMKDKALASIKKEWDSVYPREFMTYTPPK..........QTTS
MMLPV1   451  KFKCFMPLTLDEDLTMYMKDKALAAISSEWDSVYPRETMAYTPPK..........QTTS
RuV      452  KFHCFMPLVLDDDLTMYMKDKALASPLSEWDSIYPKEVIDYVNSS..........STIS
BaVV     450  TFSCFMPLSLDEDLTMYMKDKALAAISKEWDSPYVYDVMKYRPPK..........QTTS
ASPV     458  KFEKFLEPQLCEDLTIYMKDKALSPPKSNWDSVYPNHNMCYNPNV..........CEES
FLDV     448  KFDKFEEVNLDEDLTIFMKDKALSPIRAEWDTVYPQENMQYSPGR..........SSTS
APV      451  EFEQCIDFDPVSNLSMFLKDKAIAHPKHNWLASFRKNLLSDTQKKE.....VRD..ATST
PPMV1    451  EFEPCIDYDPVTNLSMFLKDKAIAHPKDNWLASFRRNLLSEDQKKQ.....VKE..ATST
NDV      451  EFEPCIDYDPVTNLSMFLKDKAIAHPSDNWLASFRRNLLSEDQKKQ.....IEE..ATST
APV17    451  EFEKCIDVDPVSNLSMFLKDKAIAHTKPNWLASFKANLLSDCQRAT.....SKD..VAST
HPIV2    458  EFRKCFDFDPGEELSIFMKDKAISAPRSDWMSVFRRSLIKQRHQRH.....HIPMPNPFN
MuV      459  RIHKCFDASPDEDLSIFMKDKAISCPKQDWMGVFRRSLIKQRYRDA.....NRPLPQPFN
PIV5     457  KFKKCFDADAGEELSIFMKDKAISAPKQDWMSVFRRSLIKQRHQHH.....QVPLPNPFN
WTSPV    456  HFQKTFDIDLENDFSIYFKDKAIAPTVDHWDSTYQWSSMQYVPKI...........YHGH
WHPV     448  NIDQIEDVSLDEAISIYMKDKAISGPASSFDSSFQFCGMSYRPSR..........GNYT
GPV      455  KFKCFMPLTLDEDLTMYMKDKALAAIAPEWDSVYPRETLSYTPPK..........QTTS
FPaV     452  KFKCFMPLVLDEDLTMYMKDKALAAIKPEWDSVYPRENMCYITPH..........QSTS
HMPV     455  QFEQEFSVPEKTNLEMVLNDKAISPPKRLIWSVYPKNYLPEKIKNRYLEETFNASDSLKT
AMPV     455  QFEQEFAIPERTNLEMVLNDKAISPPKNLIWSVFPKNYLPTNIRERFTEEMFNSSEKLKT
RSV      520  RFYREFRLPKKVDLEMIINDKAISPPKNLIWTSFPRNYMPSHIQNYIEHEKLKFSESDKS
MPV      467  HFIREFHIPKLTDLEIILNDKAISPPKSLIWSCFPKNYIPQVIQDEYARRYCRAKAPLKT
EBOV     465  DHPPLFSTKIISDLSIFIKDRATAVERTCWDAVFEPNVLGYNPPH..........KFST
VSV      448  PLIKCFEIPDLLDPSIIYSDKSHSMNRSEVLKHVRMN.....PNT..........PIPS
RABV     461  PITQIFEIPESMDPSEILDDKSHSFTRTRLASWLSEN.....RGG..........PVPS
```

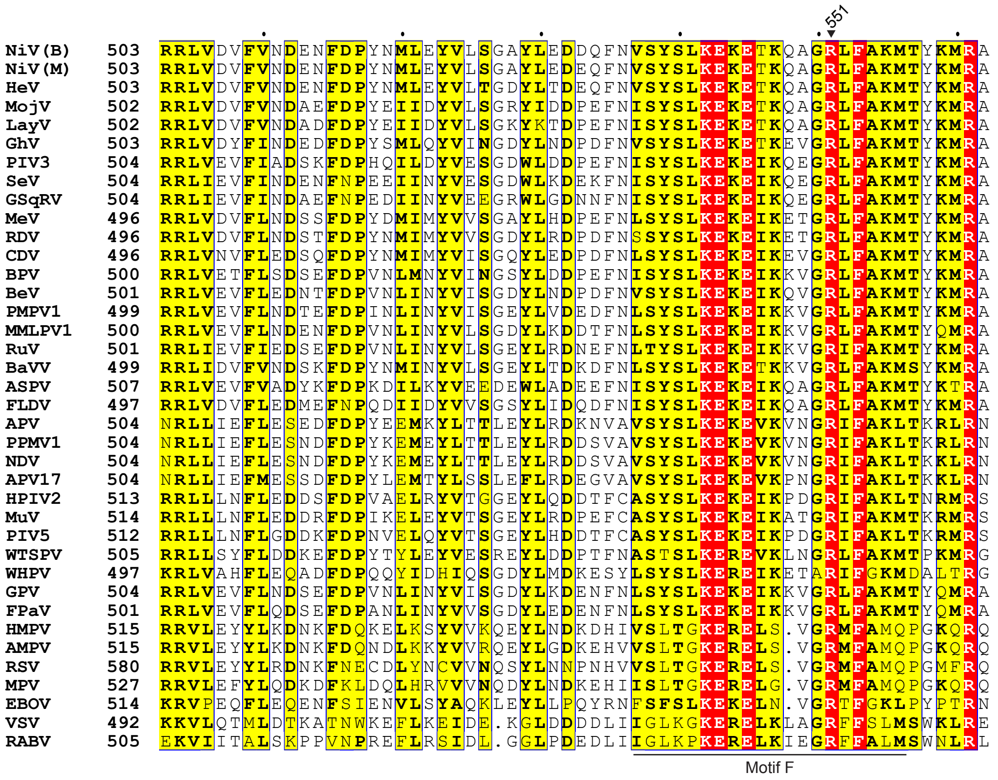
551
Motif F

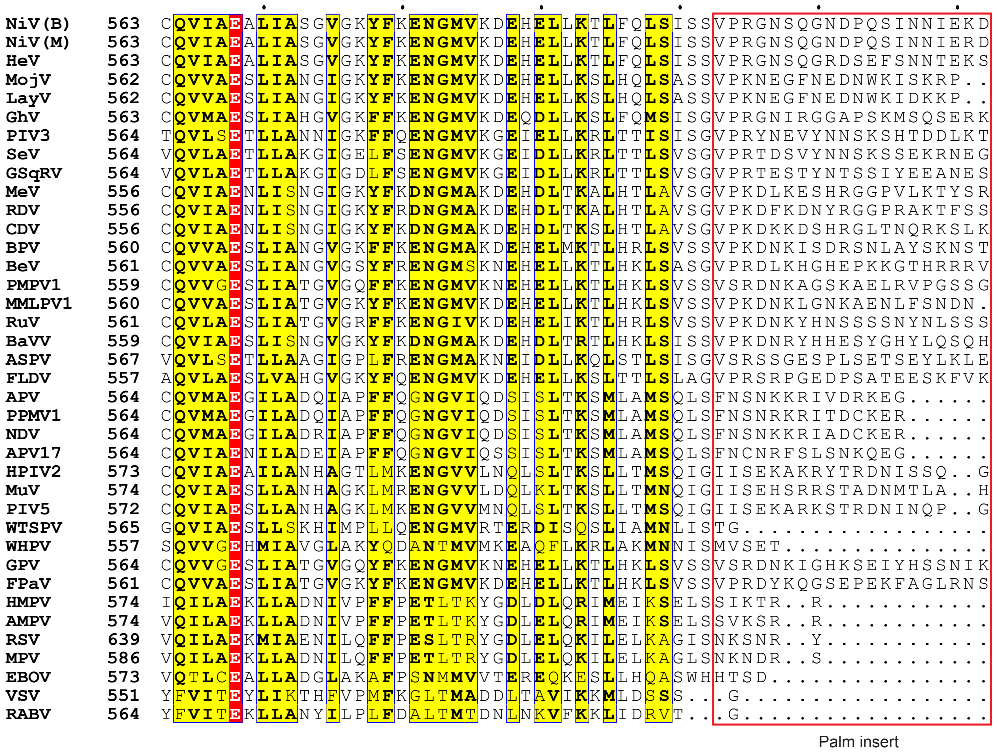
Palm insert

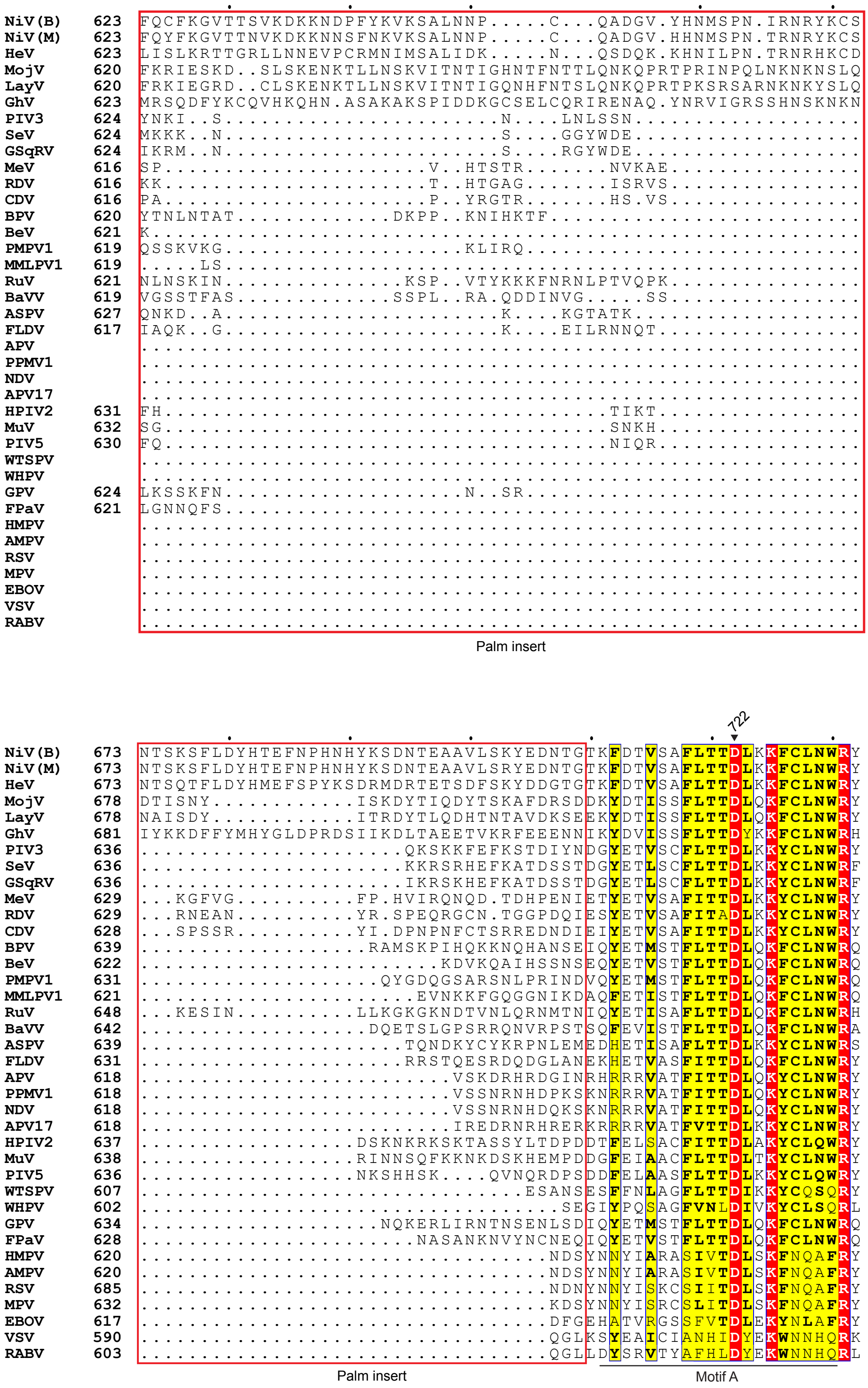
Palm insert
722
Palm insert
Motif A

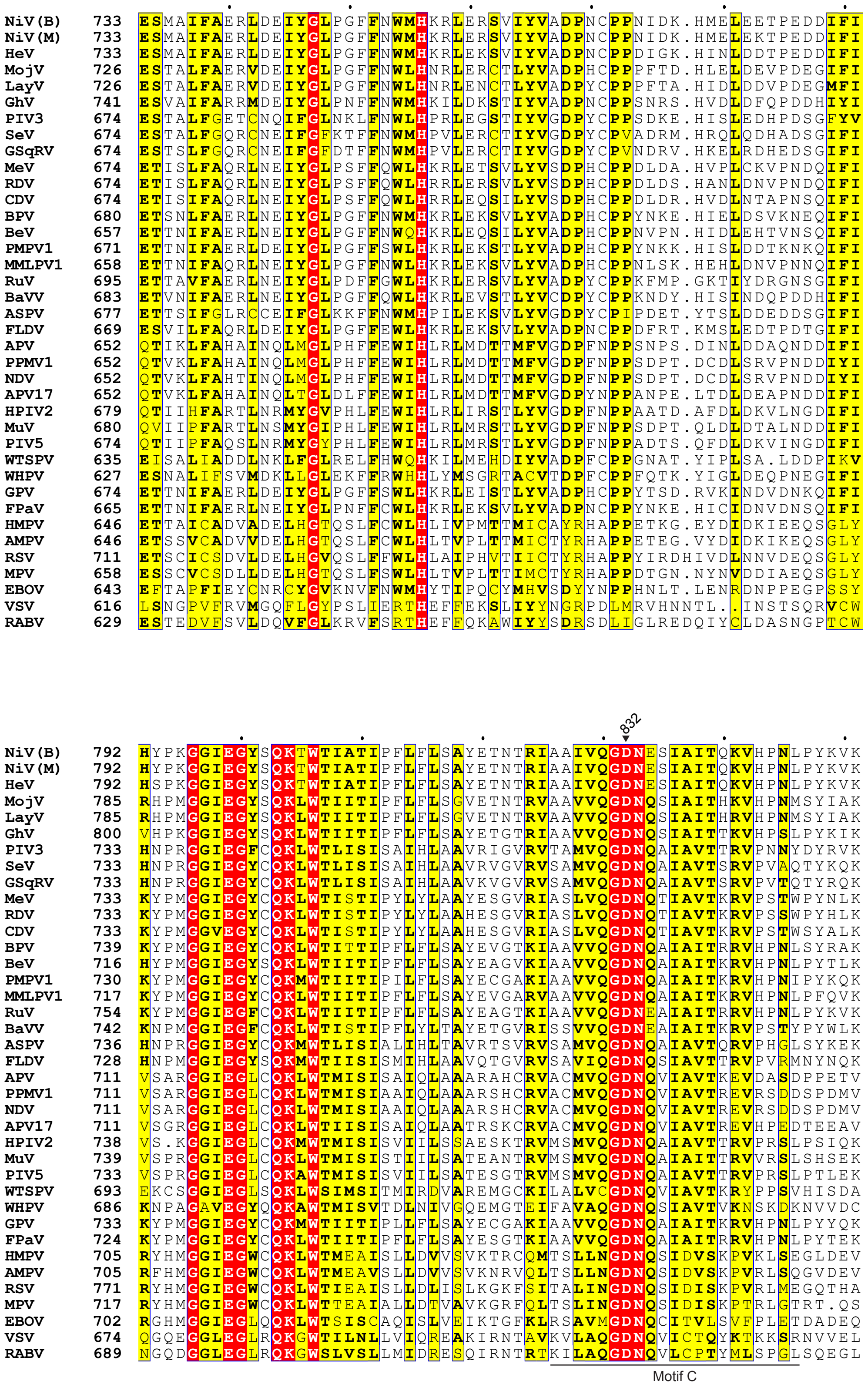

NiV(B) 733 ESMAIFAERLDEIYGLPGFFNWMHKRLERSVIYVADPNCPPNIDK.HMELEETPEDDIFI
NiV(M) 733 ESMAIFAERLDEIYGLPGFFNWMHKRLERSVIYVADPNCPPNIDK.HMELEKTPEDDIFI
HeV 733 ESMAIFAERLDEIYGLPGFFNWMHKRLEKSVIYVADPNCPPDIGK.HINLDDTPEDDIFI
MojV 726 ESTALFAERVDEIYGLPGFFNWLHNRLERCTLYVADPHCPPPFTD.HLELDEVPDEGIFI
LayV 726 ESTALFAERVDEIYGLPGFFNWLHNRLENCTLYVADPYCPPPFTA.HIDLDDVPDEGMFI
GhV 741 ESVAIFARRMDEIYGLPNFFNWMHKILDKSTIYVADPNCPPSNRS.HVDLDFQPDDHIYI
PIV3 674 ESTALFGETCNQIFGLNKLFNWLHPRLEGSTIYVGDPYCPPSDKE.HISLEDHPDSGFYV
SeV 674 ESTALFGQRCNEIFGFKTFFNWMHPVLERCTIYVGDPYCPVADRM.HRQLQDHADSGIFI
GSqRV 674 ESTSLFGQRCNEIFGFDTFFNWMHPVLERCTIYVGDPYCPVNDRV.HKELRDHEDSGIFI
MeV 674 ETISLFAQRLNEIYGLPSFFQWLHKRLETSVLYVSDPHCPPDLDA.HVPLCKVPNDQIFI
RDV 674 ETISLFAQRLNEIYGLPSFFQWLHRRLERSVLYVSDPHCPPDLDS.HANLDNVPNDQIFI
CDV 674 ETISIFAQRLNEIYGLPSFFQWLHRRLEQSILYVSDPHCPPDLDR.HVDLNTAPNSQIFI
BPV 680 ETSNLFAERLNEIYGLPGFFNWMHKRLEKSVLYVADPHCPPYNKE.HIELDSVKNEQIFI
BeV 657 ETTNIFAERLNEIYGLPGFFNWQHKRLEQSILYVADPHCPPNVPN.HIDLEHTVNSQIFI
PMPV1 671 ETTNIFAERLDEIYGLPGFFSWLHKRLEKSTLYVADPHCPPYNKK.HISLDDTKNKQIFI
MMLPV1 658 ETTNIFAQRLNEIYGLPGFFNWLHKRLEKSVLYVADPHCPPNLSK.HEHLDNVPNNQIFI
RuV 695 ETTAVFAERLNEIYGLPDFFGWLHKRLERSVLYVADPYCPPKFMP.GKTIYDRGNSGIFI
BaVV 683 ETVNIFAERLNEIYGLPGFFQWLHKRLEVSTLYVCDPYCPPKNDY.HISINDQPDDHIFI
ASPV 677 ETTSIFGLRCCEIFGLKKFFNWMHPILEKSVLYVGDPYCPIPDET.YTSLDDEDDSGIFI
FLDV 669 ESVILFAQRLDEIYGLPGFFEWLHKRLEKSVLYVADPNCPPDFRT.KMSLEDTPDTGIFI
APV 652 QTIKLFAHAINQLMGLPHFFEWIHLRLMDTTMFVGDPFNPPSNPS.DINLDDAQNDDIFI
PPMV1 652 QTVKLFAHAINQLMGLPHFFEWIHLRLMDTTMFVGDPFNPPSDPT.DCDLSRVPNDDIYI
NDV 652 QTVKLFAHTINQLMGLPHFFEWIHLRLMDTTMFVGDPFNPPSDPT.DCDLSRVPNDDIYI
APV17 652 QTVKLFAHAINQLTGLDLFFEWIHLRLMDTTMFVGDPYNPPANPE.LTDLDEAPNDDIFI
HPIV2 679 QTIIHFARTLNRMYGVPHLFEWIHLRLIRSTLYVGDPFNPPAATD.AFDLDKVLNGDIFI
MuV 680 QVIIPFARTLNSMYGIPHLFEWIHLRLMRSTLYVGDPFNPPSDPT.QLDLDTALNDDIFI
PIV5 674 QTIIPFAQSLNRMYGYPHLFEWIHLRLMRSTLYVGDPFNPPADTS.QFDLDKVINGDIFI
WTSPV 635 EISALIADDLNKLFGLRELFHWQHKILMEHDIYVADPFCPPGNAT.YIPLSA.LDDPIKV
WHPV 627 ESNALIFSVMDKLLGLEKFFRWHHLYMSGRTACVTDPFCPPFQTK.YIGLDEQPNEGIFI
GPV 674 ETTNIFAERLDEIYGLPGFFSWLHKRLEISTLYVADPHCPPYTSD.RVKINDVDNKQIFI
FPaV 665 ETTNIFAERLNEIYGLPNFFCWLHKRLEKSVLYVADPNCPPYNKE.HICIDNVDNSQIFI
HMPV 646 ETTAICADVADELHGTQSLFCWLHLIVPMTTMICAYRHAPPETKG.EYDIDKIEEQSGLY
AMPV 646 ETSSVCADVVDELHGTQSLFCWLHLTVPLTTMICTYRHAPPETEG.VYDIDKIKEQSGLY
RSV 711 ETSCICSDVLDELHGVQSLFFWLHLAIPHVTIICTYRHAPPYIRDHIVDLNNVDEQSGLY
MPV 658 ESSCVCSDLLDELHGTQSLFSWLHLTVPLTTIMCTYRHAPPDTGN.NYNVDDIAEQSGLY
EBOV 643 EFTAPFIEYCNRCYGVKNVFNWMHYTIPQCYMHVSDYYNPPHNLT.LENRDNPPEGPSSY
VSV 616 LSNGPVFRVMGQFLGYPSLIERTHEFFEKSLIYYNGRPDLMRVHNNTL..INSTSQRVCW
RABV 629 ESTEDVFSVLDQVFGLKRVFSRTHEFFQKAWIYYSDRSDLIGLREDQIYCLDASNGPTCW
832
NiV(B) 792 HYPKGGIEGYSQKTWTIATIPFLFLSAYETNTRIAAIVQGDNESIAITQKVHPNLPYKVK
NiV(M) 792 HYPKGGIEGYSQKTWTIATIPFLFLSAYETNTRIAAIVQGDNESIAITQKVHPNLPYKVK
HeV 792 HSPKGGIEGYSQKTWTIATIPFLFLSAYETNTRIAAIVQGDNESIAITQKVHPNLPYKVK
MojV 785 RHPMGGIEGYSQKLWTIITIPFLFLSGVETNTRVAAVVQGDNQSIAITHKVHPNMSYIAK
LayV 785 RHPMGGIEGYSQKLWTIITIPFLFLSGVETNTRVAAVVQGDNQSIAITHRVHPNMSYIAK
GhV 800 VHPKGGIEGYSQKLWTIITIPFLFLSAYETGTRIAAVVQGDNQSIAITTKVHPSLPYKIK
PIV3 733 HNPRGGIEGFCQKLWTLISISAIHLAAVRIGVRVTAMVQGDNQAIAVTTRVPNNYDYRVK
SeV 733 HNPRGGIEGYCQKLWTLISISAIHLAAVRVGVRVSAMVQGDNQAIAVTSRVPVAQTYKQK
GSqRV 733 HNPRGGIEGYCQKLWTLISISAIHLAAVKVGVRVSAMVQGDNQAIAVTSRVPVTQTYRQK
MeV 733 KYPMGGIEGYCQKLWTISTIPYLYLAAYESGVRIASLVQGDNQTIAVTKRVPSTWPYNLK
RDV 733 KYPMGGIEGYCQKLWTISTIPYLYLAAHESGVRIASLVQGDNQTIAVTKRVPSSWPYHLK
CDV 733 KYPMGGVEGYCQKLWTISTIPYLYLAAHESGVRIASLVQGDNQTIAVTKRVPSTWSYALK
BPV 739 KYPMGGIEGYCQKLWTITTIPFLFLSAYEVGTKIAAVVQGDNQAIAITRRVHPNLSYRAK
BeV 716 HYPMGGIEGYSQKLWTIITIPFLFLSAYEAGVKIAAVVQGDNQAIAITKRVHPNLPYTLK
PMPV1 730 KYPMGGIEGYCQKMWTIITIPILFLSAYECGAKIAAVVQGDNQAIAITKRVHPNIPYKQK
MMLPV1 717 KYPMGGIEGYCQKLWTIITIPFLFLSAYEVGARVAAVVQGDNQAIAITKRVHPNLPFQVK
RuV 754 KYPMGGIEGFCQKLWTIITIPFLFLSAYEAGTKIAAVVQGDNEAIAITRRVHPNLPYKVK
BaVV 742 KNPMGGIEGFCQKLWTISTIPFLYLTAYETGVRISSVVQGDNEAIAITKRVPSTYPYWLK
ASPV 736 HNPRGGIEGYCQKMWTLISIALIHLTAVRTSVRVSAMVQGDNQAIAVTQRVPHGLSYKEK
FLDV 728 HNPMGGIEGYSQKMWTIISISMIHLAAVQTGVRVSAVIQGDNQSIAVTTRVPVRMNYNQK
APV 711 VSARGGIEGLCQKLWTMISISAIQLAAARAHCRVACMVQGDNQVIAVTKEVDASDPPETV
PPMV1 711 VSARGGIEGLCQKLWTMISIAAIQLAAARSHCRVACMVQGDNQVIAVTREVRSDDSPDMV
NDV 711 VSARGGIEGLCQKLWTMISIAAIQLAAARSHCRVACMVQGDNQVIAVTREVRSDDSPDMV
APV17 711 VSGRGGIEGLCQKLWTIISISAIQLAATRSKCRVACMVQGDNQVIAVTREVHPEDTEEAV
HPIV2 738 VS.KGGIEGLCQKMWTMISISVIILSSAESKTRVMSMVQGDNQAIAVTTRVPRSLPSIQK
MuV 739 VSPRGGIEGLCQKLWTMISISTIILSATEANTRVMSMVQGDNQAIAITTRVVRSLSHSEK
PIV5 733 VSPRGGIEGLCQKAWTMISISVIILSATESGTRVMSMVQGDNQAIAVTTRVPRSLPTLEK
WTSPV 693 EKCSGGIEGLSQKLWSIMSITMIRDVAREMGCKILALVCGDNQVIAVTKRYPPSVHISDA
WHPV 686 KNPAGAVEGYQQKAWTMISVTDLNIVGQEMGTEIFAVAQGDNQSIAVTVKNSKDKNVVDC
GPV 733 KYPMGGIEGYCQKMWTIITIPLLFLSAYECGAKIAAVVQGDNQAIAITKRVHPNLPYYQK
FPaV 724 KYPMGGIEGYCQKLWTIITIPFLFLSAYESGTKIAAVVQGDNQAIAITRRVHPNLPYTEK
HMPV 705 RYHMGGIEGWCQKLWTMEAISLLDVVSVKTRCQMTSLLNGDNQSIDVSKPVKLSEGLDEV
AMPV 705 RFHMGGIEGWCQKLWTMEAVSLLDVVSVKNRVQLTSLLNGDNQSIDVSKPVRLSQGVDEV
RSV 771 RYHMGGIEGWCQKLWTIEAISLLDLISLKGKFSITALINGDNQSIDISKPVRLMEGQTHA
MPV 717 RYHMGGIEGWCQKLWTTEAIALLDTVAVKGRFQLTSLINGDNQSIDISKPTRLGTRT.QS
EBOV 702 RGHMGGIEGLQQKLWTSISCAQISLVEIKTGFKLRSAVMGDNQCITVLSVFPLETDADEQ
VSV 674 QGQEGGLEGLRQKGWTILNLLVIQREAKIRNTAVKVLAQGDNQVICTQYKTKKSRNVVEL
RABV 689 NGQDGGLEGLRQKGWSLVSLLMIDRESQIRNTRTKILAQGDNQVLCPTYMLSPGLSQEGL
Motif C

```
NiV(B)   852  KEICAK...QAQLYFERLRMNLRALGHNLKATETIISTHLFVYSKKIHYDGAVLSQALKS
NiV(M)   852  KEICAK...QAQLYFERLRMNLRALGHNLKATETIISTHLFIYSKKIHYDGAVLSQALKS
HeV      852  KEICAR...QAQLYFDRLRMNLRALGLNLKATETIISTHLFVYSKKIHYDGAVLSQALKS
MojV     845  KDVSAE...KARIYFERLRTNLDALGHNLKLTETIISTHFFVYSKRIYYDGMVLSQGLKS
LayV     845  KNVSAE...KAKIYFERLRSNLDALGHNLKLTETIISTHFFVYSKRIYYDGMVLSQGLKS
GhV      860  KEISAS...KAKEYFYRLRDNLRSLGHDMKATETVISTNFFIYSKRIYYDGVMLSQGLKT
PIV3     793  KEIVYK...DVVRFFDSLREVMDDLGHELKLNETIISSKMFIYSKRIYYDGRILPQALKA
SeV      793  KNHVYK...EITKYFGALRHVMFDVGHELKLNETIISSKMFVYSKRIYYDGKILPQCLKA
GSqRV    793  KMHVYE...EIIKYFQSLREVMIDIGHELKLNETIISSKMFVYSKRIYYDGKILPQCLKA
MeV      793  KREAAR...VTRDYFVILRQRLHDIGHHLKANETIVSSHFFVYSKGIYYDGLLVSQSLKS
RDV      793  KREAAR...VTREYFCLLRQRLHDIGHHLKANETIVSSHFFVYSKGIYYDGLLISQSLKS
CDV      793  KSEASR...VTTEYFIALRQRLHDVGHHLKANETIISSHFFVYSKGIYYDGMLISQSLKS
BPV      799  KIKSTE...MAQQYFNQLRLNLAGIGHNLKANETIISSHFFVYSKRIYYDGLVLSQALKP
BeV      776  KNMSAN...AAKEYFYILRRNLGDIGHNLKANETIISSNFFIYSKRIYYDGLVLSQALKP
PMPV1    790  KYLCSQ...LAQQYFDRLRMNMAGIGHNLKANETIVSSHFFIYSKRIYYDGQVLSQALKP
MMLPV1   777  KIISSQ...LAQKYFDRLRSNLFDIGHNLKANETIVSSHFFVYSKRIYYDGLVLSQALKP
RuV      814  KNMCVE...SAQEYFQQLRRNFHGIGHHLKAHETIVSSHFFIYSKRIYYDGVAISQSLKP
BaVV     802  KAESAQ...TAREYFKRLRFNFGMIGHNLKANETLISSHFFVYSKQIFYDGVVLSQALKS
ASPV     796  KHRVYD...AVVRYFYQMQKVMAEMGHELKLQETVISSKFFVYSKRIYYDGQILPQCLKA
FLDV     788  KTECYK...STIRYFETLRKVMGGLGHNLKLNETIISNQFFIYSKRIYFDGSILPQGLKT
APV      771  LQQLHE...ASNSFFNELMAVNHNIGHNLKDRETLRSDTFFIYSKRIFKDGTILGQALKN
PPMV1    771  LTQLHQ...ASDNFFKELIHVNHLIGHNLKDRETIRSDTFFIYSKRIFKDGAILSQVLKN
NDV      771  LTQLHQ...ASDNFFKELIHVNHLIGHNLKDRETIRSDTFFIYSKRIFKDGAILSQVLKN
APV17    771  LAELHR...ASDAFFSELKHVNHLIGHELKDRETIRSDTSFVYSKRIFKDGRILSQILKN
HPIV2    797  KELAYA...ASKLFFERLRANNYGLGHQLKAQETIISSTFFIYSKRVFYQGRILTQALKN
MuV      799  KEQAYK...ASKLFFERLRANNHGIGHHLKEQETILSSDFFIYSKRVFYKGRILTQALKN
PIV5     793  KTIAFR...SCNLFFERLKCNNFGLGHHLKEQETIISSHFFVYSKRIFYQGRILTQALKN
WTSPV    753  REDIVK...VVKDYHTNLAKRLSEVNQLLKDRETVLSSNIIIFGKRVLRNGRYLPQSLKA
WHPV     746  KKSALE...ESIRFKYHLQKLLIKKGQRLKMSETFVSPDLFVYSKRIYKNARTLPQSLKG
GPV      793  KYLCSQ...LAQQYFNRLRLNMAGIGHNLKANETIVSSHFFIYSKRIYYDGQVLSQALKP
FPaV     784  KLLCSQ...LAQKYFERLRYNMDAIGHNLKANETIVSSHFFVYSKRIYYDGLVLSQALKP
HMPV     765  KA.DYS...LAVKMLKEIRDAYRNIGHKLKEGETYISRDLQFISKVIQSEGVMHPTPIKK
AMPV     765  KA.DYS...LAVKMLKEIRNAYKDIGHKLKEGETYISRDLQFMSKVIQSEGVMHPSPIKK
RSV      831  QA.DYL...LALNSLKLLYKEYAGIGHKLKGTETYISRDMQFMSKTIQHNGVYYPASIKK
MPV      776  EA.DYD...LAINSLRLISAAYKGIGHKLKEGETYLSRDMQFMSKTIQHEGVYYPASIKK
EBOV     762  EQSAED...NAARVAASLAKVTSACGIFLKPDETFVHSGFIYFGKKQYLNGVQLPQSLKT
VSV      734  QGALNQMVSNNEKIMTAIKIGTGKLGLLINDDETMQSADYLNYGKIPIFRGVIRGLETKR
RABV     749  LYELERISRNALSIYRAVEEGASKLGLIIKKEETMCSYDFLIYGKTPLFRGNILVPESKR
```

```
NiV(B)   909  MSRCCFWSETLVDETRSACSNISTTIA.......KAIENGLSRNVGYCINILKVIQQLLI
NiV(M)   909  MSRCCFWSETLVDETRSACSNISTTIA.......KAIENGLSRNVGYCINILKVIQQLLI
HeV      909  MSRCCFWSETLVDETRSACSNISTTIA.......KAIENGLSRNVGYCINVLKVIQQLLI
MojV     902  VSRCVFWSETLVDETRSACSNISTSIA.......KAIENGLSKDIGYSLNVLKTIQQIIV
LayV     902  VSRCVFWSETLVDETRSACSNISTSIA.......KAIENGMSKDVGYSLNILKTIQQIVV
GhV      917  ISRCVFWSETLVDETRSACSNISTAIT.......KSIENGIDHRFGFCLNYLKTMQQLSI
PIV3     850  LSRCVFWSETVIDETRSASSNLATSFA.......KAIENGYSPVLGYACSIFKNIQQLYI
SeV      850  LTRCVFWSETLVDENRSACSNISTSIA.......KAIENGYSPILGYCIALYKTCQQVCI
GSqRV    850  LTRCVFWSETLVDENRSACSNISTSIA.......KAIENGYSPILGYCIALYKTCQQVCI
MeV      850  IARCVFWSETIVDETRAACSNIATTMA.......KSIERGYDRYLAYSLNVLKVIQQILI
RDV      850  IARCVFWSETIVDETRAACSNIATTIA.......KSIERGFDRYLAYSLNILKVIQQILI
CDV      850  IARCVFWSETIVDETRAACSNISTTLA.......KAIEKGFDRYLAYTLNILKIIQQVLI
BPV      856  LSRVVFWSETIVDETRSACSNISTAIA.......KSIEQGFNRWIGYCLNILKVLQQLVI
BeV      833  LSRCVFWSETIVDETRSACSNICTSIA.......KSIEQGYSRWLGFAIAVLKTIQQLII
PMPV1    847  LSRCVFWSETVVDETRSACSNICTSIS.......KSIEQGYSRWIGYSIAMLKTIQQITI
MMLPV1   834  MSRVVFWSETIVDETRSACSNISTAVS.......KSIEQGYSRWIGYNINILKTIEQLVI
RuV      871  LSRAVFWSETIVDETRSACSNISTAIA.......KSIEQGFSRWIGYSINLLKTIEQIII
BaVV     859  LSRCVFWSETIVDETRSACSNIATTVA.......KAIEKGYSRDLGYALCILKTFQQIVI
ASPV     853  LTRCVFWSETLVDESRAACSNIATAVA.......KSIEQGYSPVVGYFFQVYKVFQQLMI
FLDV     845  ISRCVFWSETLVDETRAACSNISTSLA.......KAVENGISPLLCYLLNAWKTLQQLHI
APV      828  ASKLVLISGDLSENTVMSCSNISSTIA.......RLCENGLPKDFCYYLNYLMSCIQTYF
PPMV1    828  SSKLVLISGDLSENTVMSCANIASTVA.......RLCENGLPKDFCYYLNYLMSCVQTYF
NDV      828  SSKLVLISGDLSENTVMSCANIASTVA.......RLCENGLPKDFCYYLNYLMSCVQTYF
APV17    828  ASKLVLISGEIGENTPMSCSNLSSTIS.......RLCENGMPKDACYLLNYIMTCTQVLF
HPIV2    854  ASKLCLTADVLGECTQASCSNSATTIM.......RLTENGVEKDTCYKLNIYQSIRQLTY
MuV      856  VSKMCLTADILGDCSQASCSNLATTVM.......RLTENGVEKDLCYFLNAFMTIRQLCY
PIV5     850  ASKLCLTADVLGECTQSSCSNLATTVM.......RLTENGVEKDICFYLNIYMTIKQLSY
WTSPV    810  MTRMALWAETTVEETRSACSNLATVVS.......KALSQGANPKTMYFLHAIRVVEQILH
WHPV     803  AINMKLWGDKIVDEVRSGLSNIGSGCY.......KYIAQGGNIPRGLTIAKMRSFEQIMN
GPV      850  LSRCVFWSETVVDETRSACSNICTSAA.......KSIEQGFSRWVGYGICVLKTLQQLII
FPaV     841  ASRIVFWSETIVDETRSACSNISTAIA.......KAIEQGYSRWIGYCINFLKILEQLVV
HMPV     821  ILRVGPWINTILDDIKTSAESIGSLCQ.......ELEFRGESIIVSLILRNFWLYNLYMH
AMPV     821  ILRVGPWINTILDDIKTSAESIGSLCQ.......ELEFRGESLLVSLILRNFWLYELWMH
RSV      887  VLRVGPWINTILDDFKVSLESIGSLTQ.......ELEYRGESLLCSLIFRNVWLYNQIAL
MPV      832  ILRVGPWINTILDDIKTSTESIGSLTQ.......ELEYKGESLMSSLLLRNFWLYRLYSV
EBOV     819  ATRMAPLSDAIFDDLQGTLASIGTAFE.......RSISETRHIFPCRITAAFHTFFSVRI
VSV      794  WSRVTCVTND....QIPTCANIMSSVSTNALTVAHFAENPINAMIQYN..YFGTFARLLL
RABV     809  WARVSCVSND....QIVNLANIMSTVSTNALTVAQHSQSLIKPMRDFL..LMSVQAVFHY
```

```
NiV(B)    962  S....T......EFSINETLTLDVT.SPISNNLDWLITAALIPAPIGGFNYLNLSRIFVR
NiV(M)    962  S....T......EFSINETLTLDVT.SPISNNLDWLITAALIPAPIGGFNYLNLSRIFVR
HeV       962  S....T......EFSINETLTADVT.SPISNNLDWLVTASRIPAPIGGFNYLNLSRIFVR
MojV      955  S....L......KFSINETMTPDVT.NPLIQNINWLLTACLMPASLGGFNYLNLSRLYVR
LayV      955  S....L......KFSINETMTPDVT.NPLIQNVNWLLTACLMPASLGGFNYLNLSRLYVR
GhV       970  S....L......NFSINETITDDIK.MAFTNHPEWYKISSMIPAALGGFNYLNVSRLYVR
PIV3      903  A....L......GMNINPTITQNIK.DQYFRNPNWMQYASLIPASVGGFNYMAMSRCFVR
SeV       903  S....L......GMTINPTISPTVR.DQYFKGKNWLRCAVLIPANVGGFNYMSTSRCFVR
GSqRV     903  S....L......GMTINPTITTTVR.DQYFKGRNWLRCAVLIPANVGGFNYMSTSRCFVR
MeV       903  S....L......GFTINSTMTRDVV.IPLLTNNDLLIRMALLPAPIGGMNYLNMSRLFVR
RDV       903  S....L......GFTINSTMTQDVV.VPLLSNHDLLIRMALLPAPIGGMNYLNMSRLFVR
CDV       903  S....L......GFTINSAMTRDVI.EPLLQDHCLLTKMAILPAPIGGFNYLNMSRLFVR
BPV       909  S....L......KFTINPNMTDDIT.EPLIRNQNWLISAAIIPSQLGGFNYMNMCRLYVR
BeV       886  S....L......KYTINDSMTPDIV.QPIYRNPSWILSAALIPAQIGGFNYINLSRLFVR
PMPV1     900  A....L......QYTLNESMTRDIV.DPLIQNPNWIIAAALIPSQLGGFNYINMSRLYVR
MMLPV1    887  S....L......SFTINPSMTKDIS.QPILQSPYWLISAAIIPSQLGGFNYMNMCRLYVR
RuV       924  S....L......KFTLNDSMTEDVV.HPLYSNPNWILAAALVPSQLGGFNYMNMSRLYVR
BaVV      912  S....L......DYTINPYMNPEIR.NPIMGSPTWFIHAALTPAPLGGFNYLNMSRIYVR
ASPV      906  S....L......GFSVNPTLTKSVT.GPLIRSTEWLTVAAFVPASVGGFNYMAMARCFVR
FLDV      898  S....L......AFSINPTITKDLY.GPILSSQDWMIIAVIVPSQLGGFNYMSLSRLFVR
APV       881  D....K......EFSIFPYNEARRL.DQNKEDIKFVHTYVLTPAQVGGLSNLQYSRLYTR
PPMV1     881  D....S......EFSITHSSQPDSN.QSWIEDISFVHSYVLTPAQLGGLSNLQYSRLYTR
NDV       881  D....S......EFSITHSSQSDSN.QSWIEDISFVHSYVLTPAQLGGLSNLQYSRLYTR
APV17     881  D....N......SFSIIPSSSQSEI.STWLDNPAFLHAYALWPAQLGGLNNLQYTRLYTR
HPIV2     907  D....L......IFPQYSIPGETIS.EIFLQHPRLISRIVLLPSQLGGLNYLACSRLFNR
MuV       909  D....L......VFPQTKSLSQDIT.NAYLNHPILISRLCLLPSQLGGLNFLSCSRLFNR
PIV5      903  D....I......IFPQVSIPGDQIT.LEYINNPHLVSRLALLPSQLGGLNYLSCSRLFNR
WTSPV     863  G....I......HMTLNKGLENLKT.DGILEIPNFLALISRLPSHLGGFNYLLRDRLIVR
WHPV      856  G....A......NLSLNQYGSRDIE.MRLRGARGLLSELCSVPIQLGGYDYLPLSKCVMK
GPV       903  S....L......KYTINDSMTKDIT.DPLLLNLNWIIAAALLPSQLGGFNYINVSRLYVR
FPaV      894  S....L......KFTINPSMTQDVT.EPLMRNPYWIISAALLPSQLGGFNYLNMSRLYVR
HMPV      874  ESKQHPLAGKQLFKQLNKTLTSVQRFFEIKKENEVVDLWMNIPMQFGGGDPVVFYRSFYR
AMPV      874  ESKSHPLAGKQLYRQLSKTLAITQKFFGITKETDVVNLWMNVPMQFGGGDPVVLYRSFYR
RSV       940  QLKNHALCNNKLYLDILKVLKHLKTFFNLDNIDTALTLYMNLPMLFGGGDPNLLYRSFYR
MPV       885  DLKDHSLCGKQLYRSLIKVLKHLKRCFNLENLGECLELFLNVPMQFGGADPNVIYRSFYR
EBOV      872  LQHHHL......GFNKGFDLGQL.T.LGKPLDFGTISLALAVPQVLGGLSFLNPEKCFYR
VSV       848  MMHDPAL.....RQSLYEVQDK....IPGLHSSTFKYAMLYLDPSIGGVSGMSLSRFLIR
RABV      863  LLFSPIL.....KGRVYKILSA....E.GESFLLAMSRIIYLDPSLGGISGMSLGRFHIR

NiV(B)   1011  NIGDPVTASLADLKRMIDHSIMTE...............SVLQKVMNQEPGDA.......
NiV(M)   1011  NIGDPVTASLADLKRMIDHSIMTE...............SVLQKVMNQEPGDA.......
HeV      1011  NIGDPVTASLADLKRMIEHDLMTD...............KVLQKVMNQEPGDA.......
MojV     1004  NIGDPITASLADLKRMINSGLMTE...............RVLHQVMTQTPGDA.......
LayV     1004  NIGDPITASLADLKRMISSGLMTE...............RVLHQIMTQAPGDA.......
GhV      1019  NIGDVLVASLADIKRYISNNLLRD...............EIIQKIMTQVPGTA.......
PIV3      952  NIGDPSVAALADIKRFIKANLLDR...............SVLYRIMNQEPGES.......
SeV       952  NIGDPAVAALADLKRFIRADLLDK...............QVLYRIMNQEPGDS.......
GSqRV     952  NIGDPAVAALADLKRFIKAGLLDK...............QVLYRVMNQEPGDS.......
MeV       952  NIGDPVTSSIADLKRMILASLMPE...............ETLHQVMTQQPGDS.......
RDV       952  NIGDPVTSSIADLKRMIGASLMPE...............ETLHQVMTQQPGDS.......
CDV       952  NIGDPVTSSIADLKRMIRSGLLGV...............EILHQVMTQYPGDS.......
BPV       958  NIGDPVTASIADVKRMIKVNLLDE...............RIIQKIMHQDTGNS.......
BeV       935  NIGDPVTSSFADLKRYIQSGIISP...............SILQKIMKQECGSS.......
PMPV1     949  NIGDPVTASLADVKRMIKVGMLDE...............RILQRIMHQDKGTC.......
MMLPV1    936  NIGDPITASIADVKRMIKVKLLTP...............SILQKIMHQTPGDN.......
RuV       973  NIGDPVTSSMADIKRLIKSDLLHE...............SILQKIMNQEPGNG.......
BaVV      961  NIGDPVTASLADVKRMIKSKMLEP...............NMLQKIMNQSPGDS.......
ASPV      955  NIGDPATAAMADIKRMIEVDILTT...............EVLNKLLEQEPGDS.......
FLDV      947  NIGDPLVAALADVKRYIEIGLLTT...............AALTKFTTQVPGDS.......
APV       930  NIGDPGTTAFAELKRLETAKLIPK...............QVIINILTRPPGNG.......
PPMV1     930  NIGDPGTTAFAEVKRLEAVGLLSP...............SIMTNILTRPPGNG.......
NDV       930  NIGDPGTTAFAEVKRLEAVGLLSP...............SIMTNILTRPPGNG.......
APV17     930  NIGDPCTAALAEIKRLERAGLLPG...............KLMQNLLARKPSDG.......
HPIV2     956  NIGDPLGTAVADLKRLIKCGALES...............WILYNLLARKPGKG.......
MuV       958  NIGDPLVSAIADVKRLIKAGCLDI...............WVLYNILGRRPGKG.......
PIV5      952  NIGDPVVSAVADLKRLIKSGCMDY...............WILYNLLGRKPGNG.......
WTSPV     912  NIGDPLSVSFADLKGLLKAGFMTQ...............SVFNRIVNQKPGNG.......
WHPV      905  NPGDPLTTALAELKIKLKSRLLDP...............DVAVNFLNQPLEKP.......
GPV       952  NIGDPVTASLADLKRMIKVNLLDE...............RVLQKVMHQECGES.......
FPaV      943  NIGDPVTASIADVKRMIQVNLLSE...............RIIYKILYQPPGDC.......
HMPV      934  RTPDFLTEAISHVDILLRISANIR......NEAKISFF...KALLSIEKNERA.......
AMPV      934  RTPDFLTEAVSHMSVLLKVYGKAK......EGSKKDFF...KALLSVDKNKRA.......
RSV      1000  RTPDFLTEAIVHSVFILSYYTNHDLKDKLQDLSDDRLNKFLTCIITFDKNPNA.......
MPV       945  RTPDFLTESITHLILILKHFRRDL......EFNKDNVSKAVLSLLEFTKNDSA.......
EBOV      924  NLGDPVTSGLFQLKTYLRMIEMDD................LFLPLIAKNPGNC.......
VSV       899  AFPDPVTESLSFWRFIHVHA.RSE.................HLKEMSAVFGNPEIAKFRI
RABV      913  QFSDPVSEGLSFWREIWLSS.QES.................WIHALCQEAGNPDLGERTL
```

```
NiV(B)   1049  .SFLDWASDPYSGNLPDSQSITKTIKNITARTILRN...SPNPMLKGLFHDKSFDEDLEL
NiV(M)   1049  .SFLDWASDPYSGNLPDSQSITKTIKNITARTILRN...SPNPMLKGLFHDKSFDEDLEL
HeV      1049  .SFLDWASDPYSGNLPDSQSITKTIKNITARTILRT...SPNPMLKGLFHDKSFEEDLEL
MojV     1042  .SFLDWASDPYSANLPSSQSITKTIKNVTARLVLSN...AKNPILKGLFHNSYHEEDKSL
LayV     1042  .TFLDWASDPYSANLPSSQSITKTIKNVTARLVLAN...AKNPILKGLFHTTYHDEDRCL
GhV      1057  .SYLDWGSDPYSANIPHMQSITKMIKTITAKSILSN...SKNPLLKGLFHSKSHEEDNEL
PIV3      990  .SFLDWASDPYSCNLPQSQNITTMIKNITARNVLQD...SPNPLLSGLFTNTMIEEDEEL
SeV       990  .SFLDWASDPYSCNLPHSQSITTIIKNITARSVLQE...SPNPLLSGLFTETSGEEDLNL
GSqRV     990  .SFLDWASDPYSCNLPHSQSITTIIKNITARSVLQE...SPNPLLSGLFTDTSSEDDLNL
MeV       990  .SFLDWASDPYSANLVCVQSITRLLKNITARFVLIH...SPNPMLKGLFHDDSKEEDERL
RDV       990  .SFLDWASDPYSANLVCIQSITRLLKNITAKYVLIN...SPNPMLRGLFHDDSKEEDEQL
CDV       990  .SYLDWASDPYSANLPCVQSITRLLKNITARHVLIN...SPNPMLRGLFHDESQDEDEAL
BPV       996  .DYLDWASDPYSINTPSSQSVTVILKNITARTILSN...SDNPMLQGLFHFDFDEEDRDL
BeV       973  .TFLDWASDPYSVNIPHSQSIITMLKNVTSRTILQN...SVNPMLRGLFHFDFQQEDHDL
PMPV1     987  .TYLDWASDPYSINIPSSQSVTIMLKNITARTVLQH...SQNPMLHGLFHEDFDQEDQDL
MMLPV1    974  .TYLDWASDPYSVNIPHSQSISMMLKNVTSRMILQH...SDNPMLKGLFHFDFEQEDRDL
RuV      1011  .TFLDWSSDPYSINMPDSQSVTVILKNVTARHILSN...SPNPMLTGLFHLDFEQEDHDL
BaVV      999  .TYLDWANDPYSSNIPNTQSITKMIKNITARNILIH...SPNPMLKGLFHTGSIDEDHRL
ASPV      993  .TFLDWGSDPYSCNLPYSQSISAVIKNVTARSVLVD...SPNPLLQGLFDIDNQEEDEDL
FLDV      985  .TELDWASDPYSANLPHSQSVTTVIKHVTARSVLSK...SPNPMLEGLFHENTDEEDHDL
APV       968  .DWASLCNDPYSFNFETVSSPNIVLKKHTQRVLFET...CSNPLLSGVHTEDNEAEEKML
PPMV1     968  .DWASLCNDPYSFNFETVASPNIVLKKHTQKVLFET...CSNPLLSGVHTEDNEAEEKAL
NDV       968  .DWASLCNDPYSFNFETVASPSIVLKKHTQKVLFET...CSNPLLSGVHTEDNEAEEKAL
APV17     968  .TWASLCNDPYALNLDSAPSPNLVLKRHTQKVLFES...CTNPLLTGVYNEANDSEEAEL
HPIV2     994  .SWATLAADPYSLNQEYLYPPTTILKRHTQNTLMEI...CRNPMLKGVFTDNAKEEENLL
MuV       996  .KWSTLAADPYTLNIDYLVPSKTFLKKHAQYTLMER...SVNPMLRGVFSENAAEEEEEL
PIV5      990  .SWATLAADPYSINIEYQYPPTTALKRHTQQALMEL...STNPMLRGIFSDNAQAEENNL
WTSPV     950  .QWLQLGLDPYSGNIQSTQSMTTILERITSSSILAT...SVNPLLQGLFHDKYQEEDETT
WHPV      943  .TWRRLGLDPYGLNCKSTMSISSVLQRVTAKNILAR...SPNPMLKGLFAGNFEEEDEEM
GPV       990  .TFLDWASDPYSINIPSSQSVTIILKNITARLILQG...SRNPMLHGLFHDDFDQEDKDL
FPaV      981  .SYLDWASDPYSINIQNSQSVTTMLKNVTARSILQH...SDNPMLRGLFHFDFEQEDKDL
HMPV      978  .TLTTLMRDPQAVGSERQAKVTSDINRTAVTSILSL...SPNQLFSDSAI.HYSRNEEEV
AMPV      978  .TLTTLMRDPQAVGSERQARVTSEINRAAVTSVLSL...SPNQLFCDSAI.HYSRNEEEV
RSV      1053  .EFVTLMRDPQALGSERQAKITSEINRLAVTEVLST...APNKIFSKSAQ.HYTTTEIDL
MPV       992  .EFVTLMRDPQAIGSERQAKITSDINRTAVTSVLSN...APNEIFRTSAL.HYSSTENEL
EBOV      961  .TAIDFVLNPSGLNVPGSQDLTSFLRQIVRRTITLS...AKNKLINTLFHASADFEDEMV
VSV       941  THIDKLVEDPTSLNIAMGMSPANLLKTEVKKCLIESRQTIRNQVIKDATIYL.YHEEDRL
RABV      955  ESFTRLLEDPTTLNIRGGASPTILLKDAIRKALYDEVDKVENSEFREAILLS.KTHRDNF
```

```
NiV(B)   1105  ASFLMDRRVILPRAAHEILDNSLTGAREEIAGLLDTTKGLIRSGLRKSGIQPRLVSRLSH
NiV(M)   1105  ASFLMDRRVILPRAAHEILDNSLTGAREEIAGLLDTTKGLIRSGLRKSGLQPKLVSRLSH
HeV      1105  ATFLMDRRIILPRAAHEILDNSLTGAREEIAGLLDTTKGLIRSGLKKSGIQPKLVSRLSN
MojV     1098  AEFLMDRNIIMPRAAHEILDNSITGAREEISGMLDTTKGLIRTGLSKGGIQPRLVSRLSH
LayV     1098  AEFLMDRSIIMPRAAHEILDNSITGAREEISGMLDTTKGLIRTGLSKGGIQPRLVSKLSH
GhV      1113  AAFLMDRSIIIPRAAHEIIDNSVTGAREQIAGLLDTTKGLIRSGLRKGGIQPKTVDKIAN
PIV3     1046  AEFLMDRKVILPRVAHDILDNSLTGIRNAIAGMLDTTKSLIRVGINRGGLTYSLLRKISN
SeV      1046  ASFLMDRKVILPRVAHEILGNSLTGVREAIAGMLDTTKSLVRASVKKGGLSYGILRRLVN
GSqRV    1046  ASFLMDRKVILPRVAHEILGNSLTGVREAIAGMLDTTKSLVRASVKRGGLSYGILRRLIN
MeV      1046  AAFLMDRHIIVPRAAHEILDHSVTGARESIAGMLDTTKGLIRASMRKGGLTSRVITRLSN
RDV      1046  ATFLMDRSVIVPRAAHEILDHSITGAREAIAGMLDTTKGLIRTSSRRGGLTTRVIARLST
CDV      1046  AAFLMDRKIIIPRAAHEILDNTITGAREAIAGMLDTTKGLIRASMKRGGLTPRIITRLST
BPV      1052  ARFLLDRPVILPRAAHEIMDNSLTGARQEIAGMLDTTKGLIRNSIRLGAIRPKLADKLSL
BeV      1029  ADFLMDRPIIIPRAAHEIMDKSLTGARQEIAGMLDSTKGLIRNSLKTGGLRKSFINRLTL
PMPV1    1043  AKYLLDRPIIIPRAAHEIMDKSLTGARQEIAGMLDSTKGLIRNGLRAGGLRPRLIERLSF
MMLPV1   1030  ASFLLDRPIIIPRAAHEIMDKSLTGARQEIAGMLDSTKGLIRNSLKTGGLRPRLTEKLSM
RuV      1067  ATFLMDRPIIMPRAAHEIMSKTLTGARQEIAGMLDTTKGLIRNSLRLGGLRPKLVDRISL
BaVV     1055  AGFLMDRKVIIPRAAHEIMDNSIPGAREHIAGMLDTTKGLIRSGLRIGGLRSRLISKIAT
ASPV     1049  AMFLMDRGVIMPRVCHEILDNSLTGVRESIAGMLDTTKTLIRAGVRSGCLSLTMMRRLVN
FLDV     1041  ARFLIDREIILPRVANVILDQSVTGARSAIAGLLDTTKTLIKNTANKGGLSNRTVSKLLY
APV      1024  AEFLLNQESIHPRVAHAIMEASSVGRRKQIQGLVDTTNTVIKIALSRRPLGYKRLSRIIN
PPMV1    1024  AEFLLNQEAVHPRVAHAIMEASSVGRRKQIQGLVDTTNTVIKIALTRRPLGIKRLTRIIN
NDV      1024  AEFLLNQEVIHPRVAHAIMEASSVGRRKQIQGLVDTTNTVIKIALTRRPLGVKRLMRIIN
APV17    1024  AEFLLNQETIHPRVAHAILEASAVGRRKQIQGLLDTTNTIIKIALARKPLSLRKLRKVTN
HPIV2    1050  AKFLLDRDIVLPRVAHIIIDQSSIGRKKQIQGFFDTTRTIMRRSFEIKPLSTKKTLSVIE
MuV      1052  AQYLLDREVVMPRVAHVILAQSSCGRRKQIQGYLDSTRTIIRYSLEVRPLSAKKLNTVIE
PIV5     1046  ARFLLDREVIFPRVAHIIIEQTSVGRRKQIQGYLDSTRSIMSKSLEIKPLSNRKLNEILD
WTSPV    1006  AKKLLDVNRPTPRWASAILGLLPTGKRKKIAGYVETMKTTIRQACDRGGLTTELRDRLIN
WHPV      999  AKSLLDQARVYPRSAKAILDDTPTGERMKLCKMCDTTKTVIMQCSEKGGINMSDYTLIKR
GPV      1046  AKYLMDRPIIIPRAAHEIMDKCLTGARQEIAGMLDSTKGLIRNGLRAGGLRPRLVDKLSM
FPaV     1037  AEYLLNRSVIIPRAAHEIMDKSLTGARQEIAGMLDSTKGLIRNSFRLGGIRPRLIDRISC
HMPV     1033  GIIADNITPVYPHGLRVLYESLPFHKAEKVVNMISGTKSITNLLQRTSAINGEDIDRAVS
AMPV     1033  GLIAQNITPVYPHGLRVLYESLPFHKAEKVVNMISGTKSITNLLQRTSAINGEDIDRAVS
RSV      1108  NDIMQNIEPTYPHGLRVVYESLPFYKAEKIVNLISGTKSITNILEKTSAIDLTDIDRATE
MPV      1047  NGIASGVSPVYPHGLRVLYESLPFYKAEKIVNMVSGTKSITNILEKTSAISYTDIIRATN
EBOV     1017  CKWLLSSTPVMSRFAADIFSRTPSGKRLQILGYLEGTRTLLASKIINNNTETPVLDRLRK
VSV      1000  RSFLWSINPLFPRFLSEFKSGTFLGVADGLISLFQNSRTIRNSFKKKYHR..ELDDLIVR
RABV     1014  ILFLISVEPLFPRFLSELFSSSFLGIPESIIGLIQNSRTIRRQFRKSLSK..TLEESFYN
```

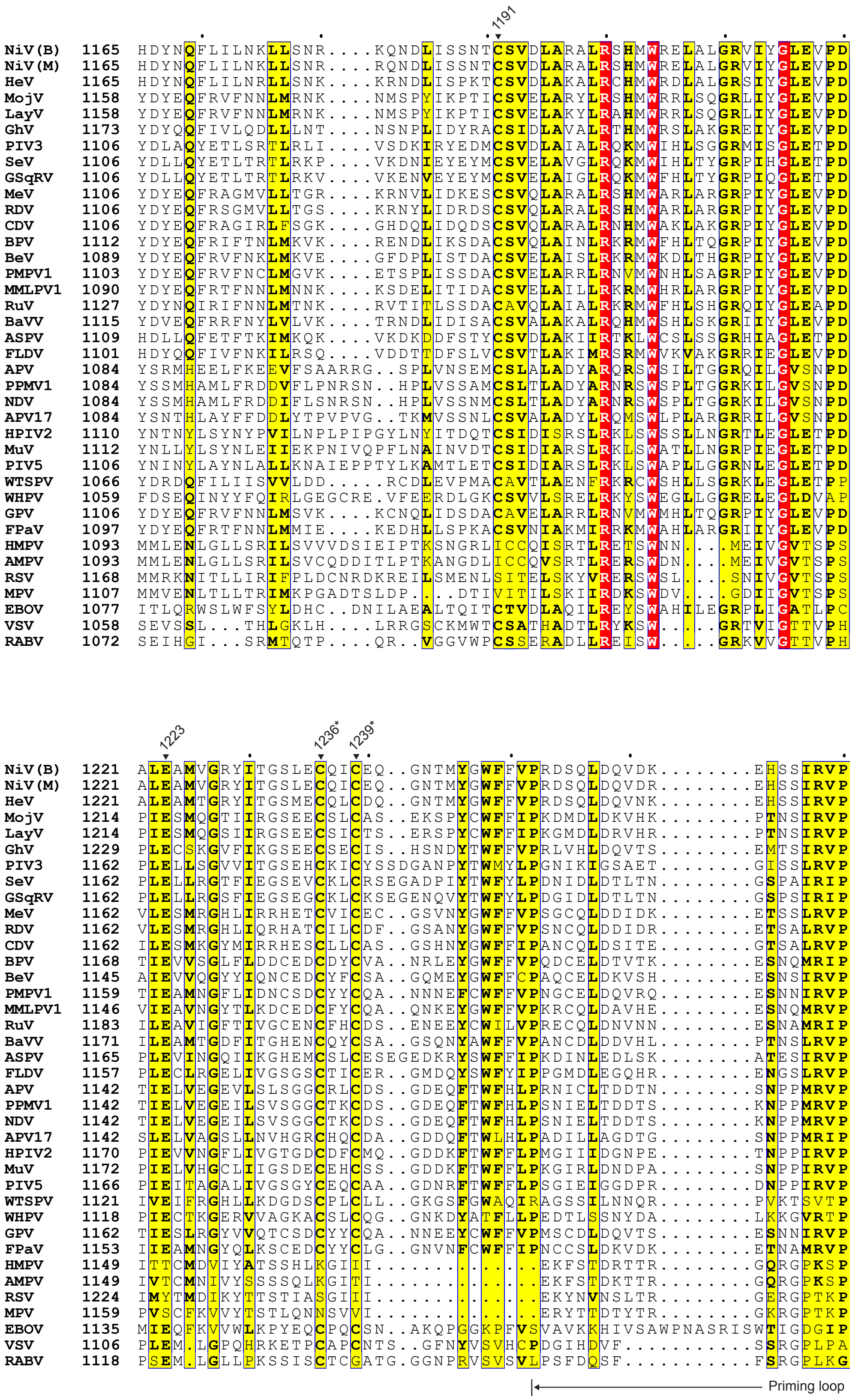
1191
NiV(B) 1165 HDYNQFLILNKLLSNR....KQNDLISSNTCSVDLARALRSHMWRELALGRVIYGLEVPD
NiV(M) 1165 HDYNQFLILNKLLSNR....RQNDLISSNTCSVDLARALRSHMWRELALGRVIYGLEVPD
HeV 1165 HDYNQFLILNRLLSNK....KRNDLISPKTCSVDLAKALRCHMWRDLALGRSIYGLEVPD
MojV 1158 YDYEQFRVFNNLMRNK....NMSPYIKPTICSVELARYLRSHMWRRLSQGRLIYGLEVPD
LayV 1158 YDYEQFRVFNNLMRNK....NMSPYIKPTICSVELAKYLRAHMWRRLSQGRLIYGLEVPD
GhV 1173 YDYQQFIVLQDLLLNT....NSNPLIDYRACSIDLAVALRTHMWRSLAKGREIYGLEVPD
PIV3 1106 YDLAQYETLSRTLRLI....VSDKIRYEDMCSVDLAIALRQKMWIHLSGGRMISGLETPD
SeV 1106 YDLLQYETLTRTLRKP....VKDNIEYEYMCSVELAVGLRQKMWIHLTYGRPIHGLETPD
GSqRV 1106 YDLLQYETLTRTLRKV....VKENVEYEYMCSVELAIGLRQKMWFHLTYGRPIQGLETPD
MeV 1106 YDYEQFRAGMVLLTGR....KRNVLIDKESCSVQLARALRSHMWARLARGRPIYGLEVPD
RDV 1106 YDYEQFRSGMVLLTGS....KRNYLIDRDSCSVQLARALRSHMWARLARGRPIYGLEVPD
CDV 1106 YDYEQFRAGIRLFSGK....GHDQLIDQDSCSVQLARALRNHMWAKLAKGRPIYGLEVPD
BPV 1112 YDYEQFRIFTNLMKVK....RENDLIKSDACSVQLAINLRKRMWFHLTQGRPIYGLEVPD
BeV 1089 YDYEQFRVFNKLMKVE....GFDPLISTDACSVELAISLRKRMWKDLTHGRPIYGLEVPD
PMPV1 1103 YDYEQFRVFNCLMGVK....ETSPLISSDACSVELARRLRNVMWNHLSAGRPIYGLEVPD
MMLPV1 1090 YDYEQFRTFNNLMNNK....KSDELITIDACSVELAILLRKRMWHRLARGRPIYGLEVPD
RuV 1127 YDYNQIRIFNNLMTNK....RVTITLSSDACAVQLAIALRKRMWFHLSHGRQIYGLEAPD
BaVV 1115 YDVEQFRRFNYLVLVK....TRNDLIDISACSVALAKALRQHMWSHLSKGRIIYGLEVPD
ASPV 1109 HDLLQFETFTKIMKQK....VKDKDDFSTYCSVDLAKIIRTKLWCSLSSGRHIAGLETPD
FLDV 1101 HDYQQFIVFNKILRSQ....VDDTTDFSLVCSVTLAKIMRSRMWVKVAKGRRIEGLEVPD
APV 1084 YSRMHEELFKEEVFSAARRG..SPLVNSEMCSLALADYARQRSWSILTGGRQILGVSNPD
PPMV1 1084 YSSMHAMLFRDDVFLPNRSN..HPLVSSAMCSLTLADYARNRSWSPLTGGRKILGVSNPD
NDV 1084 YSSMHAMLFRDDIFLSNRSN..HPLVSSNMCSLTLADYARNRSWSPLTGGRKILGVSNPD
APV17 1084 YSNTHLAYFFDDLYTPVPVG..TKMVSSNLCSVALADYLRQMSWLPLARGRRILGVSNPD
HPIV2 1110 YNTNYLSYNYPVILNPLPIPGYLNYITDQTCSIDISRSLRKLSWSSLLNGRTLEGLETPD
MuV 1112 YNLLYLSYNLEIIEKPNIVQPFLNAINVDTCSIDIARSLRKLSWATLLNGRPIEGLETPD
PIV5 1106 YNINYLAYNLALLKNAIEPPTYLKAMTLETCSIDIARSLRKLSWAPLLGGRNLEGLETPD
WTSPV 1066 YDRDQFILIISVVLDD.....RCDLEVPMACAVTLAENFRKRCWSHLLSGRKLEGLETPP
WHPV 1059 FDSEQINYYFQIRLGEGCRE.VFEERDLGKCSVVLSRELRKYSWEGLLGGRELEGLDVAP
GPV 1106 YDYEQFRVFNNLMSVK....KCNQLIDSDACAVELARRLRNVMWMHLTQGRPIYGLEVPD
FPaV 1097 YDYEQFRTFNNLMMIE....KEDHLLSPKACSVNIAKMIRRKMWAHLARGRIIYGLEVPD
HMPV 1093 MMLENLGLLSRILSVVVDSIEIPTKSNGRLICCQISRTLRETSWNN....MEIVGVTSPS
AMPV 1093 MMLENLGLLSRILSVCQDDITLPTKANGDLICCQVSRTLRERSWDN....MEIVGVTSPS
RSV 1168 MMRKNITLLIRIFPLDCNRDKREILSMENLSITELSKYVRERSWSL....SNIVGVTSPS
MPV 1107 MMVENLTLLTRIMKPGADTSLDP....DTIVITILSKIIRDKSWDV....GDIIGVTSPS
EBOV 1077 ITLQRWSLWFSYLDHC..DNILAEALTQITCTVDLAQILREYSWAHILEGRPLIGATLPC
VSV 1058 SEVSSL...THLGKLH....LRRGSCKMWTCSATHADTLRYKSW.....GRTVIGTTVPH
RABV 1072 SEIHGI...SRMTQTP....QR..VGGVWPCSSERADLLREISW.....GRKVVGTTVPH
1223
1236*
1239*
NiV(B) 1221 ALEAMVGRYITGSLECQICEQ..GNTMYGWFFVPRDSQLDQVDK........EHSSIRVP
NiV(M) 1221 ALEAMVGRYITGSLECQICEQ..GNTMYGWFFVPRDSQLDQVDR........EHSSIRVP
HeV 1221 ALEAMTGRYITGSMECQLCDQ..GNTMYGWFFVPRDSQLDQVNK........EHSSIRVP
MojV 1214 PIESMQGTIIRGSEECSLCAS..EKSPYCWFFIPKDMDLDKVHK........PTNSIRVP
LayV 1214 PIESMQGSIIRGSEECSICTS..ERSPYCWFFIPKGMDLDRVHR........PTNSIRVP
GhV 1229 PLECSKGVFIKGSEECSECIS..HSNDYTWFFVPRLVHLDQVTS........EMTSIRVP
PIV3 1162 PLELLSGVVITGSEHCKICYSSDGANPYTWMYLPGNIKIGSAET........GISSLRVP
SeV 1162 PLELLRGTFIEGSEVCKLCRSEGADPIYTWFYLPDNIDLDTLTN........GSPAIRIP
GSqRV 1162 PLELLRGSFIEGSEGCKLCKSEGENQVYTWFYLPDGIDLDTLTN........GSPSIRIP
MeV 1162 VLESMRGHLIRRHETCVICEC..GSVNYGWFFVPSGCQLDDIDK........ETSSLRVP
RDV 1162 VLESMRGHLIQRHATCILCDF..GSANYGWFFVPSNCQLDDIDR........ETSALRVP
CDV 1162 ILESMKGYMIRRHESCLLCAS..GSHNYGWFFIPANCQLDSITE........GTSALRVP
BPV 1168 TIEVVSGLFLDDCEDCDYCVA..NRLEYGWFFVPQDCELDTVTK........ESNQMRIP
BeV 1145 AIEVVQGYYIQNCEDCYFCSA..GQMEYGWFFCPAQCELDKVSH........ESNSIRVP
PMPV1 1159 TIEAMNGFLIDNCSDCYYCQA..NNNEFCWFFVPNGCELDQVRQ........ESNNIRVP
MMLPV1 1146 VIEAVNGYTLKDCEDCFYCQA..QNKEYGWFFVPKRCQLDAVHE........ESNQMRVP
RuV 1183 ILEAVIGFTIVGCENCFHCDS..ENEEYCWILVPRECQLDNVNN........ESNAMRIP
BaVV 1171 ILEAMTGDFITGHENCQYCSA..GSQNYAWFFVPANCDLDDVHL........PTNSLRVP
ASPV 1165 PLEVINGQIIKGHEMCSLCESEGEDKRYSWFFIPKDINLEDLSK........ATESLRVP
FLDV 1157 PLECLRGELIVGSGSCTICER..GMDQYSWFYIPPGMDLEGQHR........ENGSLRVP
APV 1142 TIELVEGEVLSLSGGCRLCDS..GDEQFTWFHLPRNICLTDDTN........SNPPMRVP
PPMV1 1142 TIELVEGEILSVSGGCTKCDS..GDEQFTWFHLPSNIELTDDTS........KNPPMRVP
NDV 1142 TIELVEGEILSVSGGCTKCDS..GDEQFTWFHLPSNIELTDDTS........KNPPMRVP
APV17 1142 SLELVAGSLLNVHGRCHQCDA..GDDQFTWLHLPADILLAGDTG........SNPPMRIP
HPIV2 1170 PIEVVNGFLIVGTGDCDFCMQ..GDDKFTWFFLPMGIIIDGNPE........TNPPIRVP
MuV 1172 PIELVHGCLIIGSDECEHCSS..GDDKFTWFFLPKGIRLDNDPA........SNPPIRVP
PIV5 1166 PIEITAGALIVGSGYCEQCAA..GDNRFTWFFLPSGIEIGGDPR........DNPPIRVP
WTSPV 1121 IVEIFRGHLLKDGDSCPLCLL..GKGSFGWAQIRAGSSILNNQR........PVKTSVTP
WHPV 1118 PIECTKGERVVAGKACSLCQG..GNKDYATFLLPEDTLSSNYDA........LKKGVRTP
GPV 1162 TIESLRGYVVQTCSDCYYCQA..NNEEYCWFFVPMSCDLDQVTS........ESNNIRVP
FPaV 1153 IIEAMNGYQLKSCEDCYYCLG..GNVNFCWFFIPNCCSLDKVDK........ETNAMRVP
HMPV 1149 ITTCMDVIYATSSHLKGIII..............EKFSTDRTTR........GQRGPKSP
AMPV 1149 IVTCMNIVYSSSSQLKGITI..............EKFSTDKTTR........GQRGPKSP
RSV 1224 IMYTMDIKYTTSTIASGIII..............EKYNVNSLTR........GERGPTKP
MPV 1159 PVSCFKVVYTSTLQNNSVVI..............ERYTTDTYTR........GKRGPTKP
EBOV 1135 MIEQFKVVWLKPYEQCPQCSN..AKQPGGKPFVSVAVKKHIVSAWPNASRISWTIGDGIP
VSV 1106 PLEM.LGPQHRKETPCAPCNTS..GFNYVSVHCPDGIHDVF...........SSRGPLPA
RABV 1118 PSEM.LGLLPKSSISCTCGATG.GGNPRVSVSVLPSFDQSF............FSRGPLKG
Priming loop

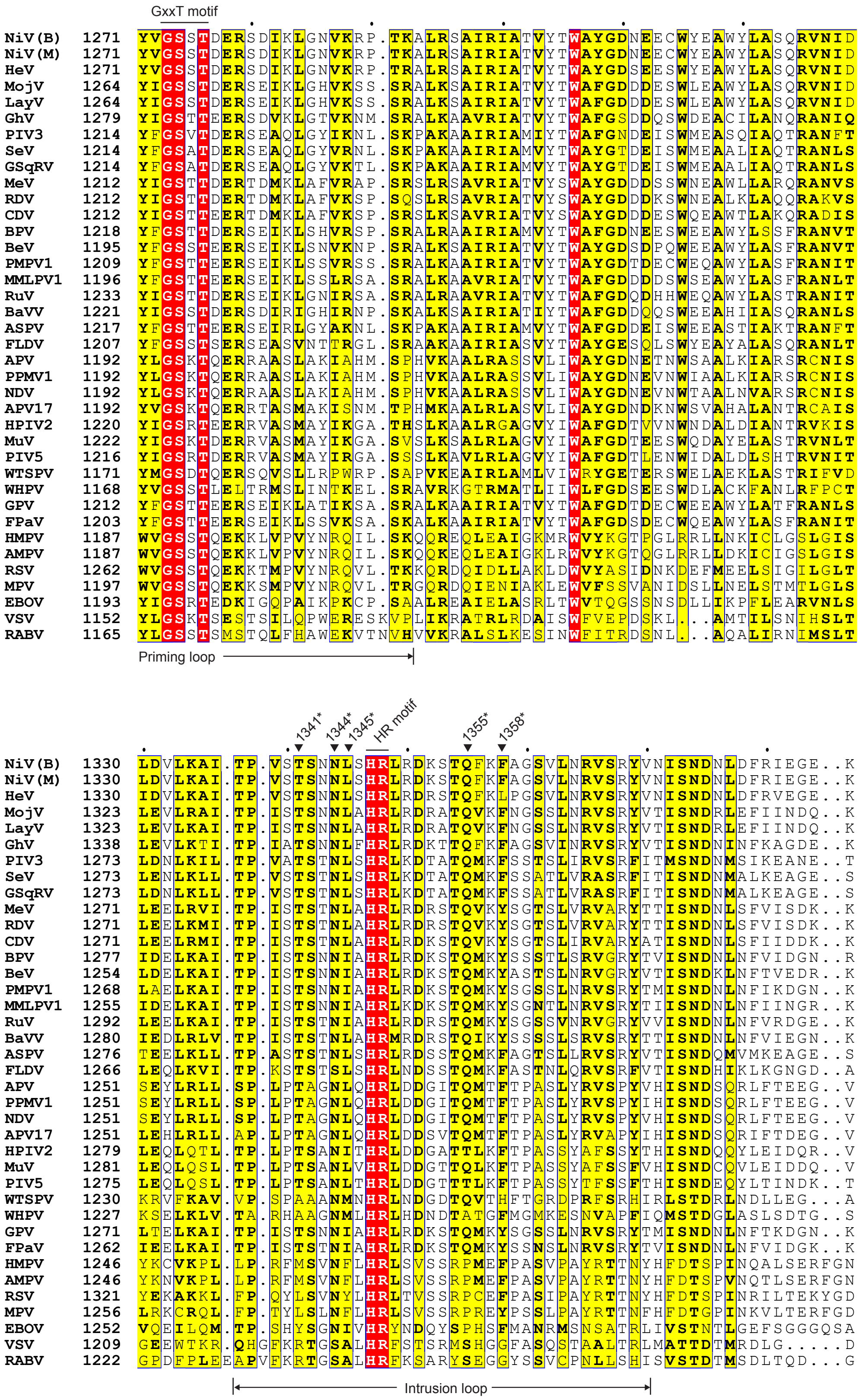

GxxT motif
NiV(B) 1271 YVGSSTDERSDIKLGNVKRP.TKALRSAIRIATVYTWAYGDNEECWYEAWYLASQRVNID
NiV(M) 1271 YVGSSTDERSDIKLGNVKRP.TKALRSAIRIATVYTWAYGDNEECWYEAWYLASQRVNID
HeV 1271 YVGSSTDERSDIKLGNVKRP.TRALRSAIRIATVYTWAYGDSEESWYEAWYLASQRVNID
MojV 1264 YIGSSTDERSEIKLGHVKSS.SRALKSAIRIATVYTWAFGDDDESWLEAWYLASQRVNID
LayV 1264 YIGSSTDERSEIKLGHVKSS.SRALKSAIRIATVYTWAFGDDDESWLEAWYLASQRVNID
GhV 1279 YIGSTTEERSDVKLGTVKNM.SRALKAAVRIATVYTWAFGSDDQSWDEACILANQRANIQ
PIV3 1214 YFGSVTDERSEAQLGYIKNL.SKPAKAAIRIAMIYTWAFGNDEISWMEASQIAQTRANFT
SeV 1214 YFGSATDERSEAQLGYVRNL.SKPAKAAIRIAMVYTWAYGTDEISWMEAALIAQTRANLS
GSqRV 1214 YFGSATDERSEAQLGYVKTL.SKPAKAAIRIAMVYTWAYGTDEISWMEAALIAQTRANLS
MeV 1212 YIGSTTDERTDMKLAFVRAP.SRSLRSAVRIATVYSWAYGDDDSSWNEAWLLARQRANVS
RDV 1212 YIGSTTDERTDMKLAFVKSP.SQSLRSAVRIATVYSWAYGDDDKSWNEALKLAQQRAKVS
CDV 1212 YIGSTTEERTDMKLAFVKSP.SRSLKSAVRIATVYSWAYGDDDESWQEAWTLAKQRADIS
BPV 1218 YFGSTTDERSEIKLSHVRSP.SRALRAAIRIAMVYTWAFGDNEESWEEAWYLSSFRANVT
BeV 1195 YFGSSTEERSEIKLSNVKNP.SRALKSAIRIATVYTWAYGDSDPQWEEAWYLASFRANVT
PMPV1 1209 YFGSTTEERSEIKLSSVRSS.SRALKSAIRIATVYTWAYGDTDECWEQAWYLASFRANIT
MMLPV1 1196 YFGSTTEERSEIKLSSLRSA.SRALKAAVRIATVYTWAFGDDDDSWSEAWYLASFRANLT
RuV 1233 YIGSTTDERSEIKLGNIRSA.SRALRAAVRIATVYTWAFGDQDHHWEQAWYLASTRANIT
BaVV 1221 YIGSSTDERSDIRIGHIRNP.SKALKSAIRIATIYTWAFGDDQQSWEEAHIIASQRANLS
ASPV 1217 YFGSTTEERSEIRLGYAKNL.SKPAKAAIRIAMVYTWAFGDDEISWEEASTIAKTRANFT
FLDV 1207 YFGSSTSERSEASVNTTRGL.SRAAKAAIRIASVYTWAYGESQLSWYEAYALASQRANLT
APV 1192 YLGSKTQERRAASLAKIAHM.SPHVKAALRASSVLIWAYGDNETNWSAALKIARSRCNIS
PPMV1 1192 YLGSKTQERRAASLAKIAHM.SPHVKAALRASSVLIWAYGDNEVNWIAALKIARSRCNIS
NDV 1192 YLGSKTQERRAASLAKIAHM.SPHVKAALRASSVLIWAYGDNEVNWTAALNVARSRCNIS
APV17 1192 YVGSKTQERRTASMAKISNM.TPHMKAALRLASVLIWAYGDNDKNWSVAHALANTRCAIS
HPIV2 1220 YIGSRTEERRVASMAYIKGA.THSLKAALRGAGVYIWAFGDTVVNWNDALDIANTRVKIS
MuV 1222 YIGSKTDERRVASMAYIKGA.SVSLKSALRLAGVYIWAFGDTEESWQDAYELASTRVNLT
PIV5 1216 YIGSRTDERRVASMAYIRGA.SSSLKAVLRLAGVYIWAFGDTLENWIDALDLSHTRVNIT
WTSPV 1171 YMGSDTQERSQVSLLRPWRP.SAPVKEAIRLAMLVIWRYGETERSWELAEKLASTRIFVD
WHPV 1168 YVGSSTLELTRMSLINTKEL.SRAVRKGTRMATLIIWLFGDSEESWDLACKFANLRFPCT
GPV 1212 YFGSTTEERSEIKLATIKSA.SRALKAAIRIATVYTWAFGDTDECWEEAWYLATFRANLS
FPaV 1203 YFGSTTEERSEIKLSSVKSA.SKALKAAIRIATVYTWAFGDSDECWQEAWYLASFRANIT
HMPV 1187 WVGSSTQEKKLVPVYNRQIL.SKQQREQLEAIGKMRWVYKGTPGLRRLLNKICLGSLGIS
AMPV 1187 WVGSSTQEKKLVPVYNRQIL.SKQQKEQLEAIGKLRWVYKGTQGLRRLLDKICIGSLGIS
RSV 1262 WVGSSTQEKKTMPVYNRQVL.TKKQRDQIDLLAKLDWVYASIDNKDEFMEELSIGILGLT
MPV 1197 WVGSSTQEKKSMPVYNRQVL.TRGQRDQIENIAKLEWVFSSVANIDSLLNELSTMTLGLS
EBOV 1193 YIGSRTEDKIGQPAIKPKCP.SAALREAIELASRLTWVTQGSSNSDLLIKPFLEARVNLS
VSV 1152 YLGSKTSESTSILQPWERESKVPLIKRATRLRDAISWFVEPDSKL...AMTILSNIHSLT
RABV 1165 YLGSSTSMSTQLFHAWEKVTNVHVVKRALSLKESINWFITRDSNL...AQALIRNIMSLT
Priming loop
1341*
1344*
1345*
HR motif
1355*
1358*
NiV(B) 1330 LDVLKAI.TP.VSTSNNLSHRLRDKSTQFKFAGSVLNRVSRYVNISNDNLDFRIEGE..K
NiV(M) 1330 LDVLKAI.TP.VSTSNNLSHRLRDKSTQFKFAGSVLNRVSRYVNISNDNLDFRIEGE..K
HeV 1330 IDVLKAI.TP.VSTSNNLSHRLRDRSTQFKLPGSVLNRVSRYVNISNDNLDFRVEGE..K
MojV 1323 LEVLRAI.TP.ISTSNNLAHRLRDRATQVKFNGSSLNRVSRYVTISNDRLEFIINDQ..K
LayV 1323 LEVLKAI.TP.ISTSNNLAHRLRDRATQVKFNGSSLNRVSRYVTISNDRLEFIINDQ..K
GhV 1338 LEVLKTI.TP.IATSNNLFHRLRDKTTQFKFAGSVINRVSRYVTISNDNINFKAGDE..K
PIV3 1273 LDNLKIL.TP.VATSTNLSHRLKDTATQMKFSSTSLIRVSRFITMSNDNMSIKEANE..T
SeV 1273 LENLKLL.TP.VSTSTNLSHRLKDTATQMKFSSATLVRASRFITISNDNMALKEAGE..S
GSqRV 1273 LDNLKLL.TP.VSTSTNLSHRLKDTATQMKFSSATLVRASRFITISNDNMALKEAGE..S
MeV 1271 LEELRVI.TP.ISTSTNLAHRLRDRSTQVKYSGTSLVRVARYTTISNDNLSFVISDK..K
RDV 1271 LEELKMI.TP.ISTSTNLAHRLRDRSTQVKYSGTSLVRVARYTTISNDNLSFVISDK..K
CDV 1271 LEELRMI.TP.ISTSTNLAHRLRDKSTQVKYSGTSLIRVARYATISNDNLSFIIDDK..K
BPV 1277 IDELKAI.TP.ISTSNNIAHRLRDKSTQMKYSSTSLSRVGRYTVISNDNLNFVIDGN..R
BeV 1254 LDELKAI.TP.ISTSNNIAHRLRDKSTQMKYASTSLNRVGRYVTISNDKLNFTVEDR..K
PMPV1 1268 LAELKAI.TP.ISTSNNIAHRLRDKSTQMKYSGSSLNRVSRYTMISNDNLNFIKDGN..K
MMLPV1 1255 IDELKAI.TP.ISTSNNIAHRLRDKSTQMKYSGNTLNRVSRYTIISNDNLNFIINGR..K
RuV 1292 LEELKAI.TP.ISTSTNIAHRLRDRSTQMKYSGSSVNRVGRYVVISNDNLNFVRDGE..K
BaVV 1280 IEDLRLV.TP.ISTSTNLAHRMRDRSTQIKYSSSSLSRVSRYTTISNDNLNFVIEGN..K
ASPV 1276 TEELKLL.TP.ASTSTNLSHRLRDSSTQMKFAGTSLLRVSRYVTISNDQMVMKEAGE..S
FLDV 1266 LEQLKVI.TP.KSTSTSLSHRLNDSSTQMKFASTNLQRVSRFVTISNDHIKLKGNGD..A
APV 1251 SEYLRLL.SP.LPTAGNLQHRLDDGITQMTFTPASLYRVSPYVHISNDSQRLFTEEG..V
PPMV1 1251 SEYLRLL.SP.LPTAGNLQHRLDDGITQMTFTPASLYRVSPYIHISNDSQRLFTEEG..V
NDV 1251 SEYLRLL.SP.LPTAGNLQHRLDDGITQMTFTPASLYRVSPYVHISNDSQRLFTEEG..V
APV17 1251 LEHLRLL.AP.LPTAGNLQHRLDDSVTQMTFTPASLYRVAPYIHISNDSQRIFTDEG..V
HPIV2 1279 LEQLQTL.TP.LPTSANITHRLDDGATTLKFTPASSYAFSSYTHISNDQQYLEIDQR..V
MuV 1281 LEQLQSL.TP.LPTSANLVHRLDDGTTQLKFTPASSYAFSSFVHISNDCQVLEIDDQ..V
PIV5 1275 LEQLQSL.TP.LPTSANLTHRLDDGTTTLKFTPASSYTFSSFTHISNDEQYLTINDK..T
WTSPV 1230 KRVFKAV.VP.SPAAANMNHRLNDGDTQVTHFTGRDPRFSRHIRLSTDRLNDLLEG..A
WHPV 1227 KSELKLV.TA.RHAAGNMLHRLHDNDTATGFMGMKESNVAPFIQMSTDGLASLSDTG..S
GPV 1271 LTELKAI.TP.ISTSNNIAHRLRDKSTQMKYSGSSLNRVSRYTMISNDNLNFTKDGN..K
FPaV 1262 IEELKAI.TP.ISTSTNIAHRLRDKSTQMKYSSNSLNRVSRYTVISNDNLNFVIDGK..K
HMPV 1246 YKCVKPL.LP.RFMSVNFLHRLSVSSRPMEFPASVPAYRTTNYHFDTSPINQALSERFGN
AMPV 1246 YKNVKPL.LP.RFMSVNFLHRLSVSSRPMEFPASVPAYRTTNYHFDTSPVNQTLSERFGN
RSV 1321 YEKAKKL.FP.QYLSVNYLHRLTVSSRPCEFPASIPAYRTTNYHFDTSPINRILTEKYGD
MPV 1256 LRKCRQL.FP.TYLSLNFLHRLSVSSRPREYPSSLPAYRTTNFHFDTGPINKVLTERFGD
EBOV 1252 VQEILQM.TP.SHYSGNIVHRYNDQYSPHSFMANRMSNSATRLIVSTNTLGEFSGGGQSA
VSV 1209 GEEWTKR.QHGFKRTGSALHRFSTSRMSHGGFASQSTAALTRLMATTDTMRDLG.....D
RABV 1222 GPDFPLEEAPVFKRTGSALHRFKSARYSEGGYSSVCPNLLSHISVSTDTMSDLTQD...G
Intrusion loop

```
                                                            1421 1423      1428* 1429*
NiV(B)  1386  VDTNLIYQQAMLLGLSVLEGKFRLR.LETDDYNGIYHLHVKDNCCVKEVADVGQ..VDAE
NiV(M)  1386  VDTNLIYQQAMLLGLSVLEGKFRLR.LETDDYNGIYHLHVKDNCCVKEVADVGQ..VDAE
HeV     1386  VDTNLIYQQTMLLGLSVLEGKFRLR.TETDDYNGIYHLHVRDNCCVKEVADIGG..VNAE
MojV    1379  VDTNLIFQQVMLLGLSAIEQRYRYK.TCTGKENVIYHLHARENCCVIEMSSTGN..VPSL
LayV    1379  VDTNLIFQQVMLLGLSAIEQRYRYR.VQTGDENVIYHLHAKEHCCIIEMSSTGN..VPSL
GhV     1394  QDTNFIFQQSMLLGLSHLEKVYKNY.ARTGSESIVYHLHVESYCCIRPTKEIPY..CHSL
PIV3    1329  KDTNLIYQQIMLTGLSVFEYLFRLK.ETTGHNPIVMHLHIEDECCIKESFNDEH..INPE
SeV     1329  KDTNLVYQQIMLTGLSLFEFNMRYK.KGSLGKPLILHLHLNNGCCIMESPQEAN..IPPR
GSqRV   1329  KDTNLVYQQIMLTGLSVFEFNVRYK.LGPLERPLILHLHLNNGCCIMESPQEIS..IPPR
MeV     1327  VDTNFIYQQGMLLGLGVLETLFRLE.KDTGSSNTVLHLHVETDCCVIPMIDHPR..IPSS
RDV     1327  VDTNFIYQQGMLLGLGVLETLFRLD.KDTGPSNTVLHLHVETDCCVIPMVDHPR..IPSL
CDV     1327  VDTNFIYQQGMLLGLGILEHLFRLS.STTGDSNTVLHLHVETDCCVIPMSDHPR..VPGL
BPV     1333  VDTNLIYQQIMLMGLSILEDHFRFL.KETGPKNTLLHLHINTPCCIVQMTEHPY..ISSD
BeV     1310  IDTNLIYQQIMLIGLSILEEHFRYT.SMTDEYNTVLHLHINKHCCLKEMCDHPY..VDTE
PMPV1   1324  IDTNLIYQQVMLLGLASLEDLFRFQ.LSTGTTNTVYHLHVEQNCCVIEMEDHPY..VDVT
MMLPV1  1311  VDTNLVYQQIMLLGLSILEGKFRFK.SDTGDSNTVLHLHIVKNCCVIEMEDHPY..TNSL
RuV     1348  IDTNLVYQQIMLLGISVLEEQYRYF.IDTGPTNRLLHLHIVKNCCVIEMPDHPY..IDTD
BaVV    1336  TDTNYIYQQGMLLGLASIEDRFRFN.QSTGSHNTVYHLHTIDECCVIKMVDYQN..VVSL
ASPV    1332  KDTNLVYQQVMLIGLSFLEYLSRHE.STTGDENTLLHLHLNPGCCIVTNEDEEK..NPST
FLDV    1322  GDTNLIYQQVMLVGLSIIELTHRRQ.ENTGEDSKVLHLHIREDCCVEFTQDQGD..IPSL
APV     1307  KEGNVVYQQIMLLGLSLLESLFPLT.TTRIFDEVTLHLHSKFSCCIREAPVSVP..FELQ
PPMV1   1307  KEGNVVYQQIMLLGLSLIESLFPMT.TTKTYDEITLHLHSKFSCCIREAPVAVP..FELL
NDV     1307  KEGNVVYQQIMLLGLSLIESLFPMT.TTRTYDEITLHLHSKFSCCIREAPVAVP..FELL
APV17   1307  KESNIIYQQLMLLGLSAIESLFPLT.ANKVFEEVTLHLHTKFSCCIREAPVSIP..FEMQ
HPIV2   1335  VDSNIIYQQLMITGLGIIETYHNPP.IRTSTQEITLHLHTSSSCCVRSVDGCLI..CESN
MuV     1337  TDSNLIYQQVMITGLALIETWNNPP.INFSVYETTLHLHTGSSCCIRPVESCVV..NPPL
PIV5    1331  ADSNIIYQQLMITGLGILETWNNPP.INRTFEESTLHLHTGASCCVRPVDSCII..SEAL
WTSPV   1285  EDTNFVFQPAMLTCIATLEDELRYT.TQTA.ADLTYHLHVETTCCIREVPNAIDPEVPDI
WHPV    1283  EDTNFIYQNALHIGIFMIEDSVGKEVTRYGNNEELYHLHVETGCCVKSQVYTPLREL.GK
GPV     1327  IDTNLIYQQTMLIGLSALEDLFRFK.KTTGVENTVYHLHVEQNCCVIEMEDHPY..IDTH
FPaV    1318  IDTNLIYQQVMLLGLSSLEDLFRLR.LSTGIENTVYHLHVESNCCVVEMSDHPY..IESN
HMPV    1304  EDINLVFQNAISCGISIMSVVEQLT..GRSPKQLVLIPQLE.......EIDIMPPPVFQGK
AMPV    1304  EDINLVFQNAISCGISVMSVVEQLT..GRSPKQLVMIPQLE.......EIDIMPPPVFLGK
RSV     1379  EDIDIVFQNCISFGLSLMSVVEQFT..NVCPNRIILIPKLN.......EIHLMKPPIFTGD
MPV     1314  EDINLVFQNAISYGLSTMSLVEQFT..GVCPNKVLLVPKLQ.......EIQLMKVPIFQGG
EBOV    1310  RDSNIIFQNVINYAVALFDIKFRNT.EATDIQYNRAHLHL.TKCCTREVPAQYL..TYTS
VSV     1263  QNFDFLFQATLLYAQITT..TVAR...DGWITSCTDHYHIACKSCLRPIEEITLD.....
RABV    1279  KNYDFMFQPLMLYAQTWTSELVQR...DTRLRDSTFHWHLRCNRCVRPIDDVTLE.....
```

```
NiV(B)  1443  LPIPEYTEVDNNHLIYDPDPVSEIDCSRLSN........QESKSRE...........LDF
NiV(M)  1443  LPIPEYTEVDNNHLIYDPDPVSEIDCSRLSN........QESKSRE...........LDF
HeV     1443  LPVPEYTEVENNRLIYDPDPVSEIDCDRLSK........QESKARE...........LDF
MojV    1436  LEPPFYKEIRENRLIYDDNPIIDKDLRKLRT........QAWYASD...........VDF
LayV    1436  IEPPIYKEIKENRLIYDDNPIIDKDLKKLRT........QAWYSSD...........VDF
GhV     1451  RPVPLIEHSSENVLIYDPSPVTDYQKIKLEI........QYLDKEE...........LDF
PIV3    1386  STLELIRYPESNEFIYDKDPLKDVDLSRLMV........IKDHSYT...........IDM
SeV     1386  STLDLEITQENNKLIYDPDPLKDVDLELFSK........VRDVVHT...........VDM
GSqRV   1386  STLDLEITQESNRLIYDPDPLRGIDLELFNK........VRDVVHT...........VDM
MeV     1384  RKLELRAELCTNPLIYDNAPLIDRDATRLYT........QSHRRHL...........VEF
RDV     1384  HELKFRRELCTNPLIYDSAPIIDREATKLYT........QSHRRHL...........VEF
CDV     1384  RKVVIPRNICTNPLIYDSNPIIEKDAVRLYN........QSHRKHI...........VEF
BPV     1390  VILPELQHVQANKLIYDDNPIIDKDQLKIHN........QLFRSSD...........LDF
BeV     1367  KTMPTLRRVETNKLIYDPDPIIESNKNTIQQ........QVFRKGM...........LDF
PMPV1   1381  EPPPVLQGVYTNRLIYDDNPLIEKDVERIYT........QTYRSVT...........IDF
MMLPV1  1368  LPVPNLVKVECNRLIYDDDPILERDTEQIHR........QLYNLPS...........LDF
RuV     1405  IVLPSLQFVYGNKLIYDDQPIIDKDKMILDQ........QIFKQGT...........LFF
BaVV    1393  GECPIIIPPKNNKLIYDDNPIIERDQLRLDQ........QRYRVAL...........VDY
ASPV    1389  VEVEIKTRTDANVMIYDPEPVSKDDLKKLQM........IKTEIHE...........VDF
FLDV    1379  NNLPEFEDHPTNRLIYDPNPLDNIYITRIKE........TSVYLHE...........LDF
APV     1364  GYLPDLRTVASNKFMFDPQPITDTDFGRLNL........TIFRSYE...........LNL
PPMV1   1364  GMAPELRTVTSNKFMYDPSPISERDFARLDL........AIFKSYE...........LNL
NDV     1364  GLAPELRMVTSNKFMYDPSPISERDFARLDL........AIFKSYE...........LNL
APV17   1364  GEIPRIRNGEGNKFVYDSCPLPSPEATILDV........KTFKNYE...........LDL
HPIV2   1392  GEVPQITVPYTNTFVYDPDPLADYEIAHLDY........LSYQAKI...........GST
MuV     1394  LPVPFINVPQMNKFVYDPEPLSLLEMEKIED........IAYQTRI...........GGL
PIV5    1388  TVKPHITVPYSNKFVFDEDPLSEYETAKLES........LSFQAQL...........GNI
WTSPV   1343  DLSTHTDTLRTNPLIYDPQGVQFSPLTDQDSL.......TEIKSPA...........LTA
WHPV    1342  LEIEIPAPLKENKFIYDSGGLTSIVGKFEEI........REIQESG...........IEL
GPV     1384  EPPPVLRAVTSNRLIYDDQPLRDGEIENIYS........QNYTSNI...........LDF
FPaV    1375  NILPVLQKVNCNKLIYDDNPIIEKDTIKIDQ........QNYRKGM...........LDF
HMPV    1356  FNYKLVDKITSDQHIFSPDKIDMLTLGKMLMPT......IK......GQKT.DQFLNKRE
AMPV    1356  FDYKLVEKISSDQHIFSPDKLDLVTLGKMLMPS......TS......GAKS.DQFWNRKE
RSV     1431  VDIHKLKQVIQKQHMFLPDKISLTQYVELFLSN......KTLKSGSHVNSN.LILAHKIS
MPV     1366  FNLQSIIPIIRQQHMFLPNHITPAQYIELFLSS......KQFHSRINLKHNNRFKLVLQK
EBOV    1366  TLDLDLTRYRENELIYDNNPLKGGLNCNISFDN......PFFQGKR...........LNI
VSV     1313  ...........SSMDYTPPDVSHVLKTWRNGEGSWGQEIKQIYPLE...........GNW
RABV    1331  ...........TSQIFEFPDVSKRISRMVSGAVPHFQRLPDIRLRP...........GDF
```

```
NiV(B)   1484  PLWSTEELHDALAKTVAQTV.LEIITKADKDVLKQ........HLAIDSDDNINSLITEF
NiV(M)   1484  PLWSTEELHDVLAKTVAQTV.LEIITKADKDVLKQ........HLAIDSDDNINSLITEF
HeV      1484  PLWSTEELHDVLAKTVAQTV.LEIITKADKDVLKQ........HLAIDSDDSINSLITEF
MojV     1477  SNWKSSDLHCVLAQSLAHTI.IDSISKSDNDVLKQ........FVTISSDDNINSLITEF
LayV     1477  SNWKSSDLHCVLAQSLAHTI.IESISKSDNDILKQ........FVTISSDDNINSLITEF
GhV      1492  TVWSTLELQELLAESTAYTI.IDIITKSERDHLKE........FVSVSSDDNISSLITEF
PIV3     1427  NYWDDTDIIHAISICTAITI.ADTMSQLDRDNLKE........IIVIANDDDINSLITEF
SeV      1427  TYWSDDEVIRATSICTAMTI.ADTMSQLDRDNLKE........MIALVNDDDVNSLITEF
GSqRV    1427  TYWSDDEVIRAASICTAMTI.ADTMSQLDRDNLKE........MIALANDDDINSLITEF
MeV      1425  VTWSTPQLYHILAKSTALSM.IDLVTKFEKDHMNE........ISALIGDDDINSFITEF
RDV      1425  VTWSTSQLYHILAKSTALSM.IDLVTKFEKDHMNE........VSALIGDDDINSFITEF
CDV      1425  VTWTTGQLYHVLAKSTAMSM.VEMITKFEKDHLNE........VTALIGDDDINSFITEF
BPV      1431  PNWTLSELNISLSQSLAMTI.IEIITKENKDHLSE........FKVLVLDDDFNSLITEF
BeV      1408  PVWPLRNLNTALAQSLAGTI.IEIISKENRDHLNE........FKTLENDDDINSMITEF
PMPV1    1422  PRYSMNELNTVLAQSLALTI.VDIITKETKDHLTE........FKVLANDDDINSLITEF
MMLPV1   1409  PRWTITELNEGLAQSLAMTI.VDIITKENKDHLTE........FKVFANDDDTNSLITEF
RuV      1446  PKWTIRELDELLAQSLAHTF.VEIITQEDKDHLTE........LKVLNNDDDINSLITEC
BaVV     1434  TMWSSHELSQGLAQSLALTL.IESITKSENDHLNE........IKSVEGDDDIKSLITEF
ASPV     1430  SLWDNSSILGALNIATAQTV.VDTISQIDRDDLKE........VVAIASDDSINSLITEF
FLDV     1420  LAWHDEDIKTVLGNSVGQMI.EEVIATTERDNLKD........INALSNEDNINSIITEM
APV      1405  DSYPTIELMNVLAIASGKLI.GQSVVSYDEDTSIK........NDAILVYDNTRNWISEA
PPMV1    1405  ESYSTMELMNILSISSGKLI.GQSVVSYDEDTSIK........NDAIIVYDNTRNWISEA
NDV      1405  ESYSTLELMNILSISSGKLI.GQSVVSYDEDTSIK........NDATIVYDNTRNWISEA
APV17    1405  DHYTTGELMQVLELACGKLI.GQSVVSYNEDTSIK........NDAIIVYDNTRNWISEA
HPIV2    1433  DYYSLTDKIDLLAHLTAKQM.INSIIGLDETVSIV........NDAVILSDYTNNWISEC
MuV      1435  DQIPLLEKIPLLAHLTAKQM.VNSITGLDEATSIV........NDAVVQADYTSNWISEC
PIV5     1429  DAVDMTGKLTLLSQFTARQI.INAITGLDESVSLT........NDAIVASDYVSNWISEC
WTSPV    1385  KTQSNQSLTRGLGVCVGTYV.GKGITGLAKMAALGEN......WTDTEGSLTVTGLISEI
WHPV     1383  EDMDSDSLSKLMGLTVGKFIMGNIITGSRKREFGG........LADQGTEEDVGGLLSEC
GPV      1425  PRYDLSELGTILAQSLAMTI.IDIITKDNKDHLTE........FKVLANDDDINSLITEF
FPaV     1416  ARWDIVQLEEGLAQSLAITI.IEIITKETKDHLTE........FKVLSNDDDINSLITEF
HMPV     1403  NYFHGNNLIESLSAALACHW.CGILTEQCI.............ENNIFKKDWGDGFISDH
AMPV     1403  NFFHGNNLVESLSAALACHW.CGILTEQCN.............ENNIFRREWGDGFVTDH
RSV      1484  DYFHNTY...ILSTNLAGHW.ILIIQLMKD.............SKGIFEKDWGEGYITDH
MPV      1420  DYFNGENMIETLSTCLAGHW.IIILMLMKE.............SQGIFDKEWYDGFVTDH
EBOV     1409  IEDDLIRLPHLSGWELAKTIMQSIISDSNNS.S...........TDPISSGETRSFTTHF
VSV      1351  KNLAPAEQSYQVGRCIGFLY.GDLA.YRKSTHAEDSSLFPLSIQGRIRGRGFLKGLLDGL
RABV     1369  ESLSGREKSHHIGSAQGLLY.SILV.AIHDSGYNDGTIFPVNIYGKVSPRDYLRGLARGV
```

```
NiV(B)   1535  LIVDPELFALYLGQSIAI.KWAFEIHHRRPRGRHTMVDLLSDLISNTSKHTYKVLSNALS
NiV(M)   1535  LIVDPELFALYLGQSISI.KWAFEIHHRRPRGRHTMVDLLSDLVSNTSKHTYKVLSNALS
HeV      1535  LMVDPELFALYLGQSISV.KWAFEIHHRRPHGRHTMVDLLSDLISNTSKHTYKVLSNALS
MojV     1528  LLVDVNLLSVYLGQACSN.KWAFDIHFKRPQGKWQMIELLTDLLSDTSVHVFRVLTNALS
LayV     1528  LLVDVNLLSIYLGQACSN.KWAFDIHFKRPQGKWQMIELLTDLLSETSVHVFRVLTNALS
GhV      1543  LLVDPELFSYYLGMYSAL.KWSFDIHYRRPRGKNAMVEIIRDLLNQASRHSYKVIANALS
PIV3     1478  LTLDILVFLKTFGGLLVN.QFAYTLYSLKIEGRDLIWDYIMRTLRDTSHSILKVLSNALS
SeV      1478  MVIDVPLFCSTFGGILVN.QFAYSLYGLNIRGREEIWGHVVRILKDTSHAVLKVLSNALS
GSqRV    1478  MIVDIPLFCSTFGGVLVN.QMAYSLYGLNIRGREEIWDYIVRVLKDTSHAVLKVLSNALS
MeV      1476  LLIEPRLFTIYLGQCAAI.NWAFDVHYHRPSGKYQMGELLSSFLSRMSKGVFKVLVNALS
RDV      1476  LLVEPRLFTVYLGQCTAI.NWAFDIHYHRPSGKYQMGELLTSFLARMSKSVFKVFVNALS
CDV      1476  LLVEPRLFTVYLGQCAAI.NWGFEIHYHRPSGKYQMGELLFSFLSRMSKGVFKILANALS
BPV      1482  LLVNPTEFSLFLGMYMAI.NWSFDIYYRRPEGKYQMVEFLQTIMTTAPKSSFRALSNALS
BeV      1459  LLVEPDEFVLYLGMYTAI.NWAFDIYYRRPEGKYQMTEYLQAILITASHSITKVISNALS
PMPV1    1473  MLVDPSEFALYLGLGVAV.NWSFDIYYRRPQGKYQMVEYLASNMRIMSRSILSVLANALS
MMLPV1   1460  MLVDPSQLTIHIGLSIAI.NWAYNIYYRRPEGKYQMVEYLYTIMIQSPRGYFSVLANALS
RuV      1497  LLVDPSLLTLYLGLSISI.KWAFNIYYKRPEGKYQMVEKLHAILANTSKSYFTVIMNAFS
BaVV     1485  MIVDPKLLCLHLGQAIAV.NWSFEIHYRKPRGPYEMVDLLHKLLGRSSHGSLSVLVNALS
ASPV     1481  LLIDINNFCIALGGLLVN.QFAYEIYTLGVEGRHLIYDHVFNRLRITSSAVFKVLTNAVS
FLDV     1471  MLSNPDDVLSSLGCMLVL.RFAYECYARHIEGKEQYISYVSDKIETMSRSNLSILINSLT
APV      1456  QNCDVVKLFEYAALEILL.DCAYQLYYLRVRGLDNMVLYMSSLLKNMPGILLSNIAATIS
PPMV1    1456  QNSDVVRLFEYAALEVLL.DCSYQLYYLRVRGLNNIVLYMSDLYKNMPGILLSNIAATIS
NDV      1456  QNSDVVRLFEYAALEVLL.DCAYQLYYLRVRGLNNIVLYMNDLYKNMPGILLSNIAATIS
APV17    1456  QNCDVVRLFEVAALEILL.DCAFQMYYLRVKGYSNIVMYMADLFRNMPGILLSNIAATIS
HPIV2    1484  SYTKIDLVFKLMAWNFLL.ELAFQMYYLRISSWTNIFDYTYMTLRRIPGTALNNIAATIS
MuV      1486  CYTYIDSVFVYSGWALLL.ELSYQMYYLRIQGIQGILDYVYMTLRRIPGMAITGISSTIS
PIV5     1480  MYTKLDELFMYCGWELLL.ELSYQMYYLRVVGWSNIVDYSYMILRRIPGAALNNLASTLS
WTSPV    1438  STADARSVLFSLATTLLL.DSASDLYFSRIRGVAEMVSWWASRLAISPHKIWLPLKSVFN
WHPV     1435  AMTKVKWIFDGMAIECIE.SLLVESLVSGCKTTSDVINFLRCRLRSIPFKSYSKLSSVLT
GPV      1476  ILVDPSEFMLYLGLSITI.NWAFDIYYRRPQGKYQIIEYLSTYLRISSKSFLNVLSNALS
FPaV     1467  IMVNPEQFSLYLGLAMSI.NWAYDIYYRRPEGKYQMVEYMNTIMNQTSKSYFMVLSNAFS
HMPV     1449  AFMDFKIFLCVFKTKLLC...SWGSQGKNIKD.EDIVDESIDKLLRIDNTFWRMFSKVMF
AMPV     1449  AFIDFKTFVGVFKTKLLC...GWGSRGGDIKD.RDMIDESIDKLIRVDNSFWRMFSKVIL
RSV      1527  MFINLKVFFNAYKTYLLCFHKGYGRAKLECD...MNTSDLLCVLELIDSSYWKSMSKVFL
MPV      1466  MFLDLQLFLSSFKTFLTVFNFAYLKVGSNIE...EITGNQANLLELLDLGYWKNMYKVFS
EBOV     1457  LTYPKIGLLYSFGAFVSY.YLGNTILRTKKLTLDNFLYYLTTQIHNLPHRSLRILKPTFK
VSV      1409  MRASCCQVI........H.RRSLAHLKRPANAVYGGLIYLIDKLSVSPPFL.....SLTR
RABV     1427  LIGSSICFL........T.RMTNININRPLELVSGVISYILLRLDNHPSLY.....IMLR
```

```
NiV(B)  1594 HPRVFKRFVNCGLLLPTQGP....YLHQQDFEKLSQNLLVTSYMIYLMNWCD..FKKYPF
NiV(M)  1594 HPRVFKRFVNCGLLLPTQGP....YLHQQDFEKLSQNLLVTSYMIYLMNWCD..FKKSPF
HeV     1594 HPRVFKRFVNCGLLLPTQGP....YLHQQDFEKLSQNLLITSYMNYLMNWCD..FKKFPF
MojV    1587 HPRVFKKFWDSGLLSPHYGP....YFYNQDTTKLAIDLIISSYTMYLTIWLE..GRTVSF
LayV    1587 HPRVFKKFWDSGLLSPYYGP....YFYNQDVTKLAIDLIISSYTMYLTVWLE..GRTVNF
GhV     1602 HPRVLKKFINSGLVTPSYGP....YVYQQDFYVMSQNLLISSYNMFLTKILN..SEIVRI
PIV3    1537 HPKVFKRFWDCGVLNPIYGP....NTASQDQIKLALSICEYSLDLFMREWLN..GVSLEI
SeV     1537 HPKIFKRFWNAGVVEPVYGP....NLSNQDKILLALSVCEYSVDLFMHDWQG..GVPLEI
GSqRV   1537 HPKIFKRFWNSGVVEPVYGP....NLSSQDKTLLALSVCEYSVDLFMHDWQG..GLPLEV
MeV     1535 HPKIYKKFWHCGIIEPIHGP....SLDAQNLHTTVCNMVYTCYMTYLDLLLNEELEEFTF
RDV     1535 HPKIYRKFWHSGIIEPIHGP....SLDTQNLHITVCNMIYSCYMTYLDLLLNDELDEFTY
CDV     1535 HPKVYRRFWDSGMIEPVHGP....SLDSQNLHITVCNLIYNCYMIYLDLLLNDELDDFSF
BPV     1541 HPTILQRFWDSGLIEPSYGP....NLLHQDFVRISIDLMITSYVTYIDYWLE..DNVLEY
BeV     1518 HPRVFQRFWDSGLIEPIYGP....NVQNQDYNRITQELICGAYQLYLNFWLS..DEEVEY
PMPV1   1532 HPKVFRRFWDVGLVEPIYGP....NVNTQNYSQIAIDLITKSYEIYIDYWLN..GKALEY
MMLPV1  1519 HPQIFNKFWDAGYVEPIYGP....NISSQDFIRIAIDYMILCYSTYLNYWTN..DNSIQY
RuV     1556 HIRVLNRFWDNGLIEPIYGP....NLLNQDYFSIVVDYLIKSYITYLDYWLD..DNNPEF
BaVV    1544 HLRIFKKFWDSNLIEPITGH....LLDQQNLITISIEFLTECYRSYLHYWGGHKSNNLQY
ASPV    1540 HPRIFKRFWDFGVVKPNRGA....NLKAQDFKLHALDICMHAVEYFMNQWDC..GTPLEV
FLDV    1530 HPRVFRKYCDEGVLVPDRGQ....TLSTQDFRAHSVEYCIASLQRFFEKWTI..SGNITI
APV     1515 HPVIHSRLHSVGLINHDGAH....QLASVDFVETSARLLTTCIRRIVTGLHS..GNKYDL
PPMV1   1515 HPIIHSRLNAVGLVGHDGSH....QLADTDFIEMSAKLLVSCTRRVVSGLYA..GNKYDL
NDV     1515 HPIIHSRLNAVGLINHDGSH....QLADIDFVEVSAKLLVSCTRRVVSGLYA..GNKYDL
APV17   1515 HPVIHNRLCNAGILSHGSAH....QLANVDFIELSANLLSACTSRVLTSLIN..GEQLSL
HPIV2   1543 HPKLLRRAMNLDIITPIHAP....YLASLDYVKLSIDAIQWGVKQVLADLSN..GIDLEI
MuV     1545 HPRILRRCINLDVIAPINSP....HIASLDYTKLSIDAVMWGTKQVLTNISQ..GIDYEI
PIV5    1539 HPKLFRRAINLDIVAPLNAP....HFASLDYIKMSVDAILWGCKRVINVLSN..GGDLEL
WTSPV   1497 SRKVLTRWLEDGIFIPAGSP....NFYNSISIETIHRWIINAIEHMLKN....PDVLLNH
WHPV    1494 SKKVRKQE..ERIQPRRAGP....NSRSM...........WTYPEIQTRILCAVDELLDN
GPV     1535 HPRVFRRFWDMGFVEPIYGP....NLNTQNFTQIAIDLLTRSCEIYLDYWLE..GGDLEY
FPaV    1526 HPKVFAKFWDQGLVEPIYGP....NLHTQDYTRIAIDFLVLSYQNYLDYWLS..DNEVPY
HMPV    1505 ESKVKKRIMLYDVKFLSLVG....Y...............IG....FKNWFIEQLRSAEL
AMPV    1505 EPKVRKRVMLFDVKILSLVG....Y...............AG....FKNWFIDHLRSSDL
RSV     1584 EQKVIKYILSQDASLHRVKG....C...............HS....FKLWFLKRLNVAEF
MPV     1523 ETKVRLALLKQDLSFNSVKN....S...............SS....FRHWFINSLQEVQC
EBOV    1516 HASVMSRLMSIDPHFSIYIGGAAGDRGLSDAARLFLRTSISSFLAFIKEWIINRGTIVPL
VSV     1455 SGPIRDELETIPHKIPTSYP........................TSN.............
RABV    1473 EPSLRGEIFSIPQKIPAAYP........................TTM.............
```

```
NiV(B)  1648 ....LIAEQDETVINLREDIITSKH......LCVIIDLYANHHKPPWIIDLNPQ.EKICV
NiV(M)  1648 ....LIAEQDETVISLREDIITSKH......LCVIIDLYANHHKPPWIIDLNPQ.EKICV
HeV     1648 ....LIAEQDEAVVELREDIITSKH......LCMIIDLYANHHKPPWIIDLNPQ.EKICV
MojV    1641 ....LMAEQSVDAVDIRSQTVQAKH......LCMLCDLYCNEQGPPHIRDLMPH.QKIDI
LayV    1641 ....LMAEQSVDAIEIRTQTIQAKH......LCMLCDLYCNEQGPPHIRDLLPH.QKIDI
GhV     1656 ....MIAEQDENVVEIREKTQLSKY......LCLLIDLYAYRGEIPWIVDLDPF.EKAIV
PIV3    1591 ....YICDSDMEVANDRKQAFISRH......LSFVCCLAEIASFGPNLLNLTYL.ERLDL
SeV     1591 ....FICDNDPDVADMRRSSFLARH......LAYLCSLAEISRDGPRLESMNSL.ERLES
GSqRV   1591 ....FICDNDPDVADMRKSSFLARH......LAYLCSLAEISKDGPRLESMTSL.ERLNA
MeV     1591 ....LLCESDEDVVPDRFDNIQAKH......LCVLADLYCQPGTCPPIRGLRPV.EKCAV
RDV     1591 ....LLCESDEDIVADRFDNIQARH......LCVLSDLYCNPRGCPTIRGLQPV.EKCAI
CDV     1591 ....ILCESDEDVIPERFDNIQARH......LCILSDLYCNPRDCPQIRGLTPT.QKCAV
BPV     1595 ....MLTESDENVIDQRYEITQARH......LCFLSCLYLQRNDMVMIRGMTSI.EKCAK
BeV     1572 ....LITEGEEELVDQRFELVQSRH......LCVLSSLYIRRQDMPQIRGLTSI.EKCTV
PMPV1   1586 ....IITETNQDIVDQRYEGIQSRH......LCFVSCLYVERKYMPPILGLTSL.EKCSV
MMLPV1  1573 ....LLTEYSDDIIEQRFEIVQSKH......LCMLNNLYNQRSDMPIIRGLTSI.EKCTI
RuV     1610 ....LITESLFRSIDQRYESVQSRH......LCTLTSLYLTRSNMPRIKGLTSI.QKCQL
BaVV    1600 ....IICEPDEDVITTRYDLVQAKH......LAMLNDYYNFSVAVPHIRGLEPL.DKCKV
ASPV    1594 ....YLCESDLDVVDERRETFTARH......LAFLCSFYGLSSRGPKLQGLDSL.QRYSA
FLDV    1584 ....LLCDQDEVIIGERELTLKGRI......KAMIICIYCLTENSPSLVGLNTK.ERDEV
APV     1569 ....LFPSVLDDNLTEKMLQLISRL......CCVLVLLYSTTKAIPKIRGLSAE.EKCAT
PPMV1   1569 ....LFPSVLDDNLSEKMFQLISRL......CCLYTVLFATTREIPKIRGLSAE.EKCSV
NDV     1569 ....LFPSVLDDNLNEKMLQLISRL......CCLYTVLFATTREIPKIRGLSAE.EKCSI
APV17   1569 ....AFPSVLEDNLTDKMFLLIARY......TSLLTLLFSARVPIPNIKGLSAE.DKCRA
HPIV2   1597 ....LILSEDSMEISDRAMNLIARK......LTLLALVKGENYTFPKIKGMPPE.EKCLV
MuV     1599 ....VVPSESQLTLSDRVLNLVARK......LSLLAIIWANYNYPPKVKGMSPE.DKCQA
PIV5    1593 ....VVTSEDSLILSDRSMNLIARK......LTLLSLIHHNGLELPKIKGFSPD.EKCFA
WTSPV   1549 ....LVVADSKLEVQELKKDLVMRE......IWMIGALLGKTDRLNNFRDMEDK.TK..S
WHPV    1537 ....PEMLDEMPIITDRLEGIKPRRTRSTTIRCLLSLMENGNLNTREIRTNVKN.D.NEL
GPV     1589 ....MLPESIQDIVDQRFENIQSRH......LCILCCLYLEREFMPIILGLTAI.EKCAV
FPaV    1580 ....VITESDEEIIDQRFEVTQARH......LCMLNNLYNTRDNMPKIRGLTSI.NKCFI
HMPV    1542 HEVPWIVNAEGDLVEIKSI............KIYL...QLIEQSLFL.............
AMPV    1542 CEVPWVVNADSEIVEVSAV............KIYL...QLLRVSSPL.............
RSV     1621 TVCPWVVNIDYHPTHMKAI............LTYI...DLVRMGLINIDK..........
MPV     1560 TSVPWVVNVTRNPTHLKGV............LQYM...KMIESGMIQGYS..........
EBOV    1576 ....WIVYPLEGQNPTPVNNFLHQI......VELL...VHDSSRQQ.............A
VSV     1478 .................RDM.......GVIVRNY....FKYQCRLIEKGKYRSHYSQLWL
RABV    1496 .................KEG.......NRSILCYLQHVLRYEREIITA...SPENDWLWI
```

```
NiV(B)  1697 LRDFISKSRHMDTSS......RSWNTS...DLDFVIFYASLTYLR.............RG
NiV(M)  1697 LRDFISKSRHVDTSS......RSWNTS...DLDFVIFYASLTYLR.............RG
HeV     1697 LRDFISKCRHTDVSS......RSWNIT...DLDFMVFYASLTYLR.............RG
MojV    1690 LSDFIKKTRLNIVGS......DSWNIH...NLHINVYPASLTYLR.............RG
LayV    1690 LSDFIRKTRLNIVGS......DAWNVH...NLHINVYPASLTYLR.............RG
GhV     1705 LENYLFEQCQKEGMI......GVWNIT...SPKFSQYRASVTYLR.............RS
PIV3    1640 LKQYLELNIKEDPTL......KYVQIS...GLLIKSFPSTVTYVR.............KT
SeV     1640 LKSYLELTFLDDPVL......RYSQLT...GLVIKVFPSTLTYIR.............KS
GSqRV   1640 LKDYLEMTFLDDPVL......RYSQLT...GLVLKIFPSTLTYIR.............KS
MeV     1640 LTDHIKAEARLSPAG......SSWNIN...PIIVDHYSCSLTYLR.............RG
RDV     1640 MTKHIEAEAKLSPAG......PSWNIG...PIVIDHFSCSLTYLR.............RG
CDV     1640 LSGYLKSKALESHVG......LTWNDK...PILIDQYSCSLTYLR.............RG
BPV     1644 LTDNLKEKKVTSGFL......QDWNLE...VLPITVHAASLTYIR.............RG
BeV     1621 LTDRLEKMRFSQGVS......ADWHID...PLPVTVYPASMTYIR.............RG
PMPV1   1635 LQDALDTMIKVQPNS......KSWNTK...LLKVEIYPVSSTYLR.............RG
MMLPV1  1622 LTDALRNAQFGNPGS......VSWNLD...PLEVVAYPASLTYLR.............RG
RuV     1659 LFDNLISERRAKGTT......LKWNLD...PLNVIIYPASLTYLR.............RG
BaVV    1649 LDESLKKAAELNPNS......RDWNLN...NLNIIAYPTSLTYIR.............RG
ASPV    1643 IRDYLEVQVTKDHTS......RFWQLT...GLIIRAVPSTVSYLM.............QS
FLDV    1633 LTEYLEEVGQSNIES......RYWVLG...SIGIKKYNTSLTYIR.............RS
APV     1618 LTSYLLSDAVRPTMS....SAQIDSLL...SPNIVTFPANLYYMS.............RK
PPMV1   1618 LTEYLLSDAVKPLLK....SEQVSSIM...SPNIITFPANLYYMS.............RK
NDV     1618 LTEYLLSDAVKPLLR....PEQVSSIM...SPNIITFPANLYYMS.............RK
APV17   1618 LTQHLLNMPGESRLS....PRQELIVL...QPQLVTFPTNLYYIS.............RK
HPIV2   1646 LTEYLAMCYQNTHHLDPDLQKYLYNLT...NPKLTAFPSNNFYLT.............RK
MuV     1648 LTTHLLQTVEYVEHIQIEKTNIRRMII...EPKLTAYPSNLFYLS.............RK
PIV5    1642 LTEFLRKVVNSGLSSIENLSNFMYNVE...NPRLAAFASNNYYLT.............RK
WTSPV   1596 LYDQFEICAP...........FTWRETFTSKYSGVTLTGTYFYHA.............RV
WHPV    1591 FLRFIQQSI...PPR......FIKEL.....SPKGIVTGNLTYVM.............RQ
GPV     1638 LHNALKNQKYMNTRY......FSWNLE...PLRVCVYPVSMTYIR.............RG
FPaV    1629 LSESLKHGKLLNISS......YTWNLE...LLEVIIYPASFTYIR.............RG
HMPV    1574 .........................................RITV......LNYTDMAHA
AMPV    1574 .........................................RITV......LNYSDMAHA
RSV     1656 ..IYIKNKH.....................KFNDEFYTSNLFYIN......YNFSDNTHL
MPV     1595 ..A.................................NISSVLSIP......YNYPDMAHM
EBOV    1610 FKTTISDHVH..PHD.....NLVYTCK...STASNFFHASLAYWRSR.HRNSNRKYLARD
VSV     1510 FSDVLSIDFI......................GPFSISTTLLQILYKPFLSGKDKNELRE
RABV    1529 FSDFRSAKMT....................YLSLITYQSHLLLQRVERNLSKSMRDNLRQ
```

```
NiV(B)  1735 IIKQLRIRQVTE.V.VDTTTMLRD.........NII...V..ENP...............
NiV(M)  1735 IIKQLRIRQVTE.V.IDTTTMLRD.........NII...V..ENP...............
HeV     1735 IIKQLRIRQVTE.V.IDTTTMLRD.........NIL...V..ENP...............
MojV    1728 VIKQLRIRQDIGEM.FDIIEMDRK.........YH....K..FMV...............
LayV    1728 VIKQLRIRQDIGEM.FDIIEMDRK.........YH....K..YMV...............
GhV     1743 SVKQLRIRQNDE.V.IEISRLEND.........VMV...K..FNS...............
PIV3    1678 AIKYLRIRGISPPEVIDDWDPIED.........EN.......MLD...............
SeV     1678 SIKVLRTRGIGVPEVLEDWDPEAD.........NA.......LLD...............
GSqRV   1678 AIKVLRTRGIGVPEVLEDWDPDTD.........NS.......LLD...............
MeV     1678 SIKQIRLRVDPGFI.FDALAEVNV.........SQPKVGS..NN................
RDV     1678 SIKQIRLRVDPGFI.FEALTVTDP.........QKPNFQH..EQ................
CDV     1678 SIKQIRLRVDPGFI.TDAVGCLER.........RPLRNNS..TS................
BPV     1682 TVKHIKLRNYLSAE.AFGYDKAIS.........QSQDKKI..FD................
BeV     1659 TIKHIRLRNYLSGE.SEIMESIKI.........QNQEIRV..FD................
PMPV1   1673 SIKHIRLRKFLVFE.DPLSDKPKL.........DPLDPKS..FI................
MMLPV1  1660 TIKHIRLRNVLSKS.ILYINEVKF.........EVP..NI..TQD...............
RuV     1697 AIKHIRIRKSITDP.ASSTRDTKW.........DPL...I..HPD...............
BaVV    1687 TIKQLRLRSPDPSL.LIGSSPVSN.........YPS.KQ... .................
ASPV    1681 AVKQFRLRGLGVPTVLETWNPTFN.........DL.......LCD...............
FLDV    1671 LVKQYRLRDLDVQLLIDEMAEQDM.........ESL...S..MAD...............
APV     1658 SLNLIREREDRDTVLALIFPP.DV.........IM.......EAP...............
PPMV1   1658 SLNLIREREDRDAILALLFPQ.EP.........LL.......ELR...............
NDV     1658 SLNLIREREDRDTILSLLFPQ.EP.........LL.......ELR...............
APV17   1658 SLNIVREREDREGILELIFPA.YD.........DI.......TES...............
HPIV2   1690 ILNQIRESDEGQYIITSYYES.FE.........QL.......ETD...............
MuV     1692 LLNAIRDSEEGQFLIASYYNS.FG.........YL.......EPI...............
PIV5    1686 LLNSIRDTESGQVAVTSYYES.LE.........YI.......DSL...............
WTSPV   1632 MLTELRSRNVVHHTPTLNQECINP.........AI.......IC................
WHPV    1624 CLIILRDRNFVIGNHK.......K.........TIIQTKV..TCT...............
GPV     1676 AVKHIRLRRFIQFN.DPLSDKLKI.........DPLNMRS..FV................
FPaV    1667 IIKHIRLRGSLSSE.VIAYNKLQS.........NQKEPKI..HLP...............
HMPV    1587 LTRLIRKKLM.CDN...A.LLTPI.PSP................................
AMPV    1587 ITRLIRRKSM.HDN...V.PSISR.TLS................................
RSV     1687 LTKHIRIANSELEN...NYNKLYH.PTPETLE.NILTNPV..KCNDKKTLNDYCIGKNVD
MPV     1614 MTKIIRNRGH.MSY...DYPKMKK.SL.................................
EBOV    1659 SSTGSSTNNSDGHI.ERSQEQTTRDPHDGTERNLVLQMSHEIKRT...............
VSV     1548 LANLSSLLRSGEGW.....EDIHVK....FFTKDI....L..L.................
RABV    1569 LSSLMRQVLGGHGE.....DTLESDDNIQRLLKDSLRRTR..W.................
```

```
                    .
NiV(B)  1764  PIKTGVLDI......R...........G.....C..........................
NiV(M)  1764  PIKTGVLDI......R...........G.....C..........................
HeV     1764  PIKTGVLDI......R...........G.....C..........................
MojV    1757  ETAINYKPR......S...........S.....C..........................
LayV    1757  ETAINYKPR......S...........T.....C..........................
GhV     1772  SILSQVDLN......D...........S.....C..........................
PIV3    1707  NIVKSINDN......CNK....DNK..GNKINNFWGLAL................KNYQV
SeV     1707  GIAAEIQQN......IPL....G....HQTRAPFWGLRV................SKSQV
GSqRV   1707  NIASEVQQN......IPI....V....QGTRAPFWGLRV................NKSQV
MeV     1710  ........I......S...........NMSIKDF..........................
RDV     1710  ........A......A...........DMHISNF..........................
CDV     1710  ........K......A...........SELTSGF..........................
BPV     1714  .MPLNNR.K......F...........NDTLSYY..........................
BeV     1691  .MPTGVV.L......T...........GEHESYY..........................
PMPV1   1705  .IMKNTT.K......G...........INTTFKA..........................
MMLPV1  1691  KLFEEQL.L......G...........YNKGAYY..........................
RuV     1727  KRFLHILET......S...........KISGVKV..........................
BaVV    1715  .MQTHAL.T......A...........PSIESLE..........................
ASPV    1710  TVEAEKDKN......MKG......T..SEYPRDYWGIEI................PRSNI
FLDV    1702  PIFDQVNKT......ET..........MG.SVSLWQS.......................
APV     1686  DIR....SL......GSR....TDDPMTKSPAAFIK.....................ELA
PPMV1   1686  PVQ....DI......GAR....VRDPFTQQPASFIQ.....................ELD
NDV     1686  PVR....DI......GAR....VKDPFTRQPASFIQ.....................ELD
APV17   1686  TKA....QW......DVA....ADDPFLKGSSLVNH.....................EIR
HPIV2   1718  IILHSTLTA......PYD....NSENSNK..VRFIP.....................FDI
MuV     1720  LMESKIFNL......SSS....ESASLTE..FDFI......................LNL
PIV5    1714  KLTPHVPGT......SCI....EDDSLCT..NDYI......................IWI
WTSPV   1660  ........N......DAT....IDR........KIQ........................
WHPV    1651  AEDQDIR.T......G............................................
GPV     1708  .IDKNIT.K......G...........INTSFRA..........................
FPaV    1700  YKYLTVK.N......K...........CDKLSLS..........................
HMPV    1609  ...............MVNLTQVIDPTEQLAYFPKITFER.....................
AMPV    1609  ...............PAELAPVVEPTVQMNLFPKITFER.....................
RSV     1740  SIMLPLLSNKKLIKSPTMIRTNYSKQDLYNLFPTVVIDK.....................
MPV     1636  ...............TFSM.TDMSDSYMLNLFPKVECSY.....................
EBOV    1703  TIPQESTHQ......GPSFQSFLSDSACGTANPKLNFDRSRHNVKSQDHNSASKREGHQI
VSV     1576  .CPEEIRHACK.....FGIAKDNN...KDMSYPPWGRES..................RGT
RABV    1605  .VDQEVRHAARTMTGDYSPNKKVS...RKVGCSEWVCSA..................QQV
```

```
                     .           .         .                     .
NiV(B)  1776  I...IYNLEEILSMNT..KSSSKKIFNLNSKPS...V.........ENHKYRRIGL.NSS
NiV(M)  1776  I...IYNLEEILSMNT..KSASKKIFNLNSRPS...V.........ENHKYRRIGL.NSS
HeV     1776  I...IYNLEEILSMNT..KSTSRKVFNLGSKLS...V.........ENHKYRRIGL.NSS
MojV    1769  T...VLNLNDIDRPLN..STLYIDLEDSVPVNG...W.........ESHKYRRVGI.NST
LayV    1769  T...VLNLSDIDRPLN..STLYIDLEDSVPVNG...W.........ESHKYRRVGI.NST
GhV     1784  F...LMNLEEAIFQST..EIMTSHSVVSRSPRL...L.........ENHMNRRIGI.NST
PIV3    1739  LK.IRSITSDSD..NNDGLDVSTGGLTLPQGGN...Y.........LSHQLRLFGI.NST
SeV     1737  LR.LRGYKEITR....GEIGRSGVGLTLPFDGR...Y.........LSHQLRLFGI.NST
GSqRV   1737  LR.LRRYREMTN....NEIGRSGVGLTLPFDGR...Y.........SSHQLRLFGI.NST
MeV     1719  RP.PHDDVAKLLKDIN..TSKHNLPISGGSLAN...Y.........EIHAFRRIGL.NSS
RDV     1719  RP.PYDGVAELLGTIN..SSKHNLPILGTGVYN...Y.........EVHAFRRIGL.NSS
CDV     1719  DP.PKDDLAKLLSQLS..TRTHNLPITGLGVRN...Y.........EVHSFRRIGI.NST
BPV     1729  FP.ASSLSTDQF......QEFNFRIDTSNDKNR...W.........ESHVFRRIGA.NST
BeV     1706  FP.ATLLFSLDF......QQSQDNIFDKNITNK...W.........ENHVTRRVGI.NST
PMPV1   1720  FQ.ASILLSDDF......RKLNQLEACIPTKNR...W.........ESHTLRRVGI.NST
MMLPV1  1707  FP.ITYLFTDDF......RQTLGNHKIAPVKNK...W.........ECHVLRRLGL.NST
RuV     1744  IP.YNLLFSTDL......NKHLNIMEDTISSNS...W.........EIHVTRRVGL.NST
BaVV    1730  LP.ININLNDLFKTVGEERSVPGNELLSTNANE...Y.........SHHIFRRTGL.NST
ASPV    1740  FP.CHGLVPSLT..R..DEEKYDTSIELPLGPS...Y.........LSHQLRIVGV.NST
FLDV    1722  IK.VKDLTLDR......NPRAESAEYRIPTTCE...I.........PSQILRIVGV.NST
APV     1711  IN.APARYDTCFPSQL..TQ..MEGTQSRPDEM...L.........IRYLFRGIGT.ASS
PPMV1   1711  LN.APARYDAFTLSEV..RP..EHTLPNSEEDY...L.........VRYLFRGIGT.ASS
NDV     1711  LS.APARYDAFTLSKV..CF..EHTLLNPKEDY...L.........VRYLFRGIGT.ASS
APV17   1711  LD.GPARYGLLFADCK..QEGSRGSLIVADQVP...M.........TRYLFRGIGL.ASS
HPIV2   1745  FP.HPESLEKYPLP.V..DHDSQSAISTLIPGP...P.........SHHVLRPLGV.SST
MuV     1746  EL.SEASLEKYSLP.S..LLMTAENMDNPFPQP...P.........LHHVLRPLGL.SST
PIV5    1740  IE.SNANLEKYPIP.N..SPEDDSNFHNFKLNA...P.........SHHTLRPLGL.SST
WTSPV   1670  EQ.IRHTYV.........KWKIMPVIASTTECD...Y.........RHYILRNVGM.NTT
WHPV    1660  ......NLS.......GKEITGDLVWKYEDKKK...I.........GCYLLRQSGV.NST
GPV     1723  FQ.SVLLLSDDF......RKLNHLEHYVPTKNR...W.........ESHIQRRIGV.NST
FPaV    1716  LT.IENLTSDDL......RIFNTKRSYKENKNR...W.........ESHILRRIGI.NST
HMPV    1633  ....LKNYDTSSNYAK..G...........KL.....TRNYMILLPWQHVNRYNFVFSST
AMPV    1633  ....LKNYETVSGSTR..G...........KL.....TRNYMVMLPWQHINRFNFVFSST
RSV     1779  ....IIDHSGNTAKSN..QLYTTTSHQIPLVHN....STSLYCMLPWHHINRFNFVFSST
MPV     1659  ....MSGYLDKLDDTL..QLLK.....KP.PVG....RKVPSVALPWHHCNRYNFVFSST
EBOV    1757  IS.HRLVLPFFTLSQG..TRQLTSSNESQTQDEISKYLRQLRSVIDTTVYCRFTGI.VSS
VSV     1609  ITTIPVYYTTTP.YPK..MLEMPPRIQNP.................LLSGIRLGQL.PTG
RABV    1643  AVSTSANPAPVS.ELD..IRALSKRFQNP.................LISGLRVVQW.ATG
```

```
                      .         .          .         .         .          .
NiV(B)   1818  SCYKALNLSPLIQRY.LPSGAQRLFIGEGSGSMMLLYQSTLGQSIS.FYNSGIDGDYIPG
NiV(M)   1818  SCYKALNLSPLIQRY.LPSGAQRLFIGEGSGSMMLLYQSTLGQSIS.FYNSGIDGDYIPG
HeV      1818  SCYKALNLSPLIQRY.LPAGSQRLFVGEGSGSMMLLYQQTLGCSIS.FYNSGIDGDYIPG
MojV     1811  SGYKALELYSIIERY.YTPNSPRLFVGEGSGAMMSVYFKLLGKAIN.YYNSGISELEVVG
LayV     1811  SGYKALELYSIIERF.YTPGSARLFVGEGSGAMMSVYFKLLGKSVN.YYNSGISELDVVG
GhV      1826  SCYKAVDLTPIVKRY.LPSEGRRLFLGEGSGAMMYVYQNTLGPSLC.YYNSGVTDQGLEG
PIV3     1783  SCLKALELSQILMKE.VNKDKDRLFLGEGAGAMLACYDATLGPAVN.YYNSGLNITDVIG
SeV      1779  SCLKALELTYLLSPL.VDKDKDRLYLGEGAGAMLSCYDATLGPCIN.YYNSGVYSCDVNG
GSqRV    1779  SCLKALELTYLLSPL.VNKDKDRLYLGEGAGAMLSCYDATLGPCMN.YYNSGVYSCDVSG
MeV      1763  ACYKAVEISTLIRRC.LEPGEDGLFLGEGSGSMLITYKEILKLNKC.FYNSGVSANSRSG
RDV      1763  ACYKAVEISTLIKSS.MDPEEDSLFLGEGSGSMLITYKEILKLKKC.FYNSGVSAESRSG
CDV      1763  ACYKAVEIASVIKNE.FTSEEHGLFLGEGSGAMLTVYKELLRLSRC.YYNSGVSVESRTG
BPV      1769  SCYKALELSTYLRPY.IVDGLPRLFLGEGSGSMMATYYMVFGRSKN.YYNSGVSSNDAMG
BeV      1746  SCYKALELATFLNGK.VAASEPRLFLGEGSGAMMVTYYYILGQSRS.YYNTGVFDSDIVG
PMPV1    1760  SCYKAMEIGMYIVNK.VDLGGDRLFLGEGSGAMLSTYYMLLGPALC.YYNTGVLNTNLIG
MMLPV1   1747  SCYKAVEIGLYLKDK.IDLKGDRLFLGEGSGAMMTTYYAILGQATN.YYNTGVFASEVVG
RuV      1784  SCYKALEIGMYLLNF.LNKEGPRLFLGEGSGAMLTVYYFLLGPCLT.YYNSGVFNEIYVG
BaVV     1776  SCYKAVEICDFIKGH.VNHSGKRLFLGEGAGAMLSVYYYMLGPAYS.YFNTGVYSQSVRG
ASPV     1782  SCLKALELTSHLSQV.ANLTGNRLFVGEGAGAMLALYDSVLGPVTN.WYNSGISPRDLTG
FLDV     1762  SCSKAHELNSVICTL.PLEGKSCLFLAEGSGSMMTYYMLRHRLNNV.YFNTGVGSEDINS
APV      1753  SWYKAAHLLSIPEVR.LARHGNSLFLAEGSGAIMSLLELHVPHEVI.FYNTLFSNEQNPP
PPMV1    1753  SWYKVSHLLSIPEVR.CARHGNSLYLAEGSGAIMSILELHIPHETI.YYNTLFSNEMNPP
NDV      1753  SWYKASHLLSVPEVR.CARHGNSLYLAEGSGATMSLLELHIPHETI.YYNTLFSNEMNPP
APV17    1755  SWYKATNLLSLPEVR.QARFGGGLYLAEGSGAIMSLLELHIPHEKI.YYNTLFYNEYNPP
HPIV2    1788  AWYKGISYCRYLETQ.KIQTGDHLYLAEGSGASMSLLELLFPGDTV.YYNSLFSSGENPP
MuV      1789  SWYKTISVLNYISHM.KISDGAHLYLAEGSGASMSLIETFLPGEII.WYNSLFNSGENPP
PIV5     1783  AWYKGISCCRYLERL.KLPQGDHLYIAEGSGASMTIIEYLFPGRKI.YYNSLFSSGDNPP
WTSPV    1707  AQQKVIHILKKWSEA.LD.VESALCVAEGSGGILAAIGLIWPQAIL.FFNSLKMGVE.ND
WHPV     1694  SALKLW...NSLSDL.PIKNGKVLCLGEGSGSIAALIK.SLGARHI.YFNSLMLGN.FGS
GPV      1763  SCYKACEIGIYIMNK.IDLNGPRLFLGEGSGAMMTTYYFILGKCKC.YYNTGVLNTGLIG
FPaV     1756  SCYKAVEIADYIIEH.LDANGERLFLGEGSGAMMAVYYYMIGPTVC.YFNTGVCDTEAIG
HMPV     1671  GCKVSLKTC.IGKLMKDLNPKVLYFIGEGAGNWMARTACEY.PDIKFVYRSLKDDLD...
AMPV     1671  GCKISVKAC.IGRLIQDLNPTVFYFVGEGAGNWMARTACEY.PNAKFVYRSLKDDLD...
RSV      1829  GCKISIEYI.LKDLI.IKDPNCIAFIGEGAGNLLLRTVVELHPDIRYIYRSLKDCND...
MPV      1703  GCKVSVIDM.LPKHFRRSNLKVICFIGEGAGNLMLRAVLEVGGNIKLIYRSLKDPDD...
EBOV     1813  MHYKLDEVLWEIESF...KSAVTLAEGEGAGALLLIQ..KYQVKTL.FFNTLATESSIES
VSV      1648  AHYKIRSILHGMGIH....YRDFLSCGDGSGGMTAALLRENVHSRG.IFNSLLELSGSVM
RABV     1682  AHYKLKPILDDLNVF....PSLCLVVGDGSGGISRAVLNMFPDAKL.VFNSLLEVNDLMA
```

```
                   .               .         .         .         .
NiV(B)   1876  QRELKLFPSEY......SIAEEDPSLAGKLKGLVVPLFNGRPETTWIGNLDSYEYIINRT
NiV(M)   1876  QRELKLFPSEY......SIAEEDPSLTGKLKGLVVPLFNGRPETTWIGNLDSYEYIINRT
HeV      1876  QRELRLFPSEY......SIAEDDPSQSDKLKGLVVPLFNGRPETTWIGNLDSYEYIINRT
MojV     1869  QRELRLYPAEA......CIVDHNMGEEYGLLQSIIPLFNGRPETTWIGNMDCFVHIMRMI
LayV     1869  QRELRLYPAEA......CIVDHNLGEEYGLLKSIVPLFNGRPETTWIGNMDCFIHIMRMI
GhV      1884  QRELKMYPAEY......YLCSTSSNREEELLPSVIPLFNGRPETTWIGNIDAFRLITEFV
PIV3     1841  QRELKIFPSEV......SLVGKKLGNVTQILNRVKVLFNGNPNSTWIGNMECESLIWSEL
SeV      1837  QRELNIYPAEV......ALVGKKLNNVTSLGQRVKVLFNGNPGSTWIGNDECEALIWNEL
GSqRV    1837  QRELNIYPAEV......ALVGKKLNNVTSLNQRVKVLFNGNPGSTWIGNDECEALIWNEL
MeV      1821  QRELAPYPSEV......GLVEHR....MGVGNIVKVLFNGRPEVTWVGSIDCFNFIVSNI
RDV      1821  QRELAPYPSEV......SLVEHQ....LGAEKTVKVLFNGRPEVTWIGSVDCFNYIISNI
CDV      1821  QREISPYPSEV......SLVEHQ....LGLDKLVTVLFNGRPEVTWVGSVDCYKYILSQI
BPV      1827  QRALSIVPSEY......ALVAKNNPNDLQMMYDLQVLFNGKPESTWVGSMDSFKYIMNTV
BeV      1804  QRVLSLVPSEA......MMVAKNNPTELAFESDLITLFNGKPESTWIGSLASFTYIMEQI
PMPV1    1818  QRVFSIYPSEV......MLVAHNNTTDLDIESNIKVLFNGKPECSWVGDMECFSYIMNNC
MMLPV1   1805  QRVLTLSPAEA......YLVAKNNPNEMNLRKNIIPLFNGKPESTWIGNPSAFVYINNSI
RuV      1842  QRILDISPAEV......HLIIKNNPSNITLSKDLNVLFNGRPESTWVGNMECFQFIMNTI
BaVV     1834  QREFHPFPSEV......ALVSKQNCDNESLLSNVEVLFNGNPESTWLGNLDCFTYIINTV
ASPV     1840  QRELNIYPSEV......ALTDNNQKGVNDLMSRTKVLFNGRPEVTWIGSDTCERFITDSL
FLDV     1820  QRETDPSPSEP......VLVEGNLRIKHCLSEKVTVLFNGRPETTWVGNEDCESYVTTQI
APV      1811  QRHFGPTPTQFLNSVVYKNLQANVPCKDGFVQEYVPLWRENADESDLSSDKSVSYITSTV
PPMV1    1811  QRHFGPTPTQFLNSVVYRNLQAEVPCKDGFVQEFCPLWRENAEESDLTSDKAVGYITSVV
NDV      1811  QRHFGPTPTQFLNSVVYRNLQAEVPCKDGYVQEFCPLWRENAEESDLTSDKAVGYITSVV
APV17    1813  QRHFGPTPTQFLNSVVYRNLQAGIPCKDGYVQEFVTLWRDVAEESDLSSDKSVAFITSEI
HPIV2    1846  QRNYAPLPTQFVQSVPYKLWQADLADDSNLIKDFVPLWNGNGAVTDLSTKDAVAFIIHKV
MuV      1847  QRNFAPLPTQFIESVPYRLIQAGIAAGSGVVQSFYPLWNGNSDITDLSTKTSVEYIIHKV
PIV5     1841  QRNYAPMPTQFIESVPYKLWQAHTDQYPEIFEDFIPLWNGNAAMTDIGMTACVEFIINRV
WTSPV    1763  QRTDEYFPSEV........MSLNPEARESVKSRTVVLDGGTPLSTDLTQNSCIELIEKKV
WHPV     1747  QRTPEYFPSEY......LYIH........GEEDITIINSGRQDSTDLTRDECKDLILSTV
GPV      1821  QRVFQVYPSEV......MLVSHNNTMDITLEKDIRVLFNGKPECTWVGDMECFSYIMNSI
FPaV     1814  QRVLNLLPSEF......SLVAKNNMDNEVIMSNIKTLFNGKPESTWIGTLSSFTYIMNRI
HMPV     1726  ....HHYPLEYQRVIG.......ELS......RIIDSGEGLSMETTDATQKTHWDLIHRVS
AMPV     1726  ....HHFPLEFQRVLG.......NMN......RVIDGGEGLSMDTTDATQKTHWDLIHRIC
RSV      1884  ....HSLPIEFLRLYN.......G.H......INIDYGENLTIPATDATNNIHWSYLHIKF
MPV      1759  ....HHVPVEFLRLKP.......C.Y......PYIDTGGSLSLASTDATNKAHWDYLHLHW
EBOV     1867  EIVSGTTTPRMLLPVM..........SKFHNDQIEIILNNSASQITDITNPTWFKDQRARL
VSV      1703  .RGASPEPPSALETLG............GDK.SRCVNGETCWEYPSDLCDPRTWDYFLRLK
RABV     1737  .SGTHPLPPSAIMRGG............NDIVSRVIDLDSIWEKPSDLRNLATWKYFQSVQ
```

```
                .         .         .         .         .         .
NiV(B)   1930   A..GRSIGLVHSDMESGIDKNVEEILVEHSHLISIAINVM..MEDGLLVSKIAYT.PGFP
NiV(M)   1930   A..GRSIGLVHSDMESGIDKNVEEILVEHSHLISIAINVM..MEDGLLVSKIAYT.PGFP
HeV      1930   A..GRNIGLVHSDMESGIDKQVEEIMIEHSHLISIAINVM..IEDGVLVSKIAFA.PGFP
MojV     1923   K..SHSLGLVHSDMESGLDKESITILNEHSHLLSLAINLL..RFDGILVSKINPS.SSFP
LayV     1923   K..SHSLGLLHSDMESGVDKDNLIILNEHSHLLSLAVNLL..RFDGILVSKINPS.SSFP
GhV      1938   N..PLETVMTHSDMESGMTKDEETILVEHSHIISLHINLT..VEDSILVSKIAFG.NNLN
PIV3     1895   N..DKSIGLVHCDMEGAIGKSEETVLHEHYSVIRITYLIG..DDDVVLISKIIPT.ITPN
SeV      1891   Q..NSSIGLVHCDMEGGDHKDDQVVLHEHYSVIRIAYLVG..DRDVVLISKIAPR.LGTD
GSqRV    1891   Q..NSSIGLIHCDMEGGDQKDDKVVLHEHYSVIRIAYLVG..DRDALLVSKIAPR.LGTD
MeV      1871   P..TSSVGFIHSDIETLPNKDTIEKLEELAAILSMALLLG..KIGSILVIKLMPF.SGDF
RDV      1871   Q..TSSLGLIHSDIETLPTKDITEKLEELSAILALALLLG..KVGSVLVIKVMPI.SGDY
CDV      1871   S..ASSLGLIHSDIESLPDKDIIEKLEELSAILSMTLILG..KVGSVLVIKIMPV.SGDW
BPV      1881   G..LGSCTLVHSDMESSSEKDDFTIFEEQLHVISLACNLT..TEAGVLVTKIAPR.FDDN
BeV      1858   P..PHSVGFIHNDMESSFDKDPCLIMQEQVHSLALAINLG..NDYSYYICKLAPR.PNDY
PMPV1    1872   K..CDTFALVHNDMESSLEKNPEIILTEQVHSLCIALNML..KKDGIYICKVAPR.DDDY
MMLPV1   1859   K..SHSLALIHNDMESSFEKDTYTIACEQCHSLALALNLG..RSDSIYVTKLAPR.PNDY
RuV      1896   P..MHSLALIHSDIENTHNKDDEIVLLELAHILSMCINLG..ELGSSVIIKLAPK.KGDK
BaVV     1888   K..TNTCALIHCDLETIGDKTIKQGFEELCHVVCLALAVG..DLNSMFIMKLLPT.PEDW
ASPV     1894   C..DDRVSIVHSDMEGGSGKDEDAVMIEQYSVTRIAYRVG..SDDVTLVTKLAPR.LGSA
FLDV     1874   G..EDKVSLVHSDMESGIEKSNQAVLVEHVSLIKIFQGVP..TPGATLVSKIAPR.LGTN
APV      1871   P..FKSVSLMHCDIEVPMGSNQSYLDQVATNLALIAMHTI..RDKGVFIVKVLFS.MGYY
PPMV1    1871   P..YRSVSLLHCDIEIPPGSNQSLLDQLATNLSLIAMHSV..REGGVVIIKVLYV.MGYY
NDV      1871   P..YRSVSLLHCDIEIPPGSNQSLLDQLATNLSLIAMHSV..REGGVVIIKVLYA.MGYY
APV17    1873   P..RGSVSLLHCDIETTLDPSWAYLEQIATNLTLIGIHTL..REGGVFILKSLYS.HGFF
HPIV2    1906   G..AEKASLVHIDLESTANINQQTLSRSQIHSLIIATTVL..KRGGILIYKTSWL.PFSR
MuV      1907   G..ADTCALVHVDLEGVPGSMNSMLERAQVHALLITVTVL..KPGGLLILKASWE.PFNR
PIV5     1901   G..PRTCSLVHVDLESSASLNQQCLSKPIINAIITATTVL..CPHGVLILKYSWL.PFTR
WTSPV    1815   P..N.NLTLLTCDVEQFDGMQLVVLWR...NVLWLLVSRV..AANGRVLFKLSHT.KGTI
WHPV     1793   N..E..VSGITCDAESGDDTES..ILRTVRS...LISIVL..KEGGWTIIKVMMR.N.ES
GPV      1875   Q..THTLALVHNDMESSLEKTPETILQEQIHSLCLAVNLT..TPDGMYICKIAPR.YNDL
FPaV     1868   K..PHSLSMVHSDMESSHQKDPDTILKEQCYAMCLAMNLS..IRKSVYITKIAPK.DDDY
HMPV     1770   ...KDALLITLCDAEFKDRDDFFKMVILWRKHVLSCRICTTYGTDLYLFAKYHAK.DCNV
AMPV     1770   ...KDALLITLCDAEFKDRDDFFKMVTLWRKHVLSCRICTTYGTDLYLFAKYHAK.EQSI
RSV      1927   ...AEPISLFVCDAELPVTVNWSKIIIEWSKHVRKCKYCSSVN.KCTLIVKYHAQ.DDID
MPV      1802   ...TDPLNLIVCDAEISGVKHWLKILHRWYEHMTSCKHCLKSEHDKYLIIKYHAQ.DDLI
EBOV     1918   P...RQVEVITMDAETTENINRSKLYEAVYKLILHHIDPS..VLK.AVVLKVFLS.DTEG
VSV      1750   AGLGLQIDLIVMDMEVRDSSTSLKIETNVRNYVHR...IL..DEQGVLIYKTYGTYICES
RABV     1785   KQVNMSYDLIICDAEVTDIASINRITLLMSDFAL....SI..DGPLYLVFKTYGTMLVNP


                      .         .         .         .
NiV(B)   1985   ISRLFNMYRSYFGLVLVCFPVYSNPDSTEVYLLCLQKTVK....................
NiV(M)   1985   ISRLFNMYRSYFGLVLVCFPVYSNPDSTEVYLLCLQKTVK....................
HeV      1985   ISRLLNMYRSYFGLVLVCFPVYSNPESTEVYLICLQKTIK....................
MojV     1978   LTALFNMYKACFVDIKIALPKYSNPNSTECYLICTQPRIL....................
LayV     1978   LTALFNMYKACFVDIKIALPKYSNPNSTECYLICTQPRIL....................
GhV      1993   IMRLFKMYRSFFSLVIIAMPISSNPQSSEIYLICLQKLIR....................
PIV3     1950   WSKILYLYKLYWKDVSVISLKTSNPASTELYLISKDAYCT....................
SeV      1946   WTRQLSLYLRYWDEVNLIVLKTSNPASTEMYLLSRHPKSD....................
GSqRV    1946   WTRQLSLYMRYWNEVSLVVLRTSNPASTEMYLLSRSPKQD....................
MeV      1926   VQGFISYVGSHYREVNLVYPRYSNFISTESYLVMTDLKAN....................
RDV      1926   VQGFMSYMVPYYREVLIIYPRYSNFISTEAYLVLLGLRAN....................
CDV      1926   VQGFILYALPHFLRSFIVYPRYSNFVSTEAYLVFTGLRAG....................
BPV      1936   TSNLIQICYQCFSDVTIFIPASSNPHSTEVYLICRNKKIK....................
BeV      1913   TPQLLYLLLNCYEEVSIFIPSSSNPQSSEIYLISSCPRHK....................
PMPV1    1927   SQTLITLLYSYFSEVTCFIPSYSNPMSPECYLICTQKKFY....................
MMLPV1   1914   SFAIFDILQIYYEEVFCFIPMYSNPYSSEVYIISSVPRNT....................
RuV      1951   SPLFIRTCLEYYSDVNILIPKSSNPYSSEVYLCCQFLKMT....................
BaVV     1943   TLQLLTFIAEHYECCHLISPWYSSPESTEVYLIAKGLIQH....................
ASPV     1949   WHRVLVMLIKYWGVVQMISLRTSNPSSMEVYVVCSGKNQR....................
FLDV     1929   WTQIISLYLETFTNVTILCLKNSNPASSELYVICDGVKTR....................
APV      1926   FHLLINLFFPCSTKCYVVSNGYACRGDLECYLIFVMGYTG....................
PPMV1    1926   FHLLMNLFTPCSTKGYILSNGYACRGDMECYLVFVMGYLG....................
NDV      1926   FHLLMNLFTPCSTKGYILSNGYACRGDMECYLIFVMGYLG....................
APV17    1928   FNLMLNLLAPCSKSIRIISNGYAVRGDFECYIVATFGPTG....................
HPIV2    1961   FSQLAGLLWCFFDRIHLIRSSYSDPHSHEVYLVCRLAADF....................
MuV      1962   FSFLLTILWQFSSTIRILRSSYSDPNNHEVYIIATLAVDP....................
PIV5     1956   FSTLITFLWCYFERITVLRSTYSGPANHEVYLICILANNF....................
WTSPV    1866   WRYVCAWIRVLCTNITMHASPLSGSSNGEFYVTGARRELSTDKLLTIE.....ALL...M
WHPV     1840   TKYMINWCNCQFQDVVLQRSPYSNPSNSEFYLICKKFTRS....................
GPV      1930   SQCLIHIIYNYFSEVTCFIPAYSNPCTPECYLICTQKKHH....................
FPaV     1923   TEFIIKTLQDYYENVSIFIPFFSNPYSSEIYIICRNLRME....................
HMPV     1826   KLP......FFVRSVATFIMQGSKLSGSECYILLTLGHHN....................
AMPV     1826   KLP......YFVRSIATYVMQGSKLSGSECYVLLTLGHHN....................
RSV      1982   FKL......DNITILKTYVCLGSKLKGSEVYLVLTIGPAN....................
MPV      1858   DLP......HGVRLLKCNICLGSKLSGSESYLLIGLGLSN....................
EBOV     1971   MLWLNDNLAPFFATGYLIKPITSSARSSEWYLCLTNFLSTTRKMPHQN.....HLS....
VSV      1805   EKNAVTILGPMFKTVDLVQTEFSSSQTSEVYMVCKGLKKLIDEPNP.DWSSINESWKNLY
RABV     1839   NYKAIQHLSRAFPSVTGFITQVTSSFSSELYLRFSKRGKFFRDAEYLTSSTLREMSLVLF
```

```
                    .         .         .         .
NiV(B)  2025  TIIPPQ.RVLEHSDLHDEVNDQGITSVIFKIKNSQSKQFHEDL................K
NiV(M)  2025  TIVPPQ.KVLEHSNLHDEVNDQGITSVIFKIKNSQSKQFHDDL................K
HeV     2025  TIIPPQ.KVLDHSYLSDEINDQGITSVIFKIKNIQSKQFHEDL................V
MojV    2018  TVVTPE.IILEHSIDKPEDCNKSVLNYILILKFRIGISFEDRGRSVCDGKIVGDMSHGIV
LayV    2018  TVVTPE.IILEHSVDKPEDCNKSVLNYILILKFRIGISFEDRGRSVIDDETEASFANSVN
GhV     2033  GIITPQ.RVLGASMGSTPDITLSFINLVEKIKMKNYLQMIKDD................I
PIV3    1990  IMEPSE.VVLSKLKRLSLLEENNLLKWIILSKKRNNEWLHHEI.................
SeV     1986  IIEDSK.TVLASLLPLSKEDSIKIEKWILIEKAKAHEWVTREL.................
GSqRV   1986  VIEDSK.TVINSLLPLSKDDSIEIEKWILIEKTKVRDWVAAEL.................
MeV     1966  RLMNPE.KIKQQIIESSVRTSPGLIGHILSIKQLSCIQAIV....G..GAV.........
RDV     1966  RLIDPE.RIKQQVMESGIRTIPGLVGHILSIKQLNCIQSQA....G..PAI.........
CDV     1966  RLINPE.GIKQQILRVGIRTSPGLVGHILSSKQTACVQSLH....G..PPF.........
BPV     1976  TLIYPE.ILKEMTPKSKIWEGIHISEIILNLKLKNDLSMRD..KLNYK............
BeV     1953  CLLTPD.MLMRKVSMKVSLQSDRIHNTVMNYKIKHHLELIK..KRKNY............
PMPV1   1967  SLLYPN.IILDNIKEGFRTRNIIISRNILDMKFSIRKEMCK..VRGLF............
MMLPV1  1954  SLVYPD.LLMKYLDKVGPSDIFELQNTIINFKLGKYLEKRA..TEKLY............
RuV     1991  VFRSPF.ELLRQINTNVEFGTLTLRENILNIKVGDYIRAKQ..IHKRY............
BaVV    1983  QIIDPN.LLYYRLMDREPDRDGRLSGWIADQKIRYHSEFRILNKMNPYNRV.........
ASPV    1989  VLLSGS.EVLSETMPLSVESAADIEDWLINTKTQSWKWLAREI.................
FLDV    1969  IRVKSI.VYAQCVHYLTEESQSRLEQRIIDAKLKAGEHLMSVI.................
APV     1966  GSTFVR.EVVRMAKDLVRRNGTLLHSKDERTLMEVFNAQFMRV.....QELTSL......
PPMV1   1966  GPTFVN.EVVRMAKTLIQQHGTLLSKSDEIKLTKLFTSQQHRV.....IDILSS......
NDV     1966  GPTFVH.EVVRMAKTLIQRHGTLLSKSDEITLTKLFTSQQRRV.....TDILSS......
APV17   1968  GQIFMR.EVLQTGKALTRQGGSILTYEDECKLNKLFEVQLTHT.....RGILQQ......
HPIV2   2001  RTIGFS.AALVTATTLHNDGFTTIHPDVVCSYWQHHLENVGRV.....GKVIDE......
MuV     2002  TTSSFT.TALNRARTLNEQGFSLIPPELVSEYWRRRVEQGQII.....QDCIDK......
PIV5    1996  AFQTVS.QATGMAMTLTDQGFTLISPERINQYWDGHLKQERIV.....AEAIDK......
WTSPV   1918  SFHYPL.NILSELEMAIKEDLIRISEQADA.......ARHN.................ML
WHPV    1880  GTINRG.TVSGQELIH..........EARDVYLRSKEKCVN...................
GPV     1970  NLIYPD.IILTGISYHNIARNIIISDNILDMKFNIHKEETD..NRCLY............
FPaV    1963  LFVYPD.YLLRKSNDYKISDLTQLKYKILNIKLQNFNNQRE..RLGYY............
HMPV    1860  NLPCHG.EIQNSKMKIAVCNDFYAAKKLDNK......SIEA...................
AMPV    1860  NLPCHG.EVQSSKLKLAVCNDFSIPRKVEVK......AVEA...................
RSV     2016  VFPVFN.VVQNAKLILSRTKNFIMPKKADKE......SIDA...................
MPV     1892  KLPVYS.EVLHSKLLLAECHQFHHPKYLDVS......GINT...................
EBOV    2022  ...CKQ.VILT.ALQLQI.........QRSPYWLSHLTQYADC.................
VSV     1864  AFQSSEQEFARAKKVSTYFTLTGIPSQFIPDPFVNIETMLQIF......GVPTG.....V
RABV    1899  NCSSPKSEMQRARSLNYQDLVRGFPEEIISNPYNEMIITLIDS......DVESF.....L
```

```
                                    .         .         .         .
NiV(B)  2068  KY....................YHIDQPFFVPTKITSDEQVLLQ.AGLKLNGPEILKSEI
NiV(M)  2068  KY....................YQIDQPFFVPTKITSDEQVLLQ.AGLKLNGPEILKSEI
HeV     2068  KH....................YQVEQPFFVPSHITCDEKLLMQ.AGLKMNGPEILKNEV
MojV    2077  EYKRSGSDRIELTGQVKANICDSDNITLINLIQGLTEDDKILLS.CGLSLNGPTICQKLL
LayV    2077  EYNRARLPHRPPHPRNFYRQGNAENLTLINLVSGLTEDDKVLLS.CGLSLNGPTVCQKLL
GhV     2076  RK....................SVREPEMLIPGKLTSKEKFLLS.IGFSANGPTLIESET
PIV3    2032  .........................KEGERDYGVMRPYHMALQI.FGFQINLNHLAKEFL
SeV     2028  .........................REGSSSSGMLRPYHQALQT.FGFEPNLYKLSRDFL
GSqRV   2028  .........................REGSSSSGMLRPYHQALQT.FGFEPNLYKLCRDFL
MeV     2010  .......................SRGDINPILKKLTPIEQVLIS.CGLAINGPKLCKELI
RDV     2010  .......................TRGEINPILRKLTSIERILIS.CGLTINGTKICSDVV
CDV     2010  .......................HAKSFNPHLQGLTSIEKVLIN.CGLTINGLKVCKNLL
BPV     2021  ........................NDYLESNLGSLTEKEKLLIS.FGFRTNGPKNVKAVC
BeV     1998  ........................GDYAKSDLKALTKPEKLLLS.VGFSVNGPKLVKALT
PMPV1   2012  ........................GDYLASDLTTLDETEKALMT.YGFQINGPKLIKQIT
MMLPV1  1999  ........................GNYSKSRLSNLSNIDKILIR.IGFSLNGPKLIKQLT
RuV     2036  ........................HGYERSDLTELTPEERLLLS.YGFQINGPRIIKKLI
BaVV    2033  .......................GQSNLDSWLGSLTSVEQALLT.VGFQLNGPKLIKRLV
ASPV    2031  .........................DVGDGKGKGLRSYHRGLIT.FGFSTNMETISSQFL
FLDV    2011  .........................NDL..KSGEKINQIESLLK.IGFSANMTTVARQFL
APV     2014  .........................PTPGLIRILQKNIDGALIE.AGGQPVRPLCRENLS
PPMV1   2014  .........................PLPRLMKYLRDNIDAALIE.AGGQPVRPFCAESLV
NDV     2014  .........................PLPKLMKLLRENIDAALIE.AGGQPVRPFCAESLV
APV17   2016  .........................CLPDLLRHLHTNIDMSLIE.AGGQPVRPSSSDMLA
HPIV2   2049  .........................ILDGLATNFFAGDNGLILR.CGGTPSSRKWLEIDQ
MuV     2050  .........................VISECVRDQYLADNNIILQ.AGGTPSTRKWLDLPD
PIV5    2044  .........................VVLGEDALFNSSDNELILK.CGGTPNARNLIDIEP
WTSPV   1953  MH.....................TQQEPT.....VTLGGSICMS.LGFRYSSLIVRQTYL
WHPV    1910  .........................LILTTNKKNTTPGNNELLR.NGWANSMTASR.DLF
GPV     2015  ........................GDYLKSDLNQLDDTEKLLLT.FGFQMNGPKIIKQMT
FPaV    2008  ........................SDYTKSQLYELCKIEKTLLS.MGFSVNGPKIIKQIC
HMPV    1894  .....................................NCKSLLSGLRIPINKKELN....
AMPV    1894  .....................................NCKSLLSGLRTPINRAELD....
RSV     2050  .....................................NIKSLIPFLCYPITKKGIN....
MPV     1926  .....................................NIKSLIPMLDYPITYNKIT....
EBOV    2051  ....................................DLHLSYIR.LGFPSLEKVL.....
VSV     1913  SH.......A.............AALKSSDRPADLLTISLFYMAII....SYYNINHIRV
RABV    1948  VH.......K.............MVDDLELQRGTLSKVAIIIAIMIVFSNRVFNVSKPLT
```

```
NiV(B)  2107  SYDI..GSDINTLRDTIIIMLNEAMNYF......DD...NRSPSHHLEPYPVL..ERTRI
NiV(M)  2107  SYDI..GSDINTLRDTIIIMLNEAMNYF......DD...NRSPSHHLEPYPVL..ERTRI
HeV     2107  GYDI..GSDINTLRSTIIILLNEAMNYF......DD...ERSPSHHLEPFPVL..EKTRV
MojV    2136  GYDV..SGPLETILESSFLLLNEALNIY......DG...NRMPSSFFEPYPIL..ESTRL
LayV    2136  GYDT..GSPLGTILESSFLLLNEALNIY......DG...NRMPSSFFEPYPIL..ESTRL
GhV     2115  GHDP..GSTKEDLRTALIITLNELLNHL......DL...ERHPSSFFQPYQVL..ENSRI
PIV3    2066  STPD..LTNINNIIQSFQRTIKDVLFEWINITHDDK...RHKLGGRYNIFPLK..NKGKL
SeV     2062  STMN..IADTHNCMIAFNRVLKDTIFEWARITESDK...RLKLTGKYDLYPVR..DSGKL
GSqRV   2062  STLN..IADTHNCMNSFGRTLKDTIFEWSRITESDK...RMKLTGKYDLYPVR..DSGKL
MeV     2046  HHDV..ASGQDGLLNSILILYRELARFK......DN...QRSQQGMFHAYPVL..VSSRQ
RDV     2046  HHDI..SSGPDGLLNSSIILLQELARFK......DN...QRSQQGMFHAYPVL..LSSRQ
CDV     2046  HHDI..SSGEEGLKGSITILYRELARFK......DN...HQSSHGMFHAYPVL..IASQE
BPV     2056  GHDV..ASGSDNLVSSINLTLNHILNSY......DI...KRDQVPFFDPYPIL..DSSKL
BeV     2033  GHDV..ASGQDILRSSITSLIIDTINEY......DL...ERSHTPFFDPYPIR..RSSKH
PMPV1   2047  GHDI..GSGAPNLRGYINSSVNNLINYC......DP...DRQNSNFLDAYPLN..RDSKL
MMLPV1  2034  GHDV..GTGSHSLVSSIQLLANSLVTYL......DP...ERDKSYFFDPYPLK..MDSKV
RuV     2071  NHDI..AGGKSVLQQLIKSGFYSLAVEL......DD...PLRHDHFFSPYPIR..VNTKY
BaVV    2069  SFDP..GSDLESLKSAIGMAYKELLYNY......LK...SNQEHHFFQPYPIL..ESSKV
ASPV    2065  GVGD..LTDLERVTTLFCSTMEQTFRSMEDRSENLS...GALMMGKFDVYPVR..DEGKR
FLDV    2043  RVTD..LTDTTTIFTKLRLALVDLLRVTS......E...ESTTLSLYQPYPLS..QSGKC
APV     2048  PACFDATKATDIIASHIDTAI....RSVIYIEEGGA...LADTVFLFTPYNLS..VEGKK
PPMV1   2048  GTLTDMTQVAQIIASHIDTVI....RSVIYMEAEGD...LADTVFLFTPYNLS..TDGKK
NDV     2048  STLIDMTQTTQIIASHIDTVI....RSVIYMEAEGD...LADTVFLFTPYNLS..TDGKK
APV17   2050  SVQIGDLTRDEVIIQYIDTSL....KTVAYLDSSDE...LADTVFVLTPFNLS..ARGKC
HPIV2   2083  LASF..DLVQDALVTLITIHL....KEIIEVQSSHT...EDYTSLLFTPYNIG..AAGKV
MuV     2084  YLSF..NELQSEMARLITIHL....KEVIEILKGQS...SDHDTLLFTSYNVG..PLGKI
PIV5    2078  VATF..IEFEQLICTMLTTHL....KEIIDITRSGT...QDYESLLLTPYNLG..LLGKI
WTSPV   1986  GWLG..TDDRKG......I.......AWKHIKNETK...HDLRGNRLKAYGDT..KDAKI
WHPV    1943  GNLM....DKHEIP......THSLERE.............VLTEHGMSGWAKK..KMTRI
GPV     2050  GHDV..GSGSSTLKAYINSSVNNIINYC......DP...DRSKSNFLDPYPLL..AESKI
FPaV    2043  GHDV..ATGSANLLASINLNVNSLVNFV......DP...NREKNVFLDPYPLL..DSSKV
HMPV    1913  .............RQR....RLLT.LQ................SNHSSVATVGGS
AMPV    1913  .............RQK....KMLT.LR................SYHSSVATVGGS
RSV     2069  .............TAL....SKLKSVVS...............GDILSYSIAGRN
MPV     1945  .............TLL....ESVRELSS...............NKNKNTMWIGRN
EBOV    2069  YHRYNLVDSKRGPLVSITQHLAHLRAEIRELTN.DY...NQQRQSRTQTYHFIRTAKGRI
VSV     1949  GPIPP.NPPSDGIAQNVGIAITGISFWL.SLMEKDIPLYQQCLAVIQQSFPIR....WEA
RABV    1988  DPSFY.PPSDPKILRHFNICCST.MMYL.STALGDVPSFARLHDLYNRPITYY....FRK
```

```
NiV(B)  2154  KTIMNRVTKKVIVY.......SLIKFK.....DTKSSELYHIKNNIRRKVL....ILDFR
NiV(M)  2154  KTIMNCVTKKVIVY.......SLIKFK.....DTKSSELYHIKNNIRRKVL....ILDFR
HeV     2154  KTIMGRVTRKVTVY.......SLIKLK.....ETKSPELYNIKNYIRRKVL....ILDFR
MojV    2183  TRYMHKFVRKIIGY.......TYLKYN.....TLDLSLVNQHISNLKRRYL....IFDFN
LayV    2183  SKYMHKFIRKIIGY.......TYLKYD.....SLDLSLVDRHISNLKRRYL....IFDFS
GhV     2162  LSLKDKIAKKYALF.......ILLYIN.....TVESK..RKVICNLKRGKI....VCDFG
PIV3    2119  RLLSRRLVLSWISL.......SLSTRL.....LTGRFPDEKFEHRAQTGYV....SLADT
SeV     2115  KTVSRRLVLSWISL.......SMSTRL.....VTGSFPDQKFEARLQLGIV....SLSSR
GSqRV   2115  RVISRRLIISWISL.......SMSTRL.....VTGSFPDHKFEARLQLGIV....SLSSK
MeV     2093  RELVSRITRKFWGH.......ILLYSG.....NRK..LINRFIQNLKSGYL....VLDLH
RDV     2093  RELISRVARKFWGY.......ILLYSS.....DRR..LLSRLVSNLKSGYL....LFDLH
CDV     2093  RELVSIIAKKYCGY.......ILLYSG.....DLY..EITRIVRNLKANHI....IFDLH
BPV     2103  REQVELLTKKISVY.......YILYSE.....GNNLQERCKIIRQLRRKSL....MMDFN
BeV     2080  RERMQIVCRKIVAY.......FILYSP.....PVHSEVRREIIKSLRKKLL....IIDLK
PMPV1   2094  REYIDLLGKKLAVY.......ILLYMK.....DSDQDLRRSLINNLRRKYI....YLDIC
MMLPV1  2081  KEMIESISIKIIIY.......YIVYAQ.....PKSNQQRSRYINCLRKQYL....YHDLT
RuV     2118  NLIIKDLFYKIGVF.......IILYLK.....YPNYSIRNHLISMLRRKIL....AININ
BaVV    2116  REIVYDIIRKXSVY.......XLLDGK.....ANRINFACHLAQSISKGHI....IFSLY
ASPV    2118  RERIKNLILKWIAL.......NLSTFF.....VHRRFTVGSFEKQCSVGCI....NMDID
FLDV    2090  RVVKERAMKGWISI.......NLNTIF.....TKRNYLKHDFEYLAVRNKV....IIDMK
APV     2099  LTSLTQCTRQIFDM.......ILLS.....ITKEDQIKISEMMGIVLKGTVCLEDLIPLR
PPMV1   2099  RTSLKQCTRQILEV.......TILG.....LRARDLNKVGDIIGLILRGMVSLEDLIPLR
NDV     2099  RTSLKQCTKQILEV.......TILG.....LRAKDINKVGDVISLVLRGAVSLEDLIPLR
APV17   2101  HTVLSTCTKHVFEL.......RLLQ.....VSKTDLHGIEQLLSIIIQGHVALHDLILLR
HPIV2   2132  RTIIKLILERSLMY.......TVRNWLVLPSSIRDSVR.....QDLELGSFRLMSILSEQ
MuV     2133  NTILRLIVERILMY.......TVRNWCILPTQTRLTLR.....QSIELGEFRLRDVITPM
PIV5    2127  STIVRLLTERILNH.......TIRNWLILPPSLRMIVK.....QDLEFGIFRITSILNSD
WTSPV   2026  RSEIKEEFRRSQVE.......LLISTD..........LRHRIITRTEIAEITFNNLSDSK
WHPV    1978  REEIILRMRDMIII.......RIMNTS.....RAKIAEWETIP..........EDIRTVT
GPV     2097  RELLDNVCKKICVY.......TLLYMK.....DNYKHIRRNLINCIRRKYL....RVDIR
FPaV    2090  REMVISIGKKSLIY.......LFIYMK.....NKDKRIYKYHMDNLRKQSM....VIDFT
HMPV    1934  KVIES....KWLTN.............K.....ANTIIDWLEHILNSPKGEL......NYD
AMPV    1934  RVIES....KWLSK.............K.....ATTIIEWLEHILNSPKGEL......NYD
RSV     2092  EVFSN....KLINH.............K.....HMNILKWFNHVLNFRSTEL......NYN
MPV     1968  PVYHN....KWLKR.............K.....YFNILKWLKYCIELPAFRM......DYN
EBOV    2125  TKLVNDYLKFFLIV.......QALKHN.....GTWQAEFKKLP...............ELI...
VSV     2003  VSVKGGYKQKWSTRGDG.LPKDTRTSDSLAPIGNWIRSLELVRNQVRL...........NPFN
RABV    2041  QVIRGNVYLSWSWSNDTSVFKRVACNSSLSLSSHWIRLIYKIVKTTRL...........VGSI
```

```
NiV(B)   2198  SKLMTKTLPKGMQERREKSG....FKEVWIVDLSNREVKIWWKIIGYISII.........
NiV(M)   2198  SKLMTKTLPKGMQERREKNG....FKEVWIVDLSNREVKIWWKIIGYISII.........
HeV      2198  SHTMIKLLPKGMKERREKSG....FKEIWIFDLSNREVKIWWKIIGYLSLV.........
MojV     2227  TYKHTKIIPNYYYKRINKGK....FKQNWMISLTTKEIKLWWKLISYVPIFNKTT.....
LayV     2227  DYKHTKIIPNYYYKRIKKGK....FKQSWMISLATKEIKLWWKLISYVPVFRKIV.....
GhV      2204  EKQFLKILPKKLRERVHTAL....ENSIHIINLERVIQKRWWKIVGYTGIL.........
PIV3     2163  DLESLKLLSKNIIKNYKECI......GSISYWFLTKEIKILMKLIGGAKLLGIPRQYK..
SeV      2159  EIRNLRVITKTLLDRFEDII......HSITYRFLTKEIKILMKILGAVKMFGARQNEY..
GSqRV    2159  EIRSLRVVSKTIIDRFEGII......HSLTYRFLTKEIKILMKILGSVKMFGARQNEY..
MeV      2135  QNIFVKNLSKSEKQIIMTGG....LKREWVFKVTVKETKEWYKLVGYSALIKD.......
RDV      2135  QNLFMKNLSKSEKQLIRTGG....LKREWLFKLTTKEIKEWFKLIGYSALIRE.......
CDV      2135  RNLFMDNLSRSDRSLILTTI....PKKNWLFQLETKEIKEWFKLLGYSALIRNH......
BPV      2147  NPFVENLVSSRLIKRFKEFG....LKRHWVFQIPTVEIKMWWKVIGYSLLVLP.......
BeV      2124  DNSCKIFMYKKLREKYARTG....LSHNVLIQLETSEVKIWWKILGYSLMTNQDNES...
PMPV1    2138  NYSFRGLIQPYLMKKLIKLD....LNTNWMYQLDTPEVKIWWKIVGYSVLHQEDD.....
MMLPV1   2125  DEQCNTVVPSYLIRKLKKTG....YQLDWKEDLDAGSIKAWWKIVGYSMLIDEQ......
RuV      2162  NDDISQLIDKKSKIILIKQG....IELDFNHKLETKEIKQWWKIVGYSLIYHED......
BaVV     2160  NKSDTSLCPHKVYNKLIRTS....FQKIWIMDLTTAEIKIWWKILGYTYLI.........
ASPV     2162  TVYKSGLLTKSMYKRFNKVL......RRCAYTFPTRDIKKLMKLVGSVKFMGAT......
FLDV     2134  CLLACGVVNKKTGDRLNRMS......HLLYSEITNRDKKKLFKLIGSAFYFSGR......
APV      2147  MYLKRSTCPKYLKEVLGMQKLRELFKTTSRFVLSRSTQKYYMKIIGNAIKGYYSMI....
PPMV1    2147  TYLKHSTCPKYLKTVLGITKLKEMFTETSLICLTRAQQKFYMKTIGNAAKGYYNNNDS..
NDV      2147  TYLKRSTCPKYLKAVLGITKLKEMFTDTSLLYLTRAQQKFYMKTIGNAAKGYYSNNDS..
APV17    2149  SYLRGSQCPKYLLDSFGKIRLREFFEEMSRFTISRSLQKLYLKTLGNAIKGYLTA.....
HPIV2    2180  TFLKKTPTKKYLLDQLTRTYISTFFNSHSVLPLHRPYQKQIWKALGSVIYCSETVDIP..
MuV      2181  EILKLSPNRKYLKSALNQSTFNHLMGETSDILLNRSYQKRIWKAIGCVIYCFGLLTPD..
PIV5     2175  RVLKLSPNRKYLITQLTAGYIRKLIEGDCNIDLTRPIQKQIWKALGCVVYCHDPVDQR..
WTSPV    2069  KQ....MCI...DKLSKLP........LLTHLEMKHIYKSWTVYKINQL...TPQDH..
WHPV     2016  RNEFLKGITKKVAKRIS.......IKESTTVEIDWSFQKLLMK.......CHPE......
GPV      2141  SESVKSLIQVYLIKKLSKLS....INMDWSYQLSTAEVKIWWKIVGYSVIHTET......
FPaV     2134  SDLYKVCIYKHLYNKLMNSG....IKQILVLQMTSVEVKIWWKIIGYSFLTVEN......
HMPV     1967  FFEALENTYPNM..............IKLIDNLGNAEIKKLIKVTGYMLVSKK.......
AMPV     1967  FFEALENTYPNM..............VKLLDNLGSAELKKLIKVTGYMLMSKK.......
RSV      2125  HLYMVESTYPYL..............SELLNSLTTNELKKLIKITGSLLYNFHNE.....
MPV      2001  SFERIEMLYPNL..............RDLVDSVSTSELKKVIKVTGILFRSNTM......
EBOV     2159  .....SVCNRFYHIRDCNCEERFLVQTLYLHRMQDSEVKLIERLTGLLSLFPDGLYRF..
VSV      2054  EILFNQL.........CRTV.........DNHLKWSNLR...RNTGMIEWINRRISKEDR
RABV     2093  KDLSREV.........ERHL.........HRYNRWITLEDIRSRSSLLDYS.........
```

```
NiV(B)         ..................................
NiV(M)         ..................................
HeV            ..................................
MojV           ..................................
LayV           ..................................
GhV            ..................................
PIV3     2215  ......EP.....EEQLLENYNQHDEFDID....
SeV      2211  ......TTVIDDGSLGDIEPYDSS..........
GSqRV    2211  ......STVIEDGSLDDIEGYVDD..........
MeV            ..................................
RDV            ..................................
CDV            ..................................
BPV            ..................................
BeV            ..................................
PMPV1          ..................................
MMLPV1         ..................................
RuV            ..................................
BaVV           ..................................
ASPV           ..................................
FLDV           ..................................
APV            ..................................
PPMV1          ..................................
NDV            ..................................
APV17          ..................................
HPIV2    2238  ........LIKDIQIEDINDFE.DIERGIDGEEL
MuV      2239  ........VE.DSERIDIDN.D.IPDYDIHGDII
PIV5     2233  ........ES..TEFIDININE.EIDLGIDGEEI
WTSPV    2108  ........RTLDLDWTDSEPED..EPWD......
WHPV           ..................................
GPV            ..................................
FPaV           ..................................
HMPV           ..................................
AMPV           ..................................
RSV            ..................................
MPV            ..................................
EBOV     2212  ......D...........................
VSV      2093  SILMLKSDLHEENSWRD.................
RABV     2126  .CL...............................
```
